# Template-independent enzymatic synthesis of RNA oligonucleotides

**DOI:** 10.1038/s41587-024-02244-w

**Published:** 2024-07-12

**Authors:** Daniel J. Wiegand, Jonathan Rittichier, Ella Meyer, Howon Lee, Nicholas J. Conway, Daniel Ahlstedt, Zeynep Yurtsever, Dominic Rainone, Erkin Kuru, George M. Church

**Affiliations:** 1 Department of Genetics, Harvard Medical School, Boston, MA USA; 2 Wyss Institute for Biologically Inspired Engineering, Boston, MA USA; 3 EnPlusOne Biosciences Inc., Watertown, MA USA

**Keywords:** Nucleic-acid therapeutics, Synthetic biology

## Abstract

RNA oligonucleotides have emerged as a powerful therapeutic modality to treat disease, yet current manufacturing methods may not be able to deliver on anticipated future demand. Here, we report the development and optimization of an aqueous-based, template-independent enzymatic RNA oligonucleotide synthesis platform as an alternative to traditional chemical methods. The enzymatic synthesis of RNA oligonucleotides is made possible by controlled incorporation of reversible terminator nucleotides with a common 3′-*O*-allyl ether blocking group using new CID1 poly(U) polymerase mutant variants. We achieved an average coupling efficiency of 95% and demonstrated ten full cycles of liquid phase synthesis to produce natural and therapeutically relevant modified sequences. We then qualitatively assessed the platform on a solid phase, performing enzymatic synthesis of several *N* + 5 oligonucleotides on a controlled-pore glass support. Adoption of an aqueous-based process will offer key advantages including the reduction of solvent use and sustainable therapeutic oligonucleotide manufacturing.

## Abstract (web summary)

Single-stranded RNA is enzymatically synthesized without use of a template strand.

## Main

Synthesis of RNA oligonucleotides by the phosphoramidite chemical method has enabled many valuable discoveries and new ways to treat disease throughout the past 50 years^1–4^. This has culminated in the development of an array of therapeutic modalities that include antisense oligonucleotides (ASOs) and short interfering RNA (siRNA)^5–7^. ASOs and siRNA have traditionally been used to treat rare diseases such as spinal muscular atrophy and hereditary transthyretin-mediated amyloidosis^8–10^. They are often chemically modified, which offers therapeutic advantages such as increased binding affinity, stability, and protection from nuclease degradation^4,11^. More recently, the *N*-acetylgalactosamine (GalNAc) ligand conjugated to siRNA enabled tissue-specific delivery of the active RNA drug^12,13^. These advances have resulted in unparalleled growth in the oligonucleotide therapeutics field. There is now immense demand for large-scale RNA manufacturing, which has presented new challenges to current production capacities^14–16^. This is especially pertinent as RNA oligonucleotides have become an increasingly viable treatment option for cardiovascular disease and hypertension, which both have large patient populations^17,18^.

Chemical phosphoramidite synthesis faces many hurdles that currently hinder large-scale manufacturing of RNA oligonucleotide therapeutics. First, scalability remains a key issue, as both batch size and overall throughput are limited by the need to store, handle and dispose of large quantities of flammable organic solvents^19–21^. To chemically synthesize oligonucleotides, facilities must be explosion proof and are generally subject to strict regulatory oversight owing to the high hazards associated with the process^15,22,23^. In addition, chemical phosphoramidite synthesis is known for its poor atom economy and high process mass intensity^19^, where thousands of kilograms of raw material input is generally needed to yield just a few kilograms of RNA oligonucleotide therapeutic product^14,20,24^. Both atom economy and process mass intensity are driven in part by the many protecting groups needed to ensure RNA oligonucleotide survival during chemical synthesis^19^. Taken together, these issues create critical bottlenecks for large-scale manufacturing of oligonucleotides and may limit the future potential of RNA therapeutics.

Enzymatically synthesizing oligonucleotides, rather than using traditional chemical methods, holds the potential to meet anticipated demands for high-quality and diverse RNA^25–28^. Adoption of enzymatic methods may offer RNA oligonucleotide production with high yield and purity owing to simplified downstream purifications and better atom economy. An aqueous-based process can also eliminate the large-scale consumption of organic solvents and prevent generation of hazardous waste, thereby reducing the overall environmental impact of oligonucleotide synthesis^19^. Here, we describe the development of a water-based enzymatic synthesis platform with the capacity to write natural and modified RNA oligonucleotides one base at a time without the need for a template sequence. With an improved atom economy and aqueous reaction conditions, our enzymatic process has considerable upside for manufacturing RNA therapeutics in a sustainable manner.

## Results

### Enzymatic RNA oligonucleotide synthesis overview and cycle

**Fig. 1:**
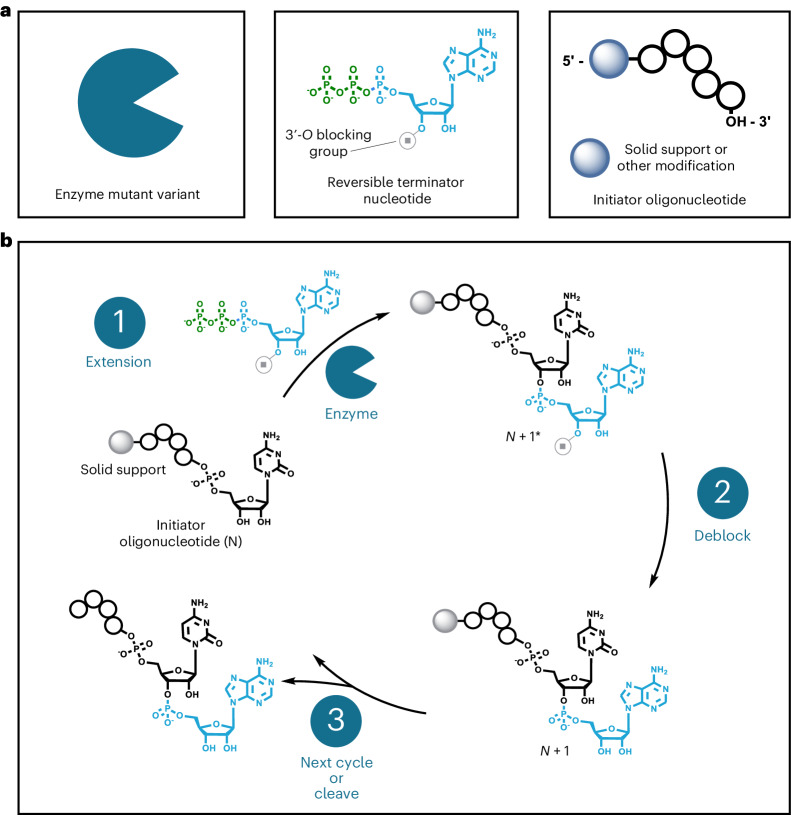
General overview of the controlled, template-independent enzymatic RNA oligonucleotide synthesis process. **a**, Three primary components are required for carrying out an enzymatic extension: 3′-blocked reversible terminator nucleotides, enzymes capable of their robust and indiscriminate incorporation, and an initiator oligonucleotide. The reversible terminator group stops uncontrolled polymerization by the enzyme and limits extension to a single incorporation event. The initiator oligonucleotide may vary in terms of sequence and length. It can also be bound to a solid support or feature other modifications such as a 5′-fluorophore or functional handle. **b**, A typical cycle of enzymatic synthesis begins with (1) extension of the initiator oligonucleotide in the presence of an RT-NTP and enzyme. A deblocking step (2) then occurs to remove the reversible terminator group from the extended oligonucleotide, allowing the next cycle of synthesis to commence. When the desired length and composition have been reached, the final oligonucleotide product is isolated. Source data

Our platform synthesizes RNA oligonucleotides over a series of iterative reaction cycles in the liquid bulk phase or on a solid support in a controlled, template-independent manner. Synthesis occurs in the 5′-to-3′ direction and requires reversible terminator nucleoside triphosphate (RT-NTP) building blocks, an enzyme capable of their efficient incorporation, and a pre-existing oligonucleotide to initiate controlled synthesis (Fig. 1a). We use mutant variants of CID1 poly(U) polymerase (PUP) derived from the fission yeast *Schizosaccharomyces pombe* to write RNA oligonucleotides^29–31^. Our PUP mutants show increased incorporation efficiency and promiscuity compared with their wild-type counterpart (Supplementary Fig. 1). Deoxynucleotide triphosphates can be incorporated by our PUP mutants; however, their use is currently limited to single terminal extension reactions (Supplementary Fig. 20a). The initiator oligonucleotide, which is essential for enzymatic functionality and controlled, template-independent synthesis, should be at least 10 nucleotides (nt) in length and can be either a homopolymeric string of bases or a rationally designed sequence (Supplementary Figs. 2 and 3). PUP prefers initiators composed primarily of RNA bases, but we have found that DNA can be used if at least a single 2′-OH group base is present at the 3′ terminus (Supplementary Fig. 20b).

An enzymatic reaction cycle is similar to chemical phosphoramidite synthesis^32^, except that there are only two main steps: extension and deblocking (Fig. 1b). During an extension step, the desired RT-NTP is enzymatically incorporated by PUP onto the 3′ terminus of the initiator oligonucleotide. A successful extension step results in generation of an *N* + 1* product, where *N* and the asterisk represent the length of the initiator and presence of a reversible terminator blocking group, respectively. Next, the *N* + 1* oligonucleotide undergoes deblocking, in which the reversible terminator group is removed under mild conditions, yielding an unblocked *N* + 1 product and allowing the subsequent cycle of controlled, enzymatic synthesis to commence. The process of iterative extension and deblocking is repeated until the desired full-length RNA oligonucleotide has been synthesized. The product can then be released enzymatically from the initiator or solid support for isolation. Unlike chemical-based RNA oligonucleotide synthesis, our enzymatic method does not require a final global deprotection step^33^.

### Development of ideal reversible terminator nucleotides

The key to building our enzymatic RNA oligonucleotide synthesis platform was the development of NTPs with a reversible terminator blocking group that can be efficiently removed without the formation of reactive side products. An ideal blocking group is one that is stable during enzymatic extension reactions and under long-term storage conditions^34^. It should also be a small moiety to ensure efficient nucleotide incorporation onto the growing oligonucleotide during synthesis. Although several viable options including nitrobenzyl, aminoxy (–ONH_2_), azido methyl ether (–OCH_2_N_3_) and phosphate (–PO) were initially considered because they have been previously used to control enzymatic polymerization^35–37^, we ultimately decided that an allyl ether (–OCH_2_CHCH_2_) blocking group best met our criteria. This choice was further supported by work demonstrating quantitative allyl ether deblocking using palladium as a catalyst and triphenylphosphine trisulfonate (TPPTS) in buffered aqueous solutions^38–40^. The versatility and selectivity of Pd as a catalyst has enabled the manufacturing of many pharmaceuticals and fine chemicals at kilogram scale^41^.

**Fig. 2:**
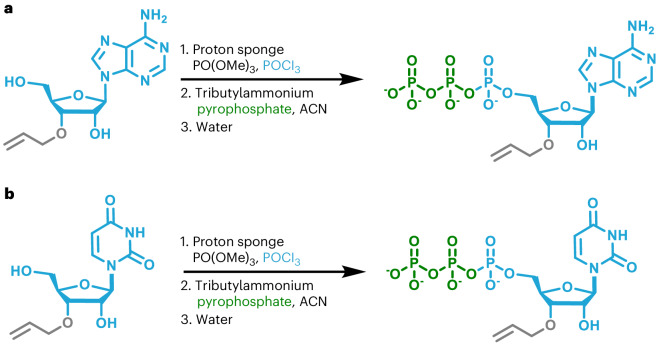
Preparation of 3′-*O*-allyl ether ATP and UTP. **a**, Preparation of 3′-*O*-allyl ether ATP. **b**, Preparation of 3′-*O*-allyl ether UTP.

**Fig. 3:**
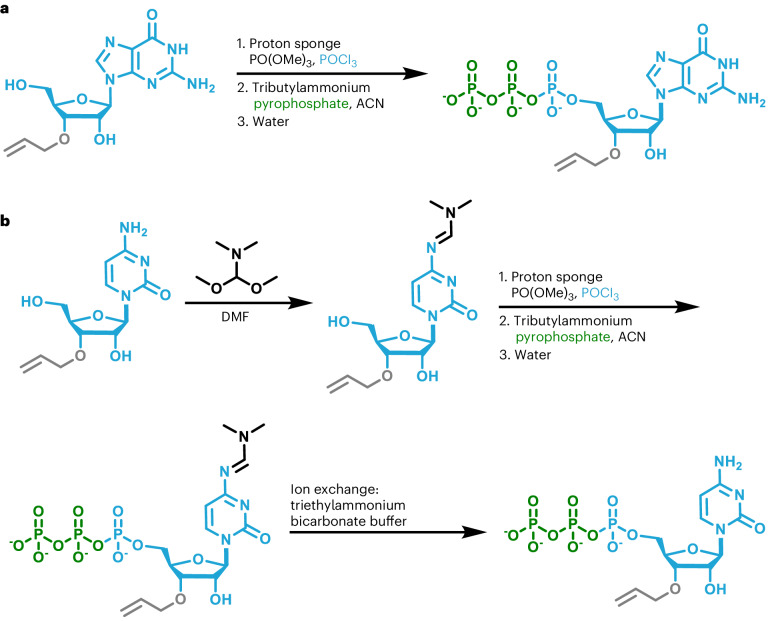
Preparation of 3′-*O*-allyl ether GTP and CTP. **a**, Preparation of 3′-*O*-allyl ether GTP. **b**, Preparation of 3′-*O*-allyl ether CTP.

Next, we needed to decide where to install the allyl ether blocking group on the NTP. As PUP catalyzes uncontrolled polymerization of long homo- and heteropolymers in the presence of NTPs with a free 3′-hydroxyl (–OH), installing the allyl ether group at this position was crucial to limiting extension to a single incorporation. Blocking at the 3′-sugar position would also enable the use of 2′-modifications such as 2′-fluoro (–F), 2′-methoxy (–OMe) and 2′-methoxyethyl (–MOE), which are important to the functionality of many therapeutic and antisense oligonucleotides^42^. Thus, we accessed the complete set (A, U, G, C) of 3′-*O*-allyl ether RT-NTPs using established methods for nucleoside preparation and triphosphorylation (Figs. 2 and 3). As our enzymatic reaction conditions were substantially milder than those used for chemical phosphoramidite synthesis, we did not need to protect the base or phosphate of the NTP with acetyl, benzoyl or 2-cyanoethyl groups^43^. In addition, traditional chemical methods require protection of the 2′-OH with bulky groups such as *t*-butyldimethylsilyl or triisopropylsilyloxymethyl. Deprotection of the 2′-OH is often a source of impurities or damaged oligonucleotide product that must be purified away^1,9,35,36,44,45^. Our enzymatic approach allows us to leave the 2′-OH of RT-NTPs unprotected. The absence of base, phosphate and 2′-OH protecting groups eliminates the need for global deprotection.

### Initial evaluation of 3′-*O*-allyl ether RT-NTPs

**Fig. 4:**
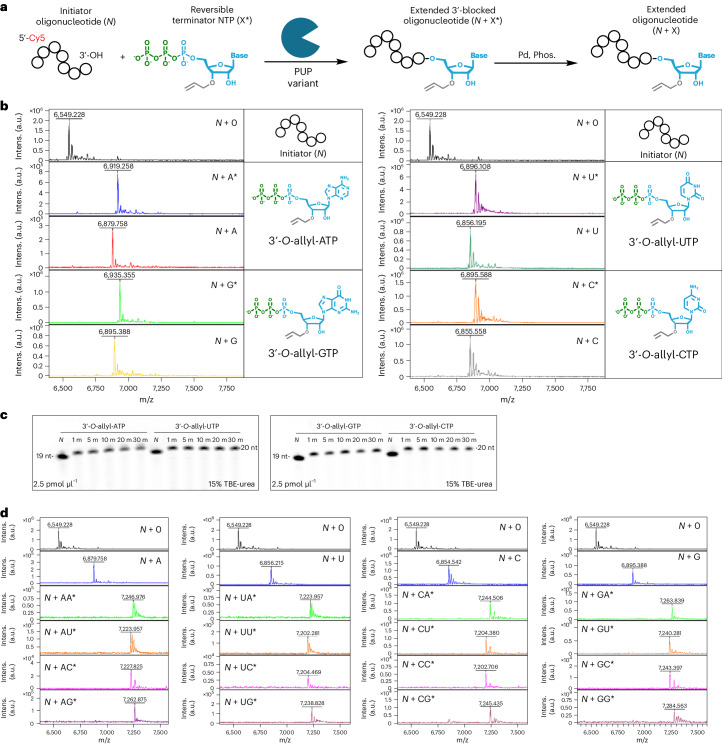
Initial evaluation of 3′-*O*-allyl ether RT-NTPs as building blocks for controlled, enzymatic RNA oligonucleotide synthesis. **a**, A complete set (A, U, G, C) of 3′-*O*-allyl ether NTPs were tested for enzymatic incorporation and deblocking using a liquid bulk phase reaction scheme, where *N* is the length of the initiator, *N* + 1* is the extension intermediate with the 3′-*O*-allyl ether group as represented by the asterisk, and *N* + 1 is the deblocked product for each base. **b**, MALDI-TOF mass spectrometry was used to verify NTP extension to *N* + 1* by the poly(U) mutant variant and subsequent deblocking of the allyl ether group to *N* + 1; the masses of all resultant oligonucleotides are given and compared with that of the 19-nt initiator. **c**, Kinetic profile for each 3′-*O*-allyl ether NTP, obtained and analyzed with denaturing gel electrophoresis; reaction samples were taken at 1, 5, 10, 20 and 30 min. Control reactions (N) included all reaction components except NTP. This direct comparison was performed once but is a compilation of several independent experimental repeats with similar results. **d**, MALDI-TOF was used to assess the efficiency of two controlled, enzymatic synthesis cycles in which all *N* + 2* combinations of base extensions were produced; the masses of all resultant oligonucleotides are given and compared with that of the 19-nt initiator. The observed and calculated *m*/*z* values for all oligonucleotide synthesis products generated by MALDI-TOF analysis, as well as their respective theoretical molecular weights, are summarized in Supplementary Table 1. Phos., triphenylphosphine; intens., intensity. Source data

Following a liquid bulk phase reaction scheme (Fig. 4a), we evaluated the capacity of our 3′-*O*-allyl ether NTPs to undergo a single cycle of enzymatic RNA synthesis. Post-reaction analysis of enzymatic extension using matrix-assisted laser desorption/ionization-time of flight (MALDI-TOF) mass spectrometry showed the absence of initiator oligonucleotide and presence of the desired *N* + 1* product for each RT-NTP (Fig. 4b). These results were supported by liquid chromatography mass spectrometry (LC/MS), which showed an average coupling efficiency of 95% for each 3′-*O*-allyl-NTP (Supplementary Fig. 4). MALDI-TOF and LC/MS also confirmed the removal of the allyl ether group upon deblocking in all cases (Fig. 4b and Supplementary Fig. 5). Time-course analysis showed that extension reactions were completed within the first minutes of incubation (Fig. 4c); however, enzymatic turnover was primarily driven by nucleobase type. Both PUP mutants had the highest turnover for 3′-*O*-allyl-UTP and -CTP, followed by 3′-*O*-allyl-ATP, then 3′-*O*-allyl-GTP (Supplementary Fig. 6). Although the initiator concentration could be as high as 50 pmol µl^−1^ for specific bases, these results reaffirmed that our standard reaction conditions (2.5 pmol µl^−1^ initiator) were sufficient to promote high coupling efficiencies for all tested RT-NTPs.

In assessing the product impurity profiles of our initial extension and deblocking reactions using our 3′-*O*-allyl ether NTPs, we found the majority to be buffer components and additives used in the extension and isolation steps, respectively. In general, these impurities absorbed strongly at 260 nm and eluted well before the extended or deblocked oligonucleotide product, as shown by LC, indicating good overall isolated purity in all cases (Supplementary Figs. 4 and 5). Notably, we observed the formation of a minor side product after enzymatic extension with 3′-*O*-allyl-ATP (Supplementary Fig. 4b). Given that our NTP starting material was of high purity, and spontaneous isomerization of the 3′-*O*-allyl ether blocking group is profoundly unlikely owing to its exceptional stability, we believe this side product to have been a double extension *N* + A*A* oligonucleotide with a 2′-,5′-phosphodiester linkage that also carried through deblocking to form *N* + AA (Supplementary Fig. 5a). However, further characterization is warranted, as the formation of such a linkage by PUP is unexpected.

### Multicycle synthesis of natural RNA oligonucleotides

Having characterized our RT-NTP building blocks, we next sought to prove that multicycle enzymatic synthesis of longer RNA oligonucleotides was possible with our platform. To do this, we first generated oligonucleotide extension products that constituted all 16 possible *N* + 2* base transitions (for example, A to A, G to U, and so on) under standard extension and deblocking reaction conditions. We have previously found that certain base transitions can be troublesome for template-independent polymerases^46^; however, analysis with MALDI-TOF confirmed the formation of all intended products, as evidenced by the total consumption of the initiator oligonucleotide and deblocked *N* + 1 during the first and second cycles of synthesis, respectively (Fig. 4d). All *N* + 2* base transitions were achieved at high efficiency without the need to alter any reaction components or increase incubation times for extension or deblocking steps.

**Fig. 5:**
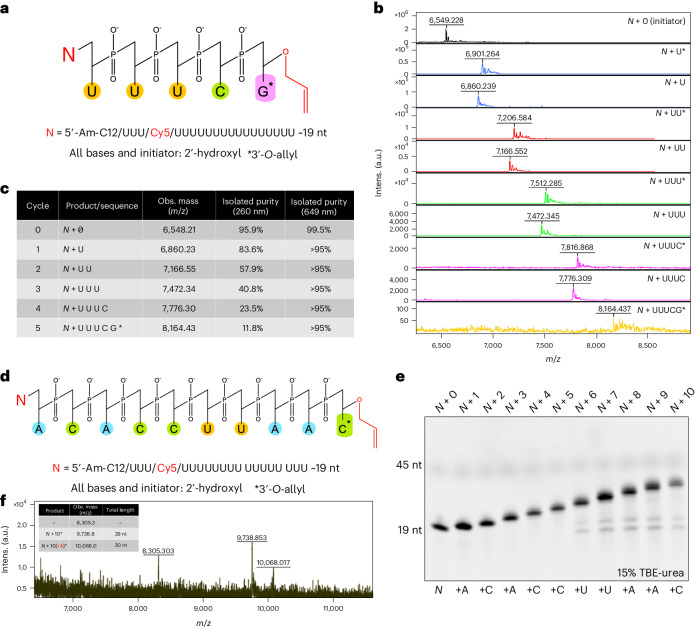
Results of multicycle enzymatic synthesis to produce natural RNA oligonucleotides using the 3′-*O*-allyl ether NTP set. **a**, An *N* + 5 RNA oligonucleotide with the sequence *N* + U-U-U-C-G* was produced in the liquid bulk phase, where the asterisk represents a 3′-*O*-allyl ether group. **b**, MALDI-TOF mass spectrometry was used to track the outcome of the extension and deblocking steps during each cycle of enzymatic synthesis. **c**, The isolated purity of the growing oligonucleotide and final product was determined after each cycle using LC/MS at 260 nm and 649 nm; the results are summarized in the table. **d**, An *N* + 10 RNA oligonucleotide with the sequence *N* + A-C-A-C-C-U-U-A-A-C* was also produced in the liquid bulk phase. **e**, High-resolution gel electrophoresis was used to analyze the success of each cycle after the sequence had been enzymatically synthesized with an imager set to collect the Cy5 signal. This analysis was conducted once. **f**, The final *N* + 10* oligonucleotide product was also assessed with MALDI-TOF and summarized along with any major impurities detected. Further data are given in Supplementary Table 2 regarding the observed and calculated *m*/*z* values for all oligonucleotide synthesis products and impurities generated by MALDI-TOF analysis, as well as their respective theoretical molecular weights. Obs., observed.

We next turned to performing 5× cycles of controlled, enzymatic synthesis to produce an *N* + 5* oligonucleotide with the natural RNA sequence *N* + U-U-U-C-G* in the liquid bulk phase using a Cy5-labeled initiator (Fig. 5a). To achieve longer synthesis lengths, we increased the initial scale to approximately 20 nmol in a volume of 8 ml and adjusted the extension and deblocking volumes accordingly after each cycle to maximize the efficiency of enzymatic coupling by maintaining standard reaction conditions (for example, 2.5 pmol µl^−1^ oligonucleotide). MALDI-TOF analysis after each cycle showed the successful formation of all extended and deblocked products, indicating a high coupling efficiency over the course of the enzymatic synthesis (Fig. 5b). This was confirmed with LC analysis, where we found excellent isolated purity of the oligonucleotide intermediates and final product (as measured by at 649 nm for Cy5) (Fig. 5c and Supplementary Fig. 7a,b).

Following a successful *N* + 5 synthesis, we next attempted to enzymatically synthesize an *N* + 10* oligonucleotide with the natural RNA sequence *N* + A-C-A-C-C-U-U-A-A-C* (Fig. 5d). Tracking the synthesis with high-resolution gel electrophoresis showed formation of all extension intermediates and the *N* + 10* final product (Fig. 5e), which had an isolated purity of 67% (as determined by LC at 649 nm) (Supplementary Fig. 8). MALDI-TOF analysis showed the expected mass of the *N* + 10* (9,738.8 *m*/*z*) in addition to a single *N* + 11* impurity (10,068.0 *m*/*z*) with an extra ‘A’ in the sequence (Fig. 5f). Possible explanations for this impurity include ATP carryover during later cycles of synthesis or the occurrence of a double-coupling event, as previously detected by LC analysis using our 3′-*O*-allyl ether ATP building block (Supplementary Fig. 4b). Another impurity with a mass of 8,305.30 m/z was found with MALDI-TOF; however, additional characterization is required to determine its exact composition.

### Incorporation of RT-NTPs with therapeutic modifications

**Fig. 6:**
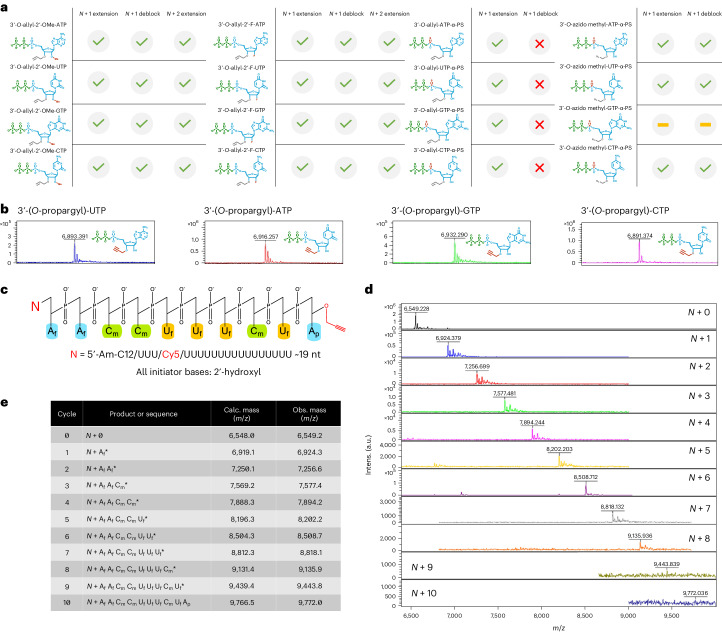
Compatibility summary of modified 3′-*O*-allyl ether and PS 3′-*O*-azido methyl ether RT-NTP sets and results of multicycle synthesis to produce a fully modified RNA oligonucleotide. **a**, Modified 3′-*O*-allyl ether and PS 3′-*O*-azido methyl ether RT-NTPs were evaluated by performing an initial *N* + 1* extension, a deblocking reaction and, if possible, an *N* + 2* extension. A green checkmark indicates a successful reaction, and a red cross-out indicates an unsuccessful reaction. Reactions that were not attempted are indicated by a yellow bar. Each individual cycle step was evaluated using MALDI-TOF mass spectrometry (Supplementary Figs. 11–13). **b**, MALDI-TOF assessment of enzymatic extension reactions using a set of 3′-*O*-propargyl ether NTPs (A, U, G, C) to install a functional handle onto oligonucleotides. **c**, A fully modified *N* + 10 oligonucleotide with the sequence *N* + A_f_-A_f_-C_m_-C_m_-U_f_-U_f_-C_m_-U_f_-A_p_ was synthesized using modified RT-NTPs, where f is 2′-fluoro, m is 2′-methoxy and p is 3′-*O*-propargyl. **d**, MALDI-TOF mass spectrometry was used to verify extension using the modified RT-NTPs during each cycle of enzymatic synthesis. **e**, The expected oligonucleotide sequences and their calculated and observed *m*/*z* values from MALDI-TOF analysis are summarized in the table. Further data are provided in Supplementary Table 2 regarding the observed and calculated *m*/*z* values for all oligonucleotide synthesis products and impurities generated by MALDI-TOF analysis, as well as their respective theoretical molecular weights. Calc., calculated.

Although the benefits of template-independent enzymatic synthesis of natural RNA oligonucleotides are numerous, all commercial RNA-based therapeutics are partially or fully modified^47^. We therefore accessed sets of modified 3′-*O*-allyl ether RT-NTP sets with either a 2′-F, 2′-OMe or alpha-phosphorothioate (α-PS) modification. We evaluated the capacity of each modified RT-NTP to control enzymatic synthesis by generating all single base transition (for example, A_m_ to A_m_, C_f_ to C_f_, where **f** is a 2′-F modification, **m** is a 2′-OMe) *N* + 2* extension products for each set (Fig. 6a). The formation of all expected oligonucleotide products with 2′-F and 2′-OMe modifications was observed with MALDI-TOF analysis using standard reaction conditions (Supplementary Figs. 9 and 10). Notably, we found that α-PS-modified 3′-*O*-allyl ether NTPs could be incorporated by our enzyme; however, deblocking with the allyl ether Pd/TPPTS chemistry resulted in formation of reduction side products, preventing us from obtaining the desired *N* + 2* product^48^ (Supplementary Fig. 11). To investigate an alternative reversible terminator chemistry that would be better suited for PS bonds, we accessed a partial set (A, U, C) of 3′-*O*-azido-methyl ether NTPs with the α-PS modification. Both enzymatic incorporation and deblocking, which was performed at room temperature using Tris (2-carboxyethyl) phosphine rather than Pd/TPPTS^49^, resulted in the desired *N* + 1* and *N* + 1 oligonucleotide products, respectively (Supplementary Fig. 21).

Strong and indiscriminate incorporation of all modified RT-NTPs by our enzyme was further exemplified by the generation of long homopolymer sequences in the presence of their unblocked counterparts (Supplementary Fig. 12). Similar results were found when we tested various propargyl-modified nucleotides with the intention of installing functional handles onto our oligonucleotide products. These handles provide a way to conjugate enzymatically synthesized oligonucleotides with important ligands such as GalNAc, which is commonly used to deliver therapeutic oligonucleotides to the liver^12,50^. Uncontrolled polymerization using unblocked *N*^6^-propargyl-ATP and 2-ethynyl-ATP resulted in generation of long homopolymer sequences (>100 nt), whereas a set of 3′-propargyl-ether-modified NTPs yielded *N* + 1 single extension products for each base (Fig. 6b and Supplementary Fig. 13). As a single terminal propargyl group was the preferred result of this activity, we labeled the functionalized *N* + 1 oligonucleotides with α-GalNAc-PEG3-azide using a standard click chemistry protocol. MALDI-TOF analysis indicated complete conjugation, marked by the total consumption of the unlabeled material (Supplementary Fig. 14). We did not label the homopolymer sequences generated by *N*^6^-propargyl-ATP and 2-ethynyl-ATP with the GalNAc ligand. However, our capacity to readily generate these sequences enables further exploratory opportunities in nucleic-acid-based materials^51^, as well as the modulation of messenger RNA stability with modifications to the polyA tail^52^.

### Multicycle synthesis of a modified RNA oligonucleotide

With a full palette of modified RT-NTPs at our disposal and having proved that our platform could accommodate nearly all of them, we set out to synthesize a fully modified RNA oligonucleotide of longer length as a final synthesis capstone. Starting with a 60 ml, 200 nmol liquid bulk phase reaction and using the standard Cy5-labeled initiator oligonucleotide, we performed 10× cycles of enzymatic synthesis to produce the sequence *N* + A_**f**_-A_**f**_-U_**m**_-U_**m**_-C_**f**_-C_**f**_-C_**f**_-U_**m**_-C_**f**_-A_**p**_, where **p** is a terminal 3′-propargyl (Fig. 6c). MALDI-TOF analysis, performed after each cycle, indicated the formation of all expected extension intermediates and final product (Fig. 6d,e). The overall yield of the synthesis was low, with ≤50 pmol of final product, which may have impeded observation of any arising impurities in the MALDI-TOF analysis during the last few cycles. Similarly, we were able to verify that the terminal propargyl group was enzymatically installed, but insufficient material made it challenging to label with a ligand such as α-GalNAc-PEG3-azide and to fully characterize the final product. Nonetheless, this capstone represents, to our knowledge, the first controlled synthesis of a fully modified oligonucleotide with RT-NTPs and a template-independent polymerase.

### Initiator oligonucleotide cleavage with endonuclease V

After successful synthesis of a fully modified RNA oligonucleotide with our platform, we considered it crucial to develop a method to remove the undesired initiator sequence from the final product. To do this, we exploited the ability of the endonuclease V enzyme from *Escherichia coli* to cleave single-stranded oligonucleotides in a site-specific manner^53^. Endonuclease V cleavage is mediated by recognition of an inosine placed rationally within the initiator sequence^54^. Cleavage occurs two bases downstream of the inosine, and products are left with a 5′- phosphate group, which can be removed with a phosphatase (Supplementary Figs. 15 and 16). An endonuclease V cleavage site can be either preinstalled during initiator production or added by enzymatic incorporation of an inosine reversible terminator nucleotide. To demonstrate this, we accessed a 3′-*O*-allyl ether inosine building block and validated its general functionality as a reversible terminator (Supplementary Fig. 17a). We then performed uncontrolled polymerization of an *N* + I deblocked oligonucleotide product to generate a long homopolymer and verified that the initiator sequence, which featured a 5′-Cy5 modification, could be cleaved from the homopolymer product (Supplementary Fig. 17b). The desired oligonucleotide product could then be isolated from the initiator sequence through further enrichment or purification.

### Evaluation of CPG support system for solid phase synthesis

**Fig. 7:**
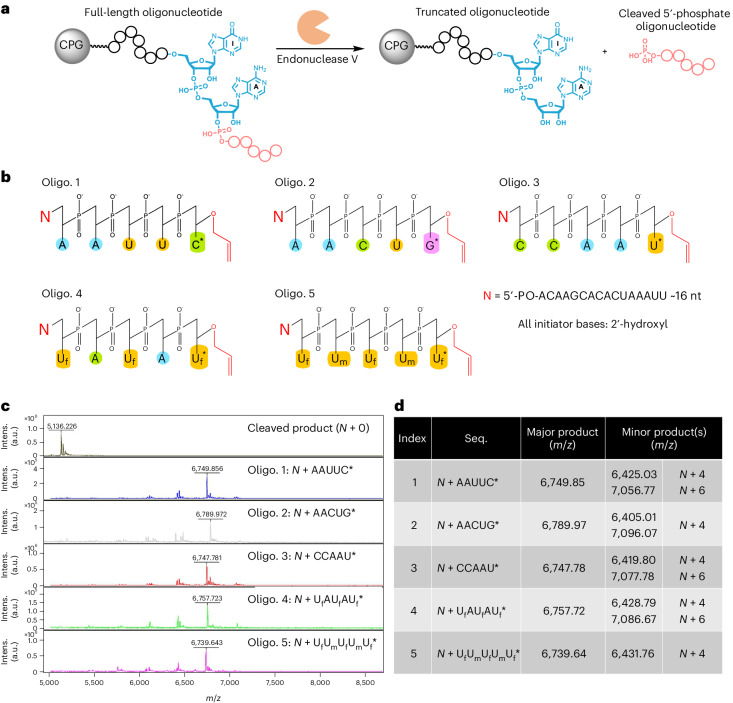
Overview and demonstration of a solid support system for controlled enzymatic RNA oligonucleotide synthesis. **a**, Outlined of a general scheme: an initiator oligonucleotide (black) is bound to LCAA-CPG with a Bis(NHS)PEG5 linker (pink) using NHS conjugation chemistry. The initiator harbors a deoxy- or riboinosine base (red) for recognition by *E. coli* endonuclease V, which cleaves the desired oligonucleotide product (blue) immediately downstream of the inosine base. The oligonucleotide product can then be isolated from the CPG solid support. **b**, To demonstrate the viability of the CPG solid support, 5× *N* + 5 oligonucleotides were enzymatically synthesized in a stir reactor format. Their sequences comprised natural and modified bases, with one partially modified with 2′-fluoro groups and another fully modified with both 2′-fluoro and 2′-methoxy groups. **c**, MALDI-TOF mass spectrometry was used to evaluate the oligonucleotide material cleaved from the solid support. **d**, Summary of enzymatic synthesis, including a high-level description of the major and minor products found. Further data are provided in Supplementary Table 3 regarding the observed and calculated *m*/*z* values for all oligonucleotide synthesis products and impurities generated by MALDI-TOF analysis, as well as their respective theoretical molecular weights. Seq., sequence; oligo., oligonucleotide.

In addition to providing a method to remove the initiator sequence from the enzymatically synthesized oligonucleotide product, endonuclease V-mediated cleavage formed a basis for the development of a solid support system for our platform (Fig. 7a). A robust and economical solid support should enable the synthesis of longer oligonucleotides at commercially relevant scales, in comparison with synthesis in the liquid bulk phase. To access such a solid support, we added a Bis(NHS)PEG5 linker to a long-chain alkylamine controlled-pore glass (LCAA-CPG) and then labeled it with a 5′-amine initiator oligonucleotide using NHS conjugation chemistry. We used an initiator oligonucleotide that had a preinstalled inosine and demonstrated that incubating labeled CPG in a stir reactor format with endonuclease V cleaved the initiator at the desired location, releasing the oligonucleotide downstream of the inosine (Supplementary Fig. 18). We next tested a single cycle of controlled extension and deblocking on the CPG solid support using each 3′-*O*-allyl ether NTP and found successful formation of the desired *N* + 1 products in all cases after endonuclease V cleavage (Supplementary Fig. 19).

Building on these experiments, we performed controlled enzymatic synthesis of 5× unique *N* + 5* oligonucleotides with unmodified, partially modified and fully modified sequences on our solid support (Fig. 7b). For these syntheses, the inosine base was preinstalled in the surface-bound initiator oligonucleotide. In general, it was observed that all five RNA oligonucleotides were successfully synthesized upon cleavage from the solid support. In MALDI-TOF analysis, the major peaks corresponded to the expected masses of the intended sequences (Fig. 7c). The primary impurities were *N* + 4 and *N* + 6 products, which may have arisen from incomplete access of the enzyme to surface-bound oligonucleotide or carryover of NTPs from inadequate washing of the solid support (Fig. 7d). To better address and prevent such impurities, future work should include packing the CPG solid support into a closed column with precise flow control.

## Discussion

In this work, we report the development and optimization of a platform for controlled, template-independent enzymatic RNA oligonucleotide synthesis. Using aqueous-based reaction conditions, we produced several natural and modified RNA oligonucleotide sequences using 3′-*O*-allyl ether RT-NTPs and mutant variants of *S. pombe* CID1 PUP in the liquid bulk phase. We found that phosphorothioate backbone modifications were not tolerated by the allyl ether deblocking chemistry and showed that the 3′-*O*-azido-methyl ether blocking group may be a viable alternative. Removal of the initiator oligonucleotide from the final product was made possible by endonuclease V cleavage following recognition of a preinstalled inosine base. This functionality served as the basis for the development of a CPG solid support system, which we showed is also capable of multicycle enzymatic synthesis of both natural and modified sequences.

In general, we found that our enzymatic platform was comparable with chemical methods in terms of building block coupling efficiencies and cycle times; however, synthesis yields were lower than those expected for traditional phosphoramidite chemistry^55^. These low synthesis yields were probably the result of multiple rounds of purification after each extension and deblocking reaction in the liquid bulk phase. Moving to a solid support system, such as the one described in this work, will be critical to retaining more oligonucleotide product after each cycle of enzymatic synthesis. Yield parity with chemical synthesis and scaling of our process may be further achieved by packing the loose solid support into a column-based format. In this case, the maximum size and volume of the column may not be limited by logistical considerations such as solvent storage and handling as well as the disposal of hazardous waste^19^. Other optimizations such as increasing initiator oligonucleotide loading density on the solid support will be important for scale, batch throughput and, ultimately, the cost of goods.

Although only a few enzymatic oligonucleotide synthesis technologies have emerged thus far^20,56^, it is clear that they have many advantages over chemical methods. Not only will aqueous synthesis reduce the overall environmental impact, it could also bolster the ability to manufacture RNA oligonucleotide therapeutics with better atom economies, reduced process mass intensities, and less rigorous downstream purifications^19,20^. In addition, enzymatic synthesis may address the length limitations commonly associated with phosphoramidite chemistry, as an enzymatic process has the potential for higher fidelity and fewer side reactions^57^. This will be important for accessing high-quality guide RNAs for CRISPR–Cas gene editing applications, as these are typically greater than 80-nt in length^58^. Enzymatic RNA synthesis is in the early stages of development, and more optimization is required to surpass chemical synthesis standards; however, it shows immense potential to deliver on the promise of RNA oligonucleotide therapeutics in a sustainable way.

## Methods

### Preparation of 3′-*O*-allyl ether NTPs (A, U, G, C)

A mixture of 3′-*O*-allyl ether adenosine nucleoside (molecular weight: 307.3 g mol^−1^, 1.04 g, 3.38 mmol) and proton sponge (molecular weight: 214.31 g mol^−^^1^, 2.03 g, 9.47 mmol) was prepared as described in the general procedure, dissolved in trimethoxy phosphate (25.0 ml) and cooled to −5 °C. This was followed by slow addition of phosphoryl oxychloride (molecular weight: 153.32 g mol^−1^, density: 1.64 g cm^−^^3^, 0.35 ml, 3.7 mmol). After 3 min, another portion of phosphoryl oxychloride (molecular weight: 153.32 g mol^−1^, density: 1.64 g cm^−^^3^, 0.1 ml, 1.1 mmol) was added. After stirring for 10 min, a prechilled mixture of tributyl ammonium pyrophosphate (molecular weight: 548.68 g mol^−1^, 7.0 g, 12.8 mmol), acetonitrile (55 ml) and tributyl amine (12 ml) was quickly added to the reaction. This was stirred for 2 h and slowly warmed to room temperature. The reaction was quenched by the addition of water (~150 ml) and worked up, isolated and purified according to the general procedure (yield 33%, formula weight for the tetra-triethylammonium salt: 951.5 g mol^−1^, 1.65 g, 1.11 mmol). ^1^H NMR (400 MHz, D_2_O) δ 8.43 (s, 1H), 8.15 (s, 1H), 6.04 (d, *J* = 6 Hz, 1H), 5.96 (m, *J* = 17, 11 Hz, 1H), 5.37 (dd, *J* = 17, 2 Hz, 1H), 5.23 (dd, *J* = 11, 2 Hz, 1H), 4.80 (t, *J* = 6 Hz, 1H), 4.42 (t, *J* = 3 Hz, 1H), 4.30 (dd, *J* = 6, 3 Hz, 1H), 4.19 (d, *J* = 6 Hz, 2H), 4.15 (t, *J* = 3 Hz, 2H), 3.09 (q, 16 H), 1.18 (t, 24H). ^31^P NMR (400 MHz, D_2_O) δ −10.6 (d, *J* = 20, 1H), −11.5 (d, *J* = 20, 1H), −23.1 (t, *J* = 20). ESI-MS: calculated for [C_13_H_21_N_5_O_13_P_3_]^+^ = 548.0343; found: 548.0347.

A mixture of 3′-*O*-allyl ether uridine nucleoside (molecular weight: 284.10 g mol^−1^, 0.57 mg, 2.00 mmol) and proton sponge (molecular weight: 214.31 g mol^−1^, 1.00 g, 4.67 mmol) was prepared as described in the general procedure, dissolved in trimethoxy phosphate (9.0 ml) and cooled to −5 °C. This was followed by slow addition of phosphoryl oxychloride (molecular weight: 153.32 g mol^−1^, density: 1.64 g cm^−^^3^, 0.2 ml, 2.1 mmol). After 3 min, another portion of phosphoryl oxychloride (molecular weight: 153.32 g mol^−1^, density: 1.64 g cm^−^^3^, 0.1 ml, 1.1 mmol) was added. After stirring for 10 min, a prechilled mixture of tributyl ammonium pyrophosphate (molecular weight: 548.68 g mol^−1^, 3.7 g, 6.7 mmol), acetonitrile (32 ml) and tributyl amine (6 ml) was quickly added to the reaction. This was stirred for 2 h and slowly warmed to room temperature. The reaction was quenched by the addition of water (~200 ml) and worked up, isolated and purified according to the general procedure (yield 29%, formula weight for the tetra-triethylammonium salt: 928.5 g mol^−1^, 0.54 g, 0.58 mmol). ^1^H NMR (400 MHz, D_2_O) δ 7.88 (d, *J* = 8 Hz, 1H), 5.93 (m, 3 H), 5.32 (dd, *J* = 17, 2 Hz, 1H), 5.22 (dd, *J* = 11, 2 Hz, 1H), 4.40 (t, *J* = 6 Hz, 1H), 4.32 (m, *J* = 3 Hz, 1H), 4.15 (m, *J* = 3 Hz, 3H), 3.12 (q, 19 H), 1.20 (t, 29 H). ^31^P NMR (400 MHz, D_2_O) δ −10.8 (d, *J* = 20, 1H), −11.6 (d, *J* = 20, 1H), −23.3 (t, *J* = 20, 1H). ESI-MS: calculated for [C_12_H_18_N_2_O_15_P_3_]^−^ = 522.9926; found: 522.9928.

A mixture of 3′-*O*-allyl ether guanine nucleoside (molecular weight: 323.12 g mol^−1^, 1.1 g, 3.40 mmol) and proton sponge (molecular weight: 214.31 g mol^−1^, 2.21 g, 10.3 mmol) was prepared as described in the general procedure, dissolved in trimethoxy phosphate (25.0 ml) and cooled to −5 °C. This was followed by slow addition of phosphoryl oxychloride (molecular weight: 153.32 g mol^−1^, density: 1.64 g cm^−^^3^, 0.35 ml, 3.7 mmol). After 3 min, another portion of phosphoryl oxychloride (molecular weight: 153.32 g mol^−1^, density: 1.64 g cm^−^^3^, 0.1 ml, 1.1 mmol) was added. After stirring for 10 min, a prechilled mixture of tributyl ammonium pyrophosphate (molecular weight: 548.68 g mol^−1^, 7.0 g, 12.8 mmol), acetonitrile (55 ml) and tributyl amine (12 ml) and was quickly added to the reaction. This was stirred for 2 h and slowly warmed to room temperature. The reaction was quenched by the addition of water (~200 ml) and worked up, isolated and purified according to the general procedure (yield 40%, formula weight for the tetra-triethylammonium salt: 967.5 g mol^−1^, 1.33 g, 1.37 mmol). ^1^H NMR (400 MHz, D_2_O) δ 8.03 (s, 1H), 5.96 (ddt, *J* = 6 Hz, 1H), 5.84 (d, *J* = 7 Hz, 1H), 5.35 (dd, *J* = 17 Hz, 1H), 5.23 (dd, *J* = 11 Hz, 1H), 4.84 (dd, *J* = 7 Hz, 1H), 4.39 (m, *J* = 3 Hz, 1H), 4.29 (dd, *J* = 5, 3 Hz, 1H), 4.19 (d, *J* = 6 Hz, 2H), 4.15 (m, *J* = 6 Hz, 2H), 3.11 (q, 19H), 1.19 (t, 29H). ^31^P NMR (400 MHz, D_2_O) δ −10.6 (d, *J* = 20 Hz, 1H), −11.5 (d, *J* = 20 Hz, 1H), −23.1 (t, *J* = 20 Hz, 1H). ESI-MS: calculated for [C_13_H_19_N_5_O_14_P_3_]^−^ = 562.0147; found: 562.0150.

The 3′-*O*-allyl ether cytidine nucleoside (molecular weight: 283.3 g mol^−^^1^, 0.22 g, 0.78 mmol) was dissolved in a mixture of dimethylformamide (DMF) and 1,1-dimethoxytrimethylamine (~6:1, 2.1 ml) and stirred for 36 h. The crude product was concentrated in vacuo. To the resultant solid was added dry proton sponge (molecular weight: 214.3 g mol^−1^, 10.45 g, 2.1 mmol), and the mixture was dried on a lyophilizer overnight in the reaction flask. Under a blanket of argon, the mixture was dissolved in trimethoxy phosphate (5.0 ml). This solution was cooled to −5 °C, and phosphoryl oxychloride was added (0.07 ml, 0.75 mmol); after 3 min, another portion of phosphoryl oxychloride (0.03 ml, 0.32 mmol) was added. This solution was stirred in a cold bath for about 20 min. After this time, prechilled tributyl ammonium pyrophosphate (1.4 g, 2.6 mmol) and tributyl amine (2.4 ml) in acetonitrile (11 ml) were added in one portion. This was stirred for 2 h and slowly warmed to room temperature. The reaction was quenched by the addition of water and worked up, isolated and purified according to the general procedure (yield 39%, formula weight for the tetra-triethylammonium salt: 927.5 g mol^−^^1^, 283.0 mg, 0.31 mmol). ^1^H NMR (400 MHz, D_2_O) δ 7.90 (d, *J* = 8 Hz, 1H), 6.09 (d, *J* = 8 Hz, 1H), 5.93 (m, *J* = 6, 5 Hz, 2H), 5.32 (dd, *J* = 17, 2 Hz, 1H), 5.22 (dd, *J* = 10, 2 Hz, 1H), 4.36 (t, *J* = 5 Hz, 1H), 4.30 (dt, *J* = 3 Hz, 1H), 4.22 (ddd, *J* = 3 Hz, 1H), 4.14 (m, 2H), 3.13 (q, 11H), 1.21 (t, 18H). ^31^P NMR (400 MHz, D_2_O) δ −10.42 (d, *J* = 20 Hz, 1H), −11.47 (d, *J* = 20 Hz, 1H), −23.12 (t, *J* = 20 Hz, 1H). ESI-MS: calculated for [C_12_H_19_N_3_O_14_P_3_]^−^ = 522.0085; found: 522.0089.

### PUP expression and purification

The DNA sequences for the wild-type CID1 *S. pombe* PUP (SEQ1) and mutant variants (H336R (SEQ2) and H336R-N171A-T172S (SEQ3)) were codon optimized for expression in *E. coli*, ordered as gBlocks (IDT) fragments and inserted into the pET-28-a-(+) expression vector (EMD Millipore 69864-3) using 2X Gibson Assembly Master Mix (NEB E2611) per the manufacturer’s instructions. High-efficiency T7 Express chemically competent *E. coli* cells (NEB C2566) were transformed with the fully assembled plasmid per the manufacturer’s instructions, and positive transformants were selected for on LB-kanamycin plates. Several bacterial colonies were picked, and sent for Sanger sequencing (Azenta) using the T7 forward and T7-Term primers. Those with correct sequences were grown in liquid LB-kanamycin media (Fisher 10-855-021) overnight at 37 °C, diluted the next morning (1:400) in fresh liquid LB supplemented with 50 µg ml^−1^ kanamycin (Sigma K1377) and induced with high-grade isopropyl β-d-1-thiogalactopyranoside (Sigma I5502) at an optical density at 600 nm of 0.6. The induced liquid cultures were incubated overnight at 15 °C, with shaking at 250 rpm. Cultures were then pelleted at 3,500*g* for 10 min and His-Tag purified using HisTalon Metal Affinity Resin per the manufacturer’s instructions (Takara 635503, 635623 and 635651). The eluted enzyme samples were concentrated and buffer-exchanged into 1× PUP storage buffer (10 mM Tris-HCl (Thermo AM9855G), 250 mM NaCl (Thermo AM9760G), 1 mM DTT (Sigma D9779), 0.1 mM EDTA (Thermo AM9260G), pH 7.5 at 25 °C) using Amicon 30K MWCO 15 ml filter columns (Sigma UFC9030), flash frozen using liquid nitrogen and stored at −80 °C until needed.

### Endonuclease V expression and purification

Wild-type *E. coli* endonuclease V (SEQ4) and endonuclease V fused to a maltose binding protein at the amino terminus (SEQ 5) were expressed and purified as described for PUP, with the exception of the 1× Endo V storage buffer being composed of 10 mM Tris-HCl, 250 mM NaCl, 0.1 mM EDTA and 1 mM DTT, pH 8.0, at 25 °C. Expressed enzyme was flash frozen using liquid nitrogen and stored at −80 °C until needed.

### Standard liquid bulk phase reactions

#### Controlled enzymatic extension reactions with PUP

A standard master mix for controlled oligonucleotide enzymatic extension in the bulk liquid phase was composed of 1× extension buffer (50 mM NaCl, 10 mM Tris-HCl, 8 mM MgCl_2_ (Thermo AM9530G), 2 mM MnCl_2_ (RPI M20100) and 1 mM DTT at pH 7.9), 0.1 mg ml^−1^ purified enzyme, 1 mM 3′-*O*-allyl ether RT-NTP and 2.5 pmol µl^−1^ initiator oligo. All extension reactions were carried out at 37 °C for 30 min unless otherwise specified. Following incubation, 2 µl proteinase K (NEB P8107) was added to the samples, followed by gentle mixing and incubation for 5 min at 37 °C. Extension products were then isolated and purified using Oligonucleotide Clean and Concentrator spin-columns (Zymo D4060) following the manufacturer’s instructions and eluted in MilliQ water. All standard liquid bulk phase extension reactions used an internally Cy5-labeled, 19-nt RNA initiator oligo comprised of the sequence 5-AmMC12/-rU-rU-rU-/iCy5/-rU-rU-rU-rU-rU-rU-rU-rU-rU-rU-rU-rU-rU-rU-rU-rU (IDT) and PUP mutant variant H336R unless otherwise specified.

#### Allyl ether deblocking reactions

A standard allyl ether deblocking reaction consisted of degassed 10 mM Tris-HCl (pH 6.7), 1.15 nmol µl^−1^ sodium tetrachloropalladate(II) (Na_2_PdCl_4_) (Sigma 205818), 8.80 nmol µl^−1^ triphenylphosphine-3,3′,3′′-trisulfonic acid trisodium salt (P(PhSO_3_Na)_3_) (Sigma 744034) and 2.5 pmol µl^−1^ blocked RNA oligonucleotide in MilliQ water. All deblocking reactions were carried out at 62 °C for 12 min. Deblocked oligonucleotide was then purified using Oligonucleotide Clean and Concentrator spin-columns (Zymo) and eluted in MilliQ water.

#### Azido methyl ether deblocking reactions

A standard azido methyl ether deblocking reaction was composed of degassed 10 mM Tris-HCl, 0.25 M Tris(2-carboxyethyl)phosphine hydrochloride (Sigma C4706) and 2.5 pmol µl^−1^ blocked RNA oligonucleotide in MilliQ water. Deblocking reactions were carried out at room temperature (~20 °C) for approximately 5 min. Deblocked oligonucleotide was then purified using Oligonucleotide Clean and Concentrator spin-columns (Zymo) and eluted in MilliQ water.

#### Endonuclease V-mediated oligonucleotide cleavage reactions

A standard endonuclease V-mediated cleavage reaction in the liquid bulk phase was carried out by incubating 2.5 pmol µl^−1^ initiator oligonucleotide containing a deoxy- or riboinosine base in 1× cleavage buffer (50 mM potassium acetate (Thermo J60832.AK), 20 mM Tris-acetate (Bioworld 42020180), 10 mM magnesium acetate (Thermo J60041.AE), 1 mM DTT, pH 7.9 at 25 °C) and 0.05 mg ml^−1^ endonuclease V at 37 °C for 30 min. For commercially sourced endonuclease V (NEB M0305), 20 U enzyme was added to reactions. Cleaved oligonucleotide was purified using Oligonucleotide Clean and Concentrator spin-columns (Zymo) and eluted into MilliQ water for downstream analysis. Wild-type endonuclease V prepared in-house was used for all standard cleavage reactions unless otherwise noted.

#### Phosphatase-mediated oligonucleotide dephosphorylation reactions

A standard dephosphorylation reaction in the liquid bulk phase was carried out by incubating an 5′-phosphate modified oligonucleotide with 100 U Antarctic phosphatase (NEB M0289) in 1× NEB 4 (50 mM bis-Tris-propane-HCl, 1 mM MgCl_2_ and 0.1 mM ZnCl_2_, pH 6 at 25 °C) at a concentration of 2.5 pmol µl^−1^ for 30 min at 37 °C. Dephosphorylated oligonucleotide was purified using Oligonucleotide Clean and Concentrator spin-columns (Zymo) and eluted in MilliQ water.

#### Preparation of CPG solid support derivatized with initiator oligonucleotide

The initiator-oligonucleotide-labeled solid support was prepared by covalently attaching a 5′-amine-modified oligonucleotide to the surface of LCAA-CPG using a bis-*N*-hydroxysuccinimide ester linker. Then, 2 g of dry LCAA-CPG with a pore size of 1,000 Å (ChemGenes N-5100-10) was added to a 20 ml scintillation vial and washed three times with 15 ml anhydrous DMF (Sigma 227056). The vial containing LCAA-CPG was rotated for 15 min during each DMF wash, and liquid waste was discarded. A 100 mg ml^−1^ solution of bis-PEG5-NHS ester linker (BroadPharm BP-20429) was prepared in anhydrous DMF. After the final wash of the LCAA-CPG, 5 ml of the 100 mg ml^−1^ bis-PEG5-NHS ester linker solution was added. Additional anhydrous DMF was added (~4–5 ml) to bring the solution to volume, and then the vial was incubated at room temperature for 2 h with rotation. After incubation, the bis-PEG5-NHS solution was discarded, and the LCAA-CPG was washed three times with anhydrous DMF. To link the initiator oligonucleotide to the now-derivatized LCAA-CPG, 500 µl of a 1 mM solution containing the 5′-amine-modified oligonucleotide (sequence: 5′-NH_2_-C12-rU-rC-rU-rA-rC-rC-rA-rU-rA-rU-rA-rU-dI-rA-rA-rC-rA-rA-rG-rC-rA-rC-rA-rCr-U-rA-rA-rA-rU-rU) (IDT), where dI is deoxyinosine) was prepared in MilliQ water and directly added to the LCAA-CPG in the vial, along with an additional 10 ml of fresh anhydrous DMF. This solution was incubated at room temperature for at least 4 h with rotation. After incubation, the initiator-oligonucleotide-labeled CPG solid support was washed three times with anhydrous DMF and then with a 0.1 M solution of succinimide anhydride (Sigma 239690) to cap any remaining primary amine sites on the surface of the LCAA-CPG. The solid support was then transferred to a 20 ml solid phase extraction (SPE) column with filter and washed in excess with a 10 mM Tris-HCl solution using a vacuum manifold. The resultant labeled CPG solid support was then stored at 4 °C until needed for enzymatic RNA oligonucleotide synthesis.

### Standard solid phase reactions

#### Controlled enzymatic extension reactions on CPG solid support

A standard solid phase enzymatic extension reaction was conducted by incubating 150 mg of initiator-oligonucleotide-labeled CPG solid support with 1× extension buffer (50 mM NaCl, 10 mM Tris-HCl, 8 mM MgCl_2_, 2 mM MnCl_2_, 1 mM DTT, at pH 7.9), 0.1 mg ml^−1^ enzyme and 1 mM allyl ether RT-NTP terminator in a total volume of 1.5 ml. Reactions were carried out in a ‘stir format’, in which the CPG solid support and extension reaction master mix were combined in a capped 3 ml SPE column containing a small flea-sized magnetic stir bar and placed on a custom-made heat block/magnetic stir plate set to 37 °C and 1,500 rpm, respectively, for 30 min (Supplementary Fig. 18). Following incubation, the SPE column was uncapped and placed on a vacuum manifold, where the extension master mix was discarded. The solid support was then washed two times with 3 ml of DNA wash buffer (Zymo D4003) and five times with 3 ml of 10 mM Tris-HCl (pH 6.7). During each wash, the CPG solid support was gently agitated with a 1 ml pipette to ensure complete washing. The SPE column was then removed from the vacuum manifold, capped and placed on ice or stored at 4 °C until needed.

#### Allyl ether deblocking reactions on CPG solid support

To remove the 3′-*O*-allyl ether blocking group from the growing oligonucleotide on the surface of the CPG solid support, 1 ml of deblocking solution (degassed, 10 mM Tris-HCl (pH 6.7), 1.15 nmol µl^−1^ Na_2_PdCl_4_ and 8.80 nmol µl^−1^ P(PhSO_3_Na)_3_) was prepared and added directly to the SPE column. The SPE column was then placed on the combination heat block/magnetic stir plate and incubated at 62 °C for 12 min without stirring. After incubation, the SPE column was placed on a vacuum manifold, where the deblocking solution was immediately discarded. The solid support was then washed once with 3 ml of 3% ammonium hydroxide (Sigma 05002), two times with 3 ml of DNA wash buffer and five times with 3 ml of a 10 mM Tris-HCl solution (pH 6.7). During each wash, the CPG solid support was gently agitated with a 1 ml pipette to ensure complete washing. The SPE column was then removed from the vacuum manifold, capped and placed on ice until the subsequent enzymatic extension step or cleavage from the surface.

#### Enzymatic cleavage from the CPG solid support

Once enzymatic RNA synthesis had been completed, the oligonucleotide product was cleaved and collected by incubating 150 mg of CPG solid support with 1× cleavage buffer (50 mM potassium acetate, 20 mM Tris-acetate, 10 mM magnesium acetate, 1 mM DTT, pH 7.9 at 25 °C) and 0.05 mg ml^−1^ endonuclease V at 37 °C for 30 min in a total volume of 0.750 ml. Cleavage reactions were carried out as before in a ‘stir format’ (Supplementary Fig. 18), in which the same SPE column containing the CPG solid support and magnetic flea was incubated at 37 °C and spun at 1,500 rpm for 30 min. After incubation, the cleaved oligonucleotide was collected by placing the uncapped SPE column in a 15 ml empty falcon tube and centrifuging for 1 min at 1,000*g*. The RNA oligonucleotide product was then stored at −20 °C until needed for analysis or downstream applications.

### Conjugation of GalNAc ligand to propargyl functional handles using click chemistry

The following stock solutions were prepared before the click chemistry protocol was performed: 5 mM ascorbic acid (Sigma A92902) in MilliQ water, 10 mM copper (II)-TBTA (Tris(benzyltriazolylmethyl)amine) in 55% dimethyl sulfoxide (DMSO); prepared by dissolving 25 mg copper (II) sulfate pentahydrate (Sigma 209198) in 10 ml MilliQ water and mixing with a solution of 58 mg of TBTA ligand (Sigma 678937) in 11 ml of anhydrous DMSO) and 2 M triethylammonium acetate buffer, pH 7.0 (prepared by mixing 2.78 ml triethylamine (TEA, Chem-Impex 00319) with 1.14 ml of glacial acetic acid (Fisher A38-500), bringing the volume to 10 ml and adjusting the pH to 7.0). A stock solution of α-GalNAc-PEG3-azide ligand (Sigma SMB00392) was prepared at a final concentration of 10 mM in 100% anhydrous DMSO. Click chemistry reactions took place in a 1.5 ml high-performance LC (HPLC) glass vial with the following standard components: 200 mM triethylammonium acetate buffer, 0.5 mM ascorbic acid, 0.5 mM copper (II)-TBTA complex, 30 µM α-GalNAc-PEG3-azide and 20 µM 3′-*O*-propargyl-ether-modified RNA oligonucleotide (previously dissolved in MilliQ water) in a total volume of 100 µl. A low flow of high-purity argon was bubbled through the click reaction for 30 s, and then the HPLC vial was sealed tightly. Reactions were carried out overnight for 12 h at room temperature, and the α-GalNAc-PEG3-labeled RNA oligonucleotides were purified using Oligonucleotide Clean and Concentrator spin-columns (Zymo) and eluted in MilliQ water for downstream analysis.

### Analysis of RNA oligonucleotide product mass, purity and concentration

Enzymatic RNA oligonucleotide synthesis product profiles were analyzed by a combination of high-resolution gel electrophoresis, MALDI-TOF mass spectrometry and LC/MS. A NanoDrop spectrophotometer (Thermo) was used to determine the concentrations of all oligonucleotide products based on absorbance at 260 nm. In instances where an oligonucleotide initiator, intermediate or final product featured an internal Cy5 dye, the absorbance at 649 nm was used to directly assess its crude purity in the presence of impurities that absorbed at 260 nm (which were generally buffer components and additives, such as guanidinium chloride, used for isolation of oligonucleotide from bulk liquid phase reactions).

#### High-resolution gel electrophoresis

For high-resolution gel electrophoresis, 15% TBE-urea denaturing gels (Thermo EC68855) were loaded with approximately 10–100 pmol of oligonucleotide material and run for 90 min at 185 V per the manufacturer’s instructions. If necessary, gels were then incubated with 1X GelStar nucleic acid stain (Lonza 50535) for 10 min on an orbital shaker. Gels were imaged with a Azure Sapphire Biomolecular Imager using the appropriate laser and filter settings (SYBR: 497 nm | 520 nm; Cy5: 651 nm | 670 nm).

#### MALDI-TOF mass spectrometry

Oligonucleotide masses were analyzed using MALDI-TOF by mixing 0.5 µl prepared MALDI matrix (50 mg ml^−1^ 3-hydroxypicolinic acid (Sigma 56197) and 10 mg ml^−1^ ammonium citrate (Sigma 247561) in a solution of 50/50 MS-grade acetonitrile (Sigma 900667) and MilliQ water) with 0.75 µl purified oligonucleotide directly on a 384-spot polished steel target plate. Samples were dried under vacuum for 5 min before analysis on a Bruker autoflex MALDI-TOF using flexControl software (v.3.4). Peak acquisition was performed in positive polarity mode using an in-source decay with reflector engaged method. Analysis of acquired data was performed using Bruker flexAnalysis software (v.3.4), with all peaks transformed and smoothed using the built-in baseline subtraction feature.

#### LC/MS analysis

The final mass and purity of oligonucleotide intermediates and final products were assessed with an Agilent 1200 series LC system with diode array detection and XBridge Oligonucleotide BEH C18 column (130 Å, 2.5 µm, 4.6 mm × 50 mm) (Waters 186003953) using a reversed phase method (mobile phase A: 5:95 methanol/water, 400 mM 1,1,1,3,3,3-hexafluoro-2-propanol (HFIP) (Chem-Impex 00080), 15 mM TEA (Chem-Impex 00319); mobile phase B: 50:50 methanol/water, 400 mM HFIP, 15 mM TEA; method: 56% isocratic over 60 min). Mass spectra were obtained by running an Agilent 6400 series single-quadrupole MS module in scanning negative mode. Deconvolution was performed using the Agilent Bioanalysis software package.

### Preparation of nucleoside triphosphate building blocks from nucleoside intermediates

#### Procurement and preparation of reaction components

All natural (2′-OH) and 2′-modified (-F, -OMe) nucleoside intermediates were purchased from ChemGenes Corporation with the 3′-*O*-allyl ether blocking group preinstalled as a custom order. Installation of the α-PS during triphosphorylation was outsourced as a custom order to Jena Bioscience using the nucleoside intermediates purchased from ChemGenes. The α-PS-modified 3′-*O*-azido-methyl ether RT-NTPs were accessed similarly, with ChemGenes providing nucleosides and Jena Bioscience performing triphosphorylation. Propargyl-modified nucleotides including 3′-*O*-propargyl-A, U, G, C, as well as *N*^6^-propargyl-ATP and 2-ethynyl-ATP, were purchased from Jena Bioscience (catalog numbers NU-945, NU-946, NU-947, NU-948, CLK-NU-001 and CLK-NU-004). Our method for the synthesis of nucleoside triphosphates from their nucleoside intermediates followed that previously reported in the literature^59^. Cytidine nucleosides were base transformed to their *N*^4^-DMF-C protected versions before triphosphorylation (Fig. 3b). Triphosphorylation reactions were carried out in dried glassware, under an argon atmosphere, using anhydrous acetonitrile (Sigma 271004) and tributyl amine (Sigma 90781). In addition, for triphosphorylation reactions, the nucleoside and proton sponge were premixed in their reaction flasks and vacuum dried overnight. Nucleosides that would not dissolve readily were gently heated until they were almost completely dispersed in the solution.

#### Standard triphosphorylation conditions

Several specific triphosphorylation reactions are discussed in detail in the ‘Preparation of 3′-*O*-allyl ether NTPs (A, U, G, C)’ section. All triphosphorylation reactions followed this general procedure: a mixture of nucleoside (3.38 mmol, 1 eq.) and proton sponge (9.47 mmol, 2.8 eq.) was prepared as described in the general procedure above. This mixture was dissolved in trimethoxy phosphate (25.0 ml) and cooled to −5 °C, followed by slow addition of phosphoryl oxychloride (3.7 mmol, 1.1 eq.). After 3 min, another portion of phosphoryl oxychloride (1.1 mmol, 0.3 eq.) was added. After stirring for 10 min, a prechilled mixture of tributyl ammonium pyrophosphate (12.8 mmol, 3.8 eq.), acetonitrile (55 ml) and tributyl amine (12 ml) was quickly added to the reaction. This was stirred for 2 h and then warmed to room temperature. The reaction was quenched by the addition of water (~150 ml) and worked up, isolated and purified according to the general procedure defined in the section entitled ‘Isolation and purification of prepared nucleoside triphosphates’.

#### Isolation and purification of prepared nucleoside triphosphates

Isolation and purification of nucleoside triphosphates was optimized and generally carried out as follows. Crude, quenched reaction mixture was washed with dichloromethane. The aqueous layer containing triphosphorylated product was washed with hexane and concentrated in vacuo. The isolated material was purified via ion-exchange chromatography (DEAE Sepharose resin; mobile phase A: MilliQ water; mobile phase B: 1 M triethyl ammonium bicarbonate buffer, pH 8 ± 0.5). The fractions containing primarily triphosphate (assayed via LC-MS) were combined and concentrated in vacuo. The sample was then further purified via preparatory HPLC (1260 Infinity Preparative LC System and Phenomenex Jupiter C18 reverse-phase column; 10 µm particle size, 300 Å pore size, 250 mm length, 21.2 mm diameter). Generally, a single method provided excellent purities for the various NTP products (mobile phase A: 0.1% ammonium acetate in acetonitrile; mobile phase B: 0.1% ammonium acetate in 1:667 water/acetonitrile; general method: 5% isocratic over 20 min, then to 90% over 30 min). Pure fractions were combined, frozen and lyophilized. The counter-ions on the triphosphate were exchanged by diluting the lyophilized sample in triethyl ammonium bicarbonate (1 M) and concentrating on the lyophilizer. Excess triethyl ammonium bicarbonate was removed from the sample by additional dilution with water and freezing, followed by lyophilization until the sample reached constant mass. Note that for this protocol, yields and stock solutions of the nucleoside triphosphates were prepared with the presumption that all final products would exist as tetra-triethylammonium salts. However, after additional, scrupulous lyophilization of analytical samples for the NMR analysis, we generally saw two to three triethylammoniums present in the final product.

#### Evaluation of prepared nucleoside triphosphates

Analytical HPLC was performed on an Agilent 1260 series LC system with diode array detection using a reversed phase method (mobile phase A: water, 400 mM HFIP, 15 mM TEA; mobile phase B: methanol, 400 mM HFIP, 15 mM TEA, unless otherwise specified). The best triphosphate resolution was obtained using a Waters XBridge Oligonucleotide BEH C18 column (130 Å, 2.5 µm, 4.6 mm × 50 mm). High-resolution mass spectra were obtained via ESI-MS-HiRes on a Thermo q-Exactive Plus spectrometer. ^1^H NMR (400 MHz on a Varian Mercury instrument) and ^31^P NMR (400 MHz on a Varian Mercury instrument) spectra were measured. Chemical shifts are reported relative to the central line of residual solvent.

### Reporting summary

Further information on research design is available in the Nature Portfolio Reporting Summary linked to this article.

## Online content

Any methods, additional references, Nature Portfolio reporting summaries, source data, extended data, supplementary information, acknowledgements, peer review information; details of author contributions and competing interests; and statements of data and code availability are available at 10.1038/s41587-024-02244-w.

## Supplementary information

Supplementary Information Supplementary Figs. 1–21 and Tables 1–3, enzyme sequences SEQ1–SEQ5, NMR analysis of 3′-*O*-allyl ether NTP and uncropped scans of gels from Supplementary figures.

Reporting Summary

Supplementary Data 1 Structures, molecular formulas, calculated masses and observed masses of mononucleotide extensions from indicated figures. A Microsoft Excel-based toolkit (named ezRNA Analyzer) to determine oligonucleotide exact mass is included.

## Source data

Source Data Fig. 1 Kinetic profile for each 3′-*O*-allyl ether NTP analyzed with denaturing gel electrophoresis; reaction samples were taken at 1, 5, 10, 20 and 30 min. Control reactions (N) included all reaction components except NTP.

Source Data Fig. 4 High-resolution gel electrophoresis to analyze the success of each cycle after the sequence was enzymatically synthesized with an imager set to collect Cy5 signal.

## Data Availability

Raw NMR data have been deposited at 10.7910/DVN/8XLE6P (ref. ^60^). Processed MALDI-TOF mass spectrometry data can be accessed at https://github.com/dan-wiegand/Enzymatic_RNA_Synthesis (ref. ^61^). Additional LC/MS data are available upon request from the corresponding authors. All data needed to reproduce the results of this study are available in the Article, online Methods and Supplementary Information. Source data are provided with this paper.
