# Supplementary material for: Template-independent enzymatic synthesis of RNA oligonucleotides
Source: Nat Biotechnol. 2024 Jul 12;43(5):762–72. doi: 10.1038/s41587-024-02244-w (PMC12084152; doi:10.1038/s41587-024-02244-w)

nature biotechnology

Supplementary information

https://doi.org/10.1038/s41587-024-02244-w

Template-independent enzymatic synthesis of RNA oligonucleotides

In the format provided by the authors and unedited

Table of Contents

Supplementary Figures

Fig. S1: Uncontrolled polymerization of natural NTPs by wildtype PUP and mutant variants.

Fig. S2: Incorporation of 3'-O-allyl-ATP using variable length initiator oligonucleotides.

Fig. S3: Incorporation of 3'-O-allyl-ATP using initiators with variable sequence compositions.

Fig. S4: LC/MS analysis of controlled, enzymatic extension reactions using 3'-O-allyl-NTPs.

Fig. S5: LC/MS analysis of 3'-O-allyl-NTP deblocking after controlled, enzymatic extension.

Fig. S6: Enzymatic processing of 3'-O-allyl-NTPs as a function of initiator concentration.

Fig. S7: Stepwise LC analysis of intermediates and final product for natural N+5* sequence.

Fig. S8: LC analysis of natural N+10* final oligonucleotide product synthesized in liquid phase.

Fig. S9: Assessment of controlled enzymatic synthesis using 2'-OMe modified 3'-O-allyl-NTPs.

Fig. S10: Assessment of controlled enzymatic synthesis using 2'-F modified 3'-O-allyl-NTPs.

Fig. S11: Assessment of controlled enzymatic synthesis using PS modified 3'-O-allyl-NTPs.

Fig. S12: Uncontrolled polymerization of therapeutically modified NTPs by PUP mutant variants

Fig. S13: Enzymatic incorporation of propargyl modified NTPs by PUP mutant variants.

Fig. S14: Installation of α-GalNAc-PEG3 ligand after incorporation of 3'-O-propargyl-NTPs.

Fig. S15: Initiator oligonucleotide cleavage using wildtype *E. coli* Endonuclease V variants.

Fig. S16: Phosphatase removal of 5'-phosphate groups from cleaved initiator oligonucleotides.

Fig. S17: Initial evaluation of 3'-O-allyl-ITP for initiator cleavage and substrate restoration.

Fig. S18: A stir-reactor apparatus for solid-phase enzymatic synthesis using CPG supports.

Fig. S19: Evaluation of 3'-O-allyl-NTP incorporation and product cleaved from CPG supports.

Fig. S20: Incorporation of 3'-O-allyl-ATP using hybrid initiators comprised of RNA/DNA bases

Fig. S21: Incorporation of natural and reversible terminator dNTPs by PUP mutant variants.

Tables

Table S1: Product mass summary for synthesis activities described in Fig. 2B, 2C & Fig. 3B, 3F.

Table S2: Product mass summary for synthesis activities described in Fig. 4A, 4B, & 4D.

Table S3: Product mass summary for synthesis activities described in Fig. 5C & 5D.

Enzyme AA Sequences

SEQ1: *S. pombe* Poly(U) Polymerase WT

SEQ2: *S. pombe* Poly(U) Polymerase H336R

SEQ3: *S. pombe* Poly(U) Polymerase H336R-N171A-T172S

SEQ4: *E. coli* Endonuclease V WT

SEQ5: *E. coli* Endonuclease V WT – N-Term. MBP Fusion

NMR Analysis of 3'-O-allyl ether NTP

NMR 1: 3'-O-allyl ether ATP – Proton NMR

NMR 2: 3'-O-allyl ether ATP – Phosphorous NMR

NMR 3: 3'-O-allyl ether UTP – Proton NMR

NMR 4: 3'-O-allyl ether UTP – Phosphorous NMR

NMR 5: 3'-O-allyl ether GTP – Proton NMR

NMR 6: 3'-O-allyl ether GTP – Phosphorous NMR

NMR 7: 3'-O-allyl ether CTP – Proton NMR

NMR 8: 3'-O-allyl ether CTP – Phosphorous NMR

Uncropped Scans of gels from Supplementary Figures

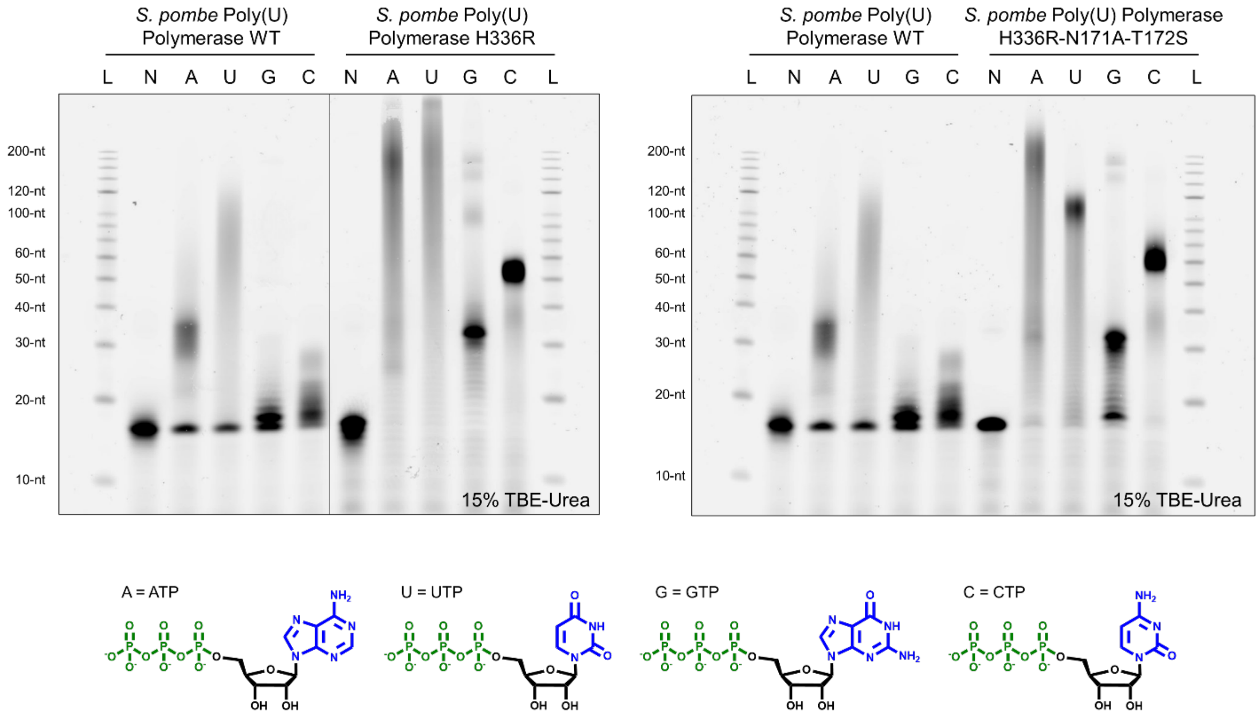

Fig. S1: A comparison of uncontrolled polymerization catalyzed by wild-type *S. pombe* Poly(U) Polymerase and two PUP mutant variants (H336R & H336R-N171A-T172S) using the natural, unblocked RNA nucleotides (A, U, G, C) under standard extension reaction conditions. Control reactions (N) contained all components except for nucleotide. A 200-nt ssDNA ladder was used to measure the approximate length of uncontrolled polymerization, which may appear as a smear or defined band if extension products coalesce around a particular oligonucleotide length. This direct comparison was performed once but is a compilation of several independent experimental repeats with similar results. The uncropped scan of this gel is supplied at the end of the document.

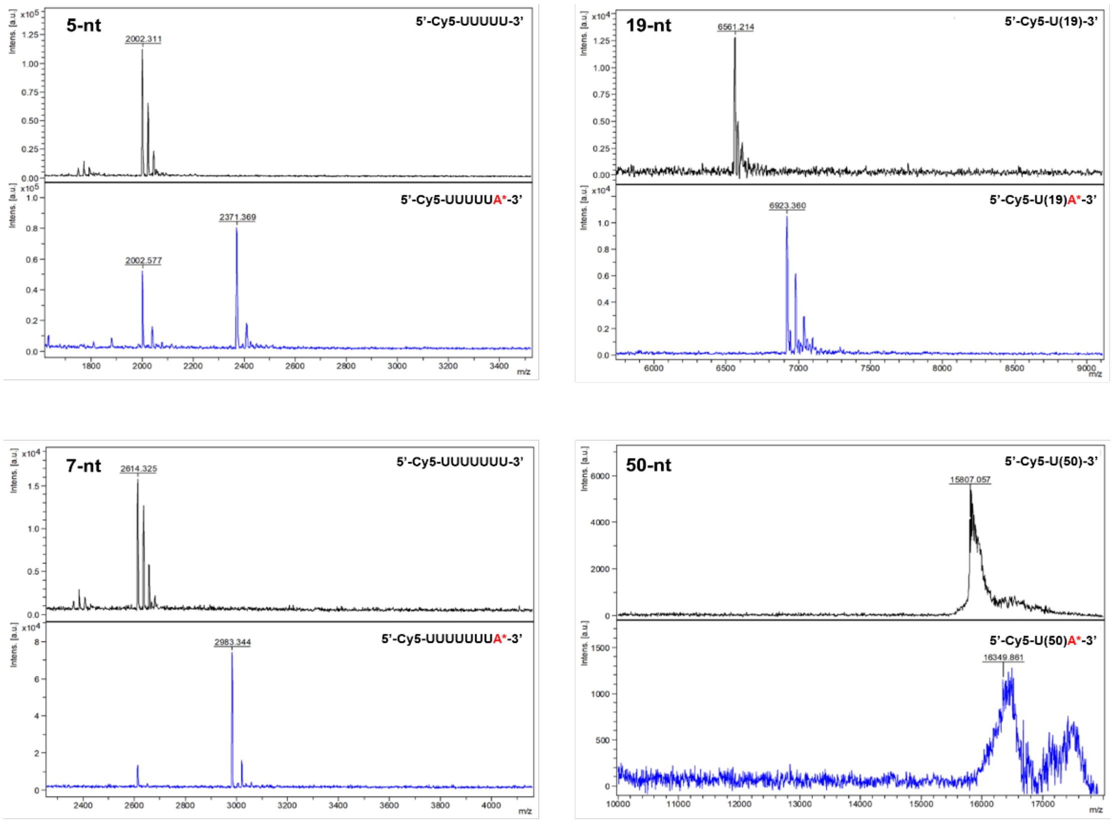

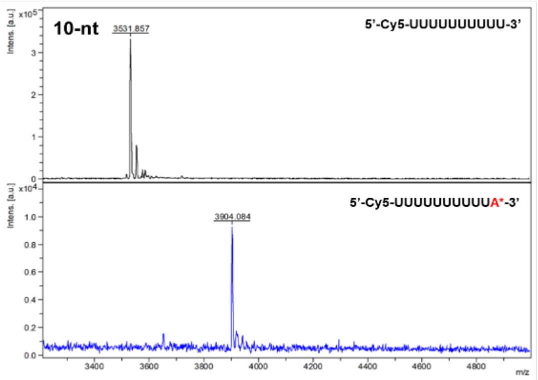

Observed MALDI-TOF Masses		
**Init. Length**	**N+0 (m/z)**	**N+A* (m/z)**
5-nt	2002.31	2371.36*
7-nt	2614.32	2983.34
10-nt	3531.85	3904.08
19-nt	6561.21	6823.36
50-nt	15807.05	16349.86

* Indicates partial conversion to the final product

Fig. S2A: An evaluation of Poly(U) Polymerase mutant variant H336R extension activity under standard reaction conditions with a 3'-O-allyl ether ATP reversible terminator in the presence of initiator oligonucleotides of variable length (5-, 7-, 10-, 19-, & 50-nt) using MALDI-TOF mass spectrometry. All initiators were comprised of a poly-U RNA sequence and feature a 5'- Cy5

modification. For each initiator oligonucleotide, mass spectra are shown before (top, black) and after (bottom, blue) enzymatic extension.

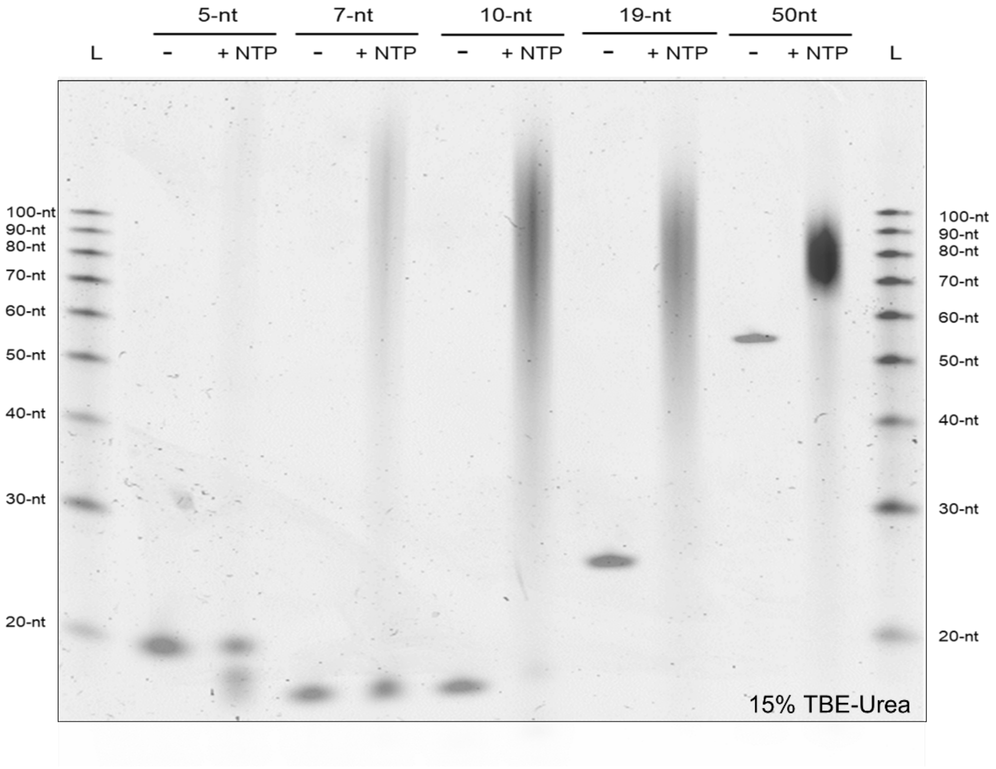

Fig. S2B: An evaluation of Poly(U) Polymerase mutant variant H336R extension activity under standard reaction conditions with an equimolar mixture of A, U, G, C natural NTPs in the presence of initiator oligonucleotide of variable length (5-, 7-, 10-, 19-, & 50-nt) using denaturing gel electrophoresis. All initiators were comprised of a poly-U RNA sequence and feature a 5'- Cy5 modification. For each initiator oligonucleotide, two lanes are shown on the gel: the negative (-) contained all reaction components except NTPs while the positive (+) were run with NTPs at a concentration of 1 mM (0.25 mM each base). A 100-nt ssDNA ladder was used to measure the approximate length of uncontrolled polymerization. The gel was imaged with the Cy3 and Cy5 channels consecutively, and these images were then overlayed. This experiment was conducted twice and both experiments showed similar results. The uncropped scan of this gel is supplied at the end of the document.

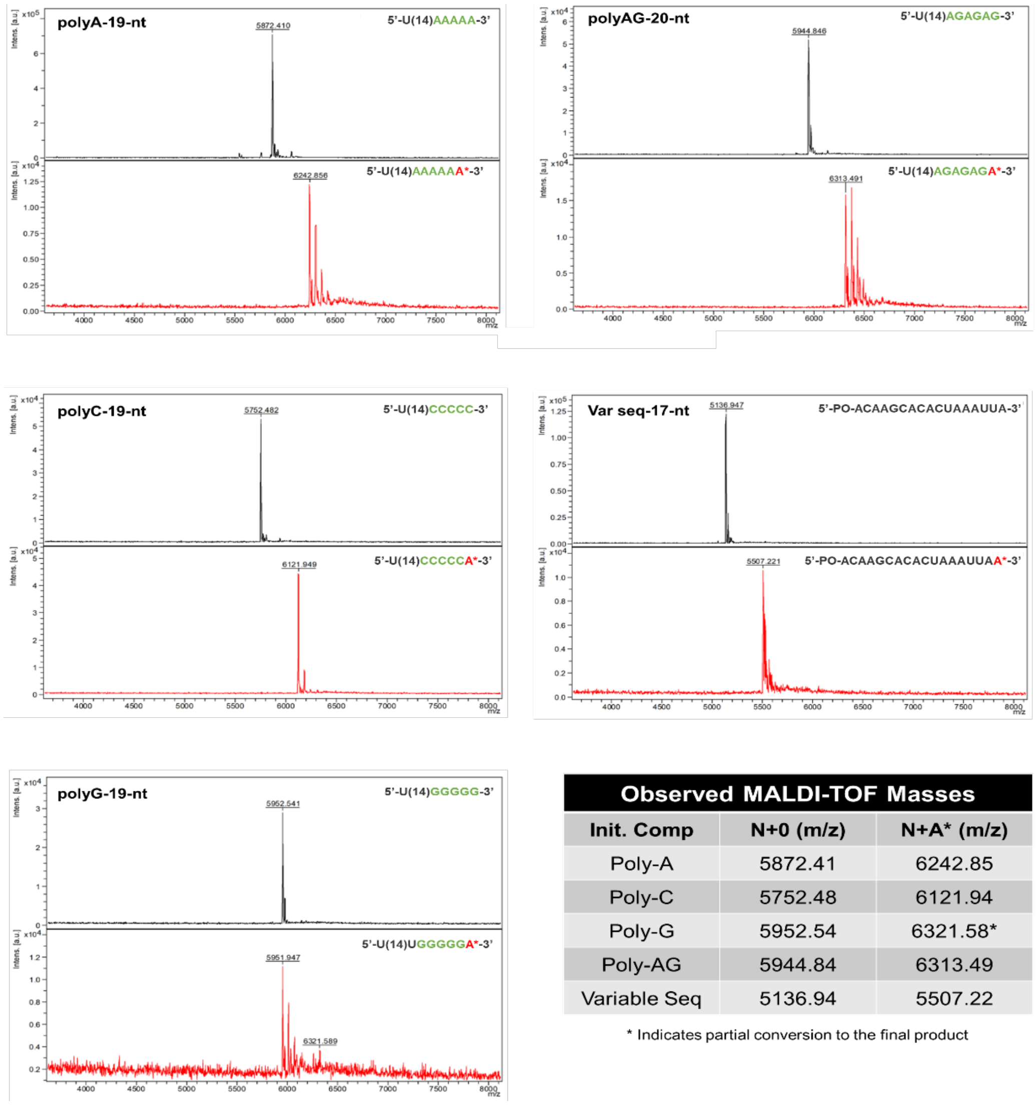

Observed MALDI-TOF Masses

Init. Comp	N+0 (m/z)	N+A* (m/z)
Poly-A	5872.41	6242.85
Poly-C	5752.48	6121.94
Poly-G	5952.54	6321.58*
Poly-AG	5944.84	6313.49
Variable Seq	5136.94	5507.22

* Indicates partial conversion to the final product

Fig. S3A: An evaluation of Poly(U) Polymerase mutant variant H336R extension activity under standard reaction conditions with a 3'-O- allyl ether ATP reversible terminator in the presence of initiator oligonucleotides of variable sequence composition using MALDI-TOF mass spectrometry. Initiators were rationally constructed to ascertain a measurement of enzymatic tolerance for different compositions and combinations of base sequences on the 3'- terminus of the oligonucleotide. The initiators tested featured a stretch of poly-A, -U, -G, -AG RNA bases in addition to an initiator of variable base composition with a 5'-phosphate (5'-PO). The initiators were of similar lengths, ranging from 17-nt to 20-nt. For each initiator oligonucleotide, mass spectra are shown before (top, black) and after (bottom, red) enzymatic extension.

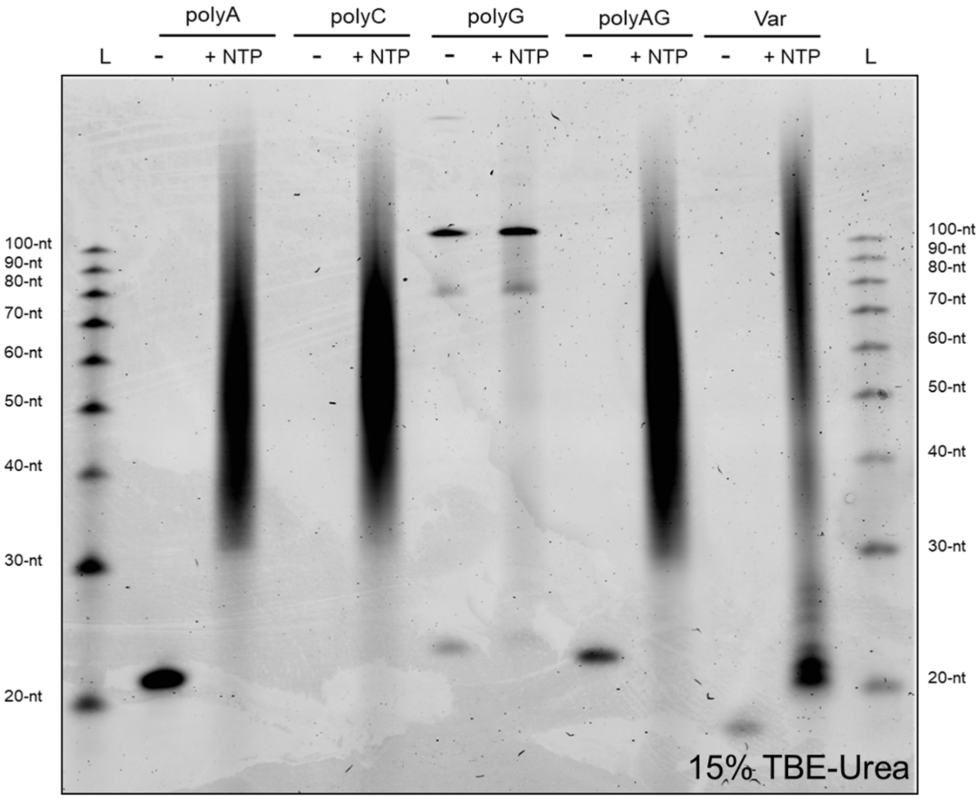

Fig. S3B: An evaluation of Poly(U) Polymerase mutant variant H336R extension activity under standard reaction conditions with natural NTPs in the presence of initiator oligonucleotide of variable sequence composition using denaturing gel electrophoresis. The initiators tested featured a stretch of poly-A, -U, -G, -AG RNA bases in addition to an initiator of random sequence with a 5'-PO modification. For each initiator oligonucleotide, two lanes are shown on the gel: the negative (-) contained all reaction components except NTPs while the positive (+) were run with NTPs at a concentration of 1 mM (0.25 mM each base). A 100-nt ssDNA ladder was used to measure the approximate length of uncontrolled polymerization. This experiment was conducted twice, and both experiments showed similar results. The uncropped scan of this gel is supplied at the end of the document.

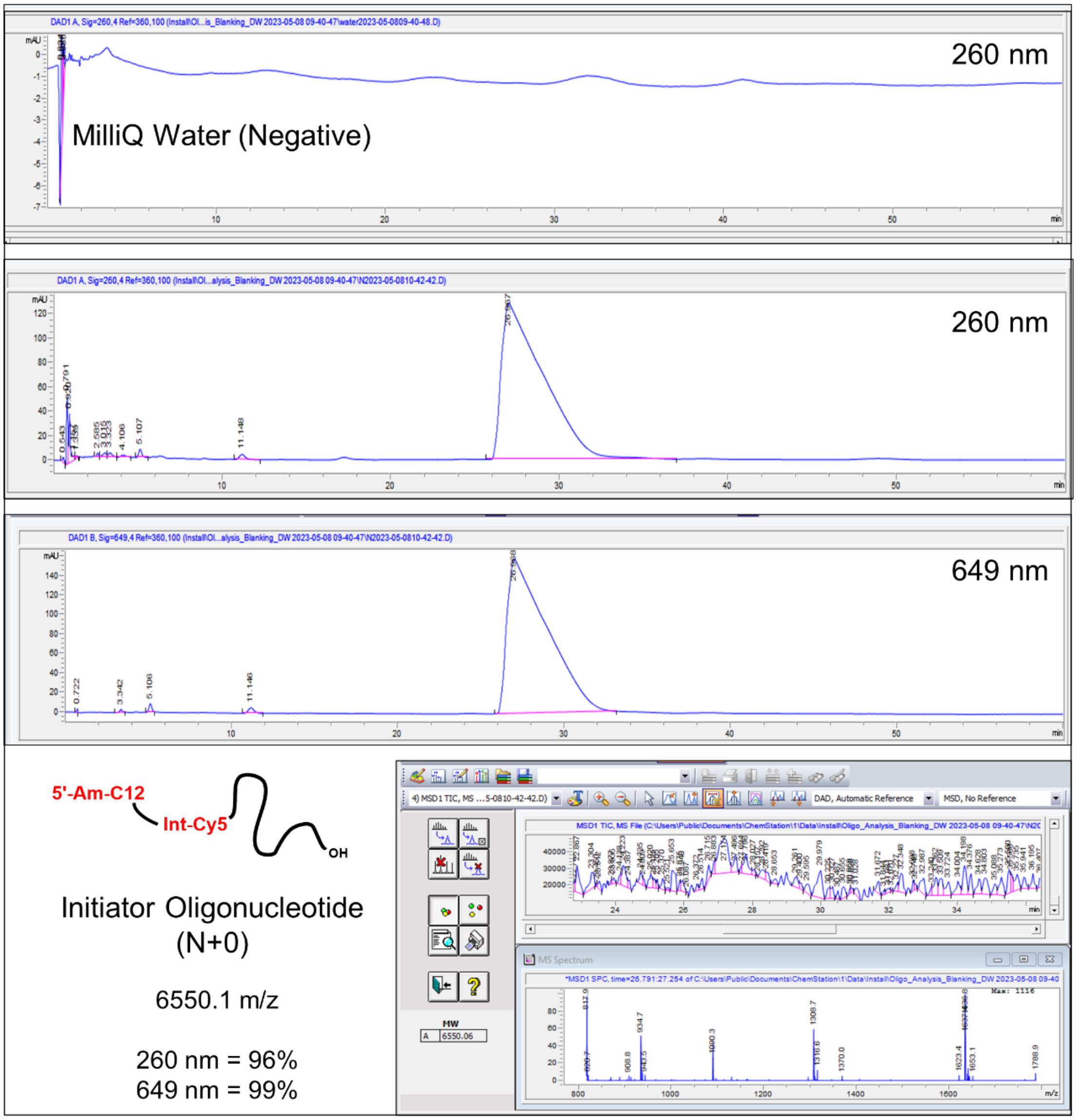

Fig. S4A: LC/MS analysis of initiator oligonucleotide starting material used to assess the coupling efficiency and isolated crude purity of controlled extension reactions with the PUP mutant and 3'-O- allyl ether NTP reversible terminator building blocks. The initiator oligonucleotide (N+0) is 19-nt in length and is comprised of a poly-U RNA sequence. The 5'- terminus features a primary amine and C12 linker as well as an internal Cy5 dye. Three HPLC chromatograms are shown: the first is a negative injection of MilliQ water, which all oligonucleotide samples are dissolved in, the second and third are an injection of approximately 2 nmol initiator with the DAD set to monitor 260 nm and 649 nm, respectively. The purity of the initiator was assessed at these wavelengths and found to be 260 nm = 96% and 649 nm = 99%, assuming similar response factors for the impurities as the oligonucleotide. The oligonucleotide mass was determined by deconvolution to be 6550.01 m/z, which corresponds to the expected mass.

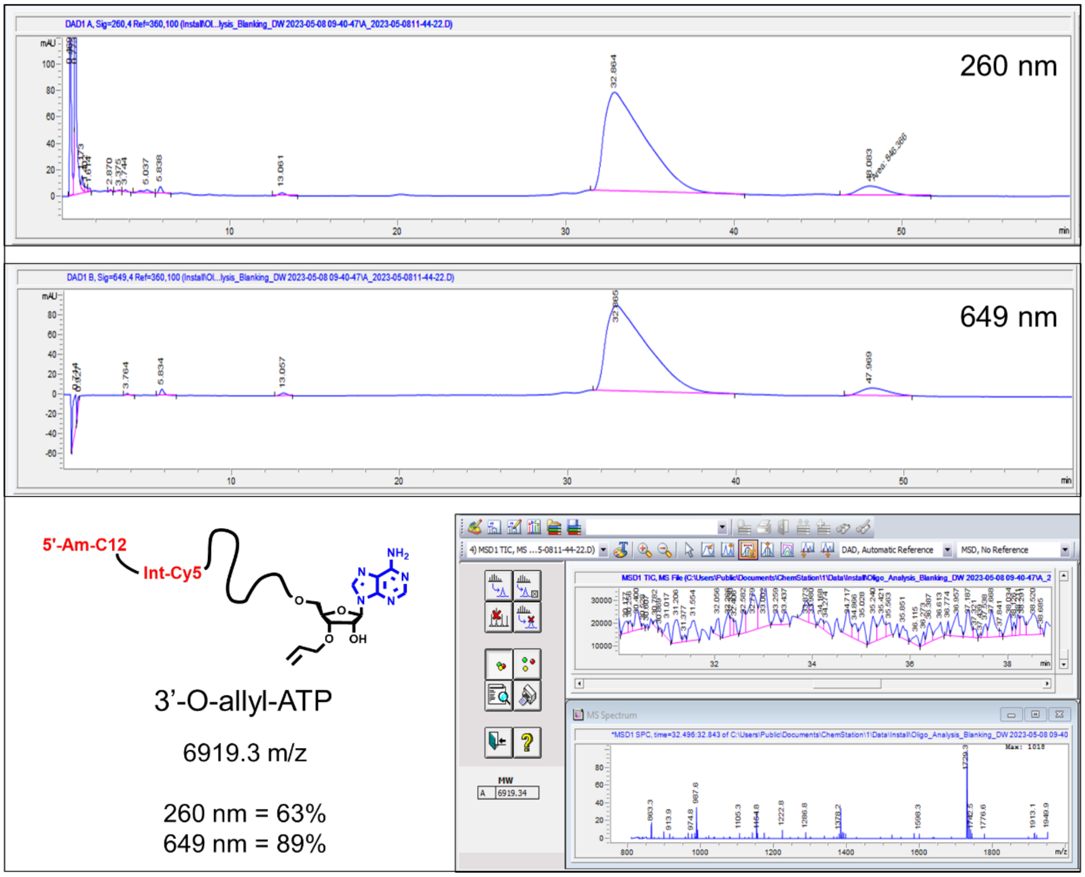

Fig. S4B: LC/MS analysis of controlled enzymatic extension using a **3'-O-allyl ether ATP** reversible terminator with the PUP mutant H336R, standard reaction conditions, and Cy5 19-nt initiator. Two HPLC chromatograms are shown that correspond to a single 2 nmol injection of the **N+1*** product at 260 nm and 649 nm. The isolated crude purity was found to be 260 nm = 63% and 649 nm = 89%. The oligonucleotide mass was determined by deconvolution to be 6919.3 m/z, which corresponds to the expected mass of the **N+A*** product. The second peak at 48 min likely corresponds to an **N+A*A*** impurity, which is about 10% of the total mass injected, and may have arisen through the formation of a non-natural 2'- / 5'- interlinkage at the terminal 2'-OH after initial N+A* incorporation.

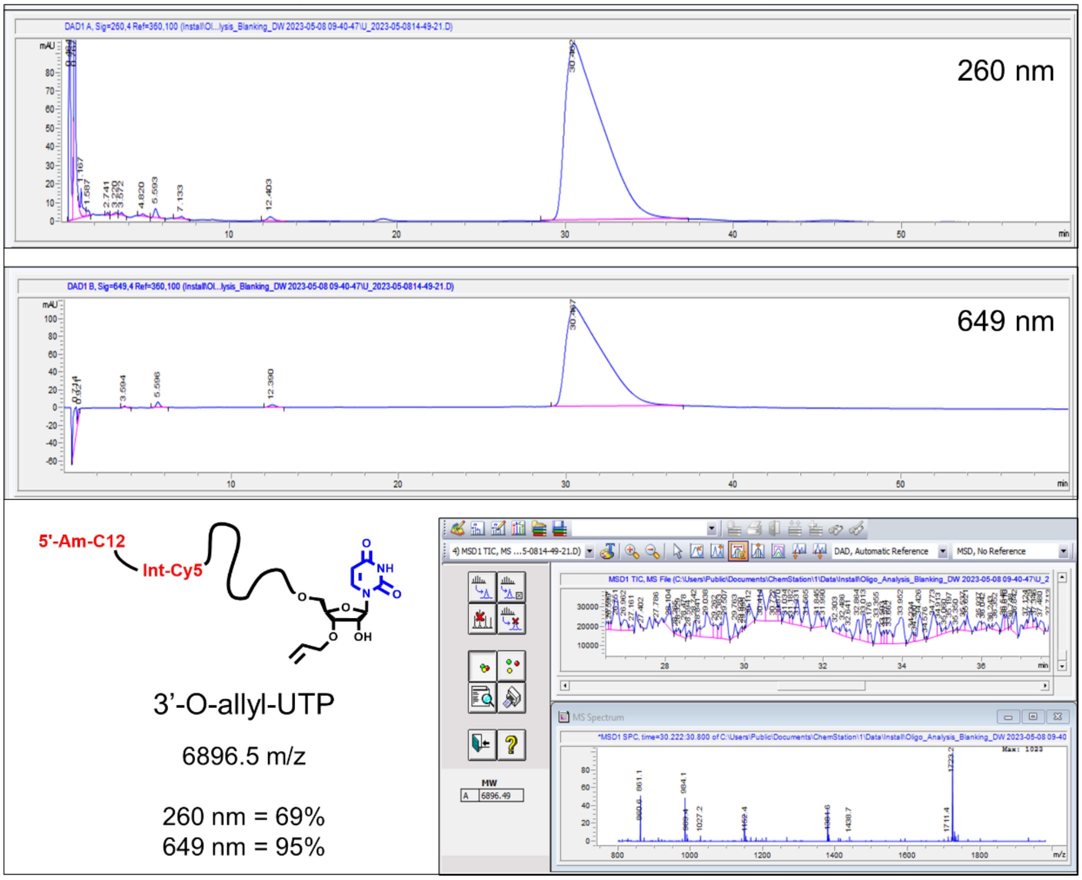

Fig. S4C: LC/MS analysis of controlled enzymatic extension using a **3'-O-allyl ether UTP** reversible terminator with the PUP H336R mutant, standard reaction conditions, and Cy5 19-nt initiator. Two HPLC chromatograms are shown that correspond to a single 2 nmol injection of the **N+1*** product at 260 nm and 649 nm. The integration of the main peak was found to be 260 nm = 69% and 649 nm = 95%. The oligonucleotide mass was determined by deconvolution to be 6896.5 m/z, which corresponds to the expected mass of the **N+U*** product.

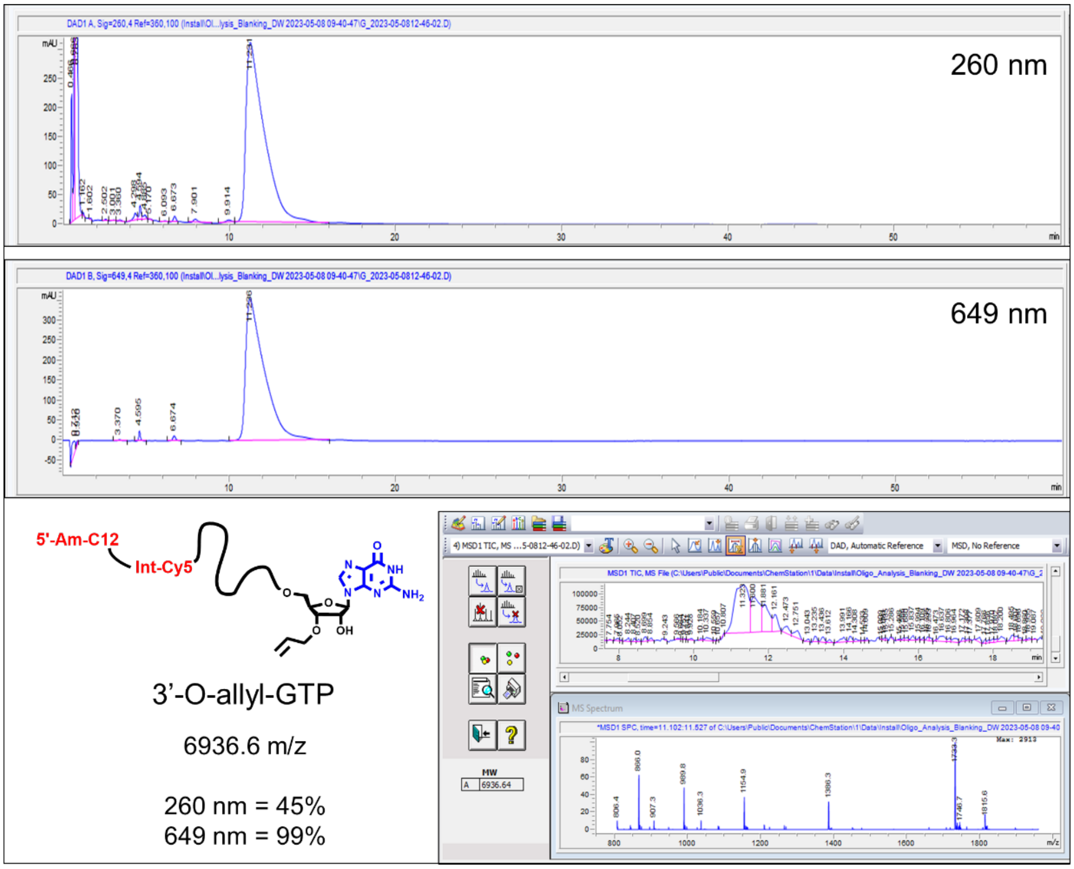

Fig. S4D: LC/MS analysis of controlled enzymatic extension using a **3'-O-allyl ether GTP** reversible terminator with the PUP mutant H336R, standard reaction conditions, and Cy5 19-nt initiator. Two HPLC chromatograms are shown that correspond to a single 2 nmol injection of the **N+1*** product at 260 nm and 649 nm. The integration of the main peak was found to be 260 nm = 45% and 649 nm = 99%. The oligonucleotide mass was determined by deconvolution to be 6896.5 m/z, which corresponds to the expected mass of the **N+G*** product.

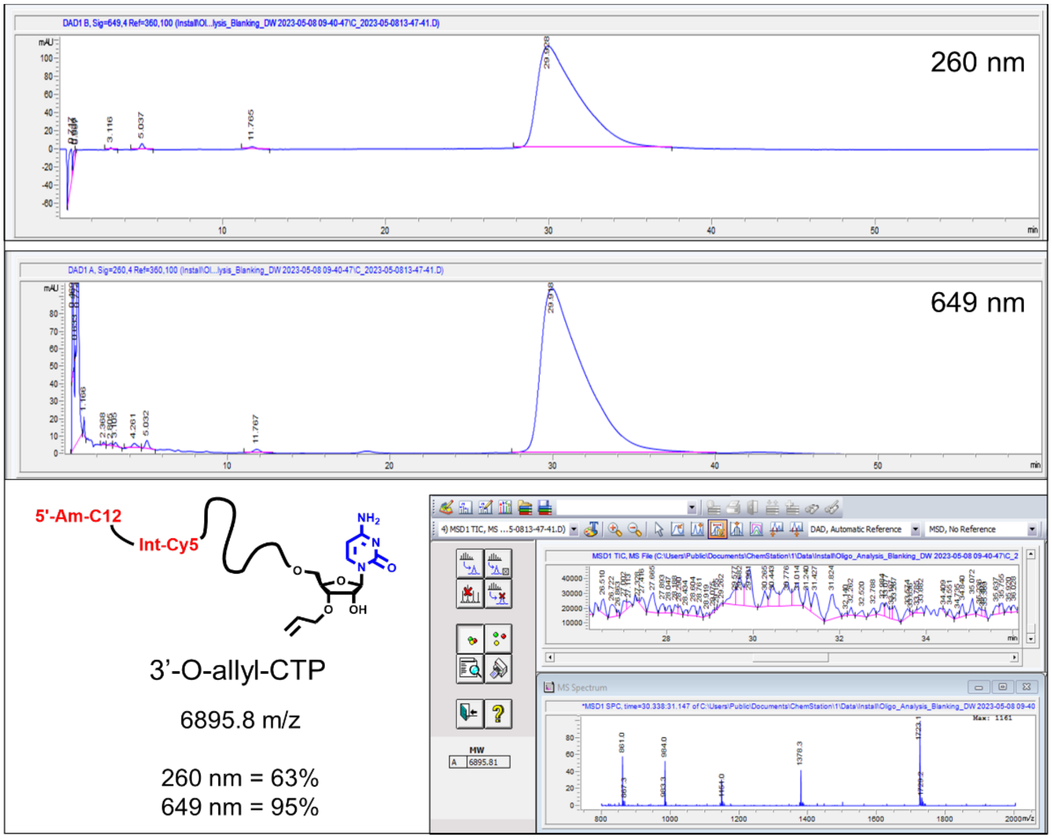

Fig. S4E: LC/MS analysis of controlled enzymatic extension using a **3'-O-allyl ether CTP** reversible terminator with the PUP mutant H336R, standard reaction conditions, and Cy5 19-nt initiator. Two HPLC chromatograms are shown that correspond to a single ~2 nmol injection of the **N+1*** product at 260 nm and 649 nm. The integration of the main peak was found to be 260 nm = 63% and 649 nm = 95%. The oligonucleotide mass was determined by deconvolution to be 6895.8 m/z, which corresponds to the expected mass of the **N+C*** product.

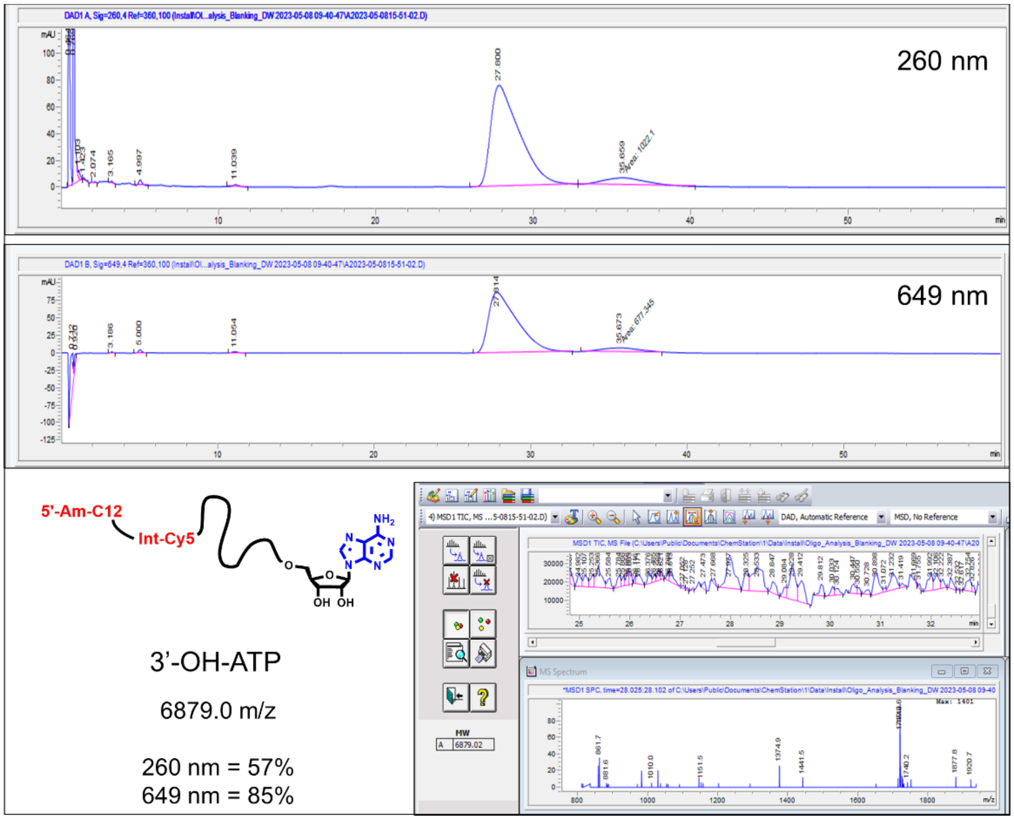

Fig. S5A: LC/MS analysis of 3'-O-allyl ether ATP deblocking post-enzymatic extension. Two HPLC chromatograms are shown that correspond to a single 2 nmol injection of the **N+1** product at 260 nm and 649 nm. The integration of the main peak was found to be 260 nm = 57% and 649 nm = 85%. The oligonucleotide mass was determined by deconvolution to be 6879.0 m/z, which corresponds to the expected mass of the **N+A** product. The second peak at 36 min likely corresponds to an **N+AA** impurity, which is about 15% of the total mass injected.

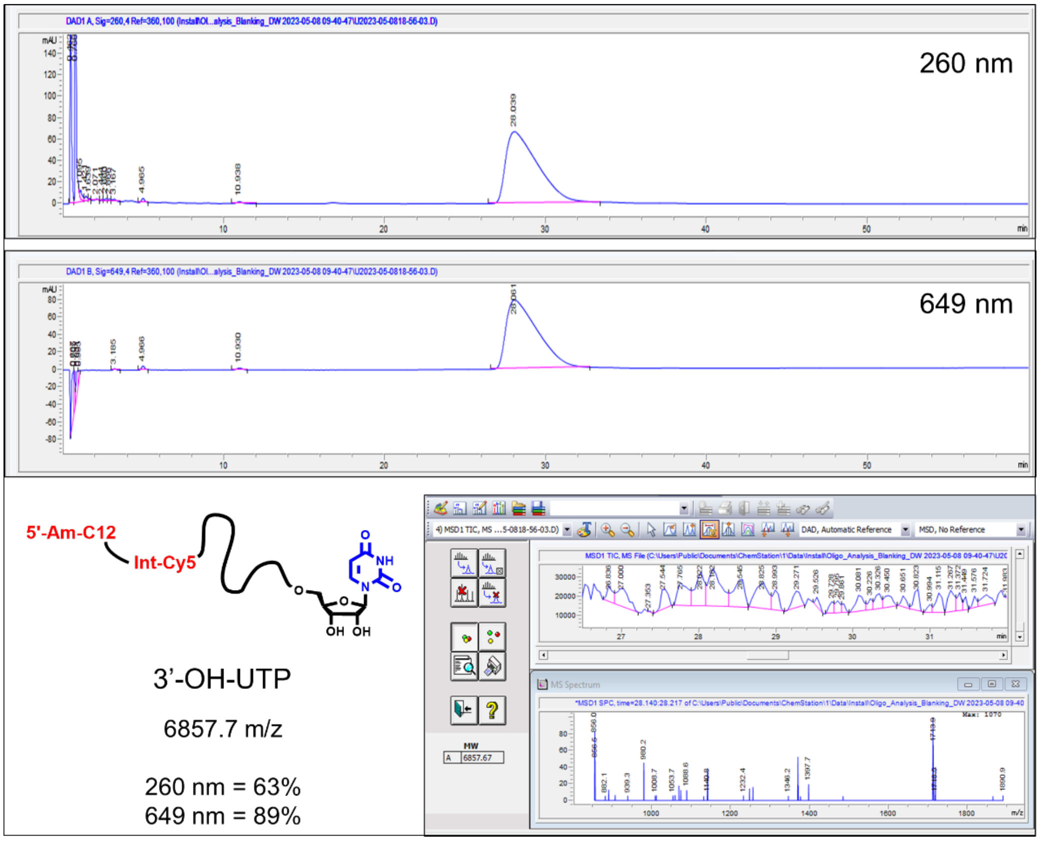

Fig. S5B: LC/MS analysis of 3'-O-allyl ether UTP deblocking post-enzymatic extension. Two HPLC chromatograms are shown that correspond to a single 2 nmol injection of the **N+1** product at 260 nm and 649 nm. The integration of the main peak was found to be 260 nm = 63% and 649 nm = 89%. The oligonucleotide mass was determined by deconvolution to be 6857.7 m/z, which corresponds to the expected mass of the **N+U** product.

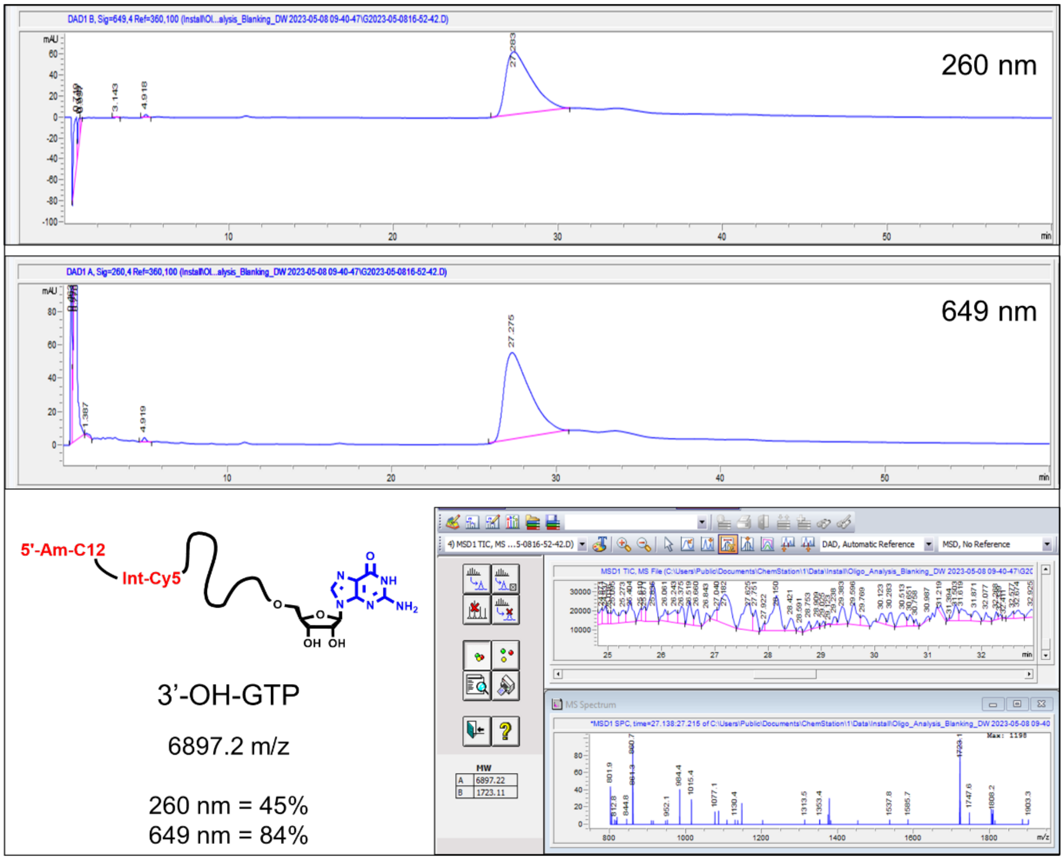

Fig. S5C: LC/MS analysis of 3'-O-allyl ether GTP deblocking post-enzymatic extension. Two HPLC chromatograms are shown that correspond to a single 2 nmol injection of the **N+1** product at 260 nm and 649 nm. The integration of the main peak was found to be 260 nm = 45% and 649 nm = 84%. The oligonucleotide mass was determined by deconvolution to be 6897.2 m/z, which corresponds to the expected mass of the **N+G** product.

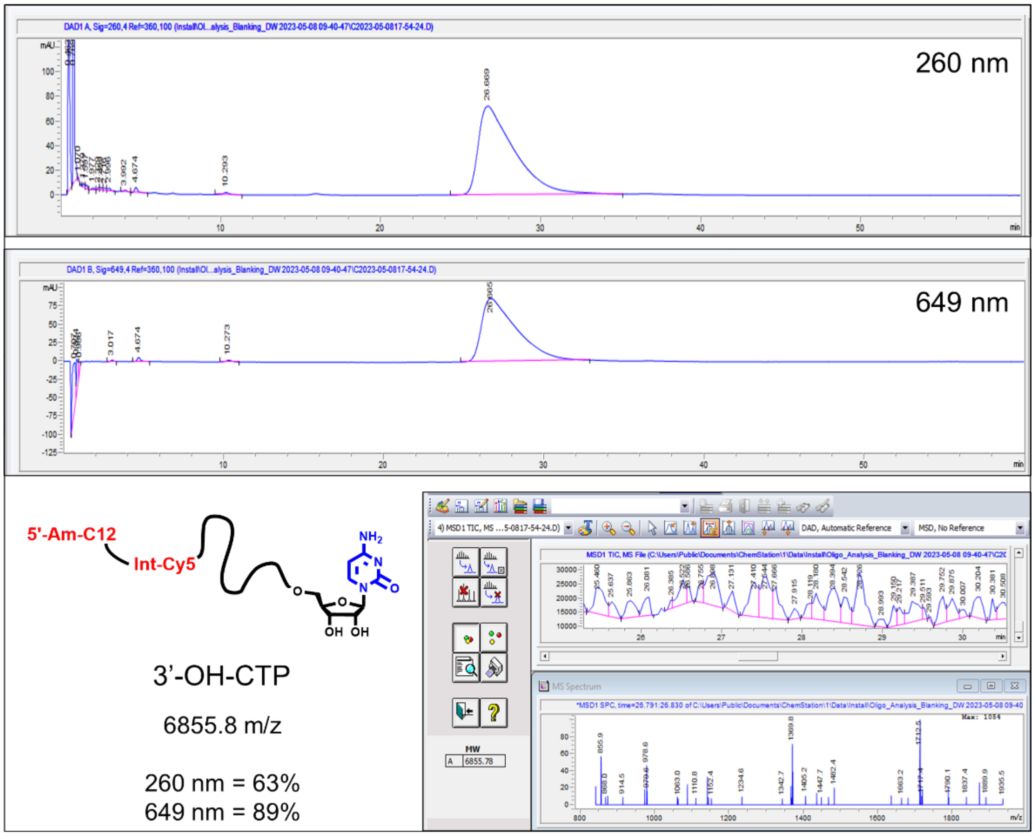

Fig. S5D: LC/MS analysis of 3'-O-allyl ether CTP deblocking post-enzymatic extension. Two HPLC chromatograms are shown that correspond to a single ~2 nmol injection of the **N+1** product at 260 nm and 649 nm. The integration of the main peak was found to be 260 nm = 63% and 649 nm = 89%. The oligonucleotide mass was determined by deconvolution to be 6855.8 m/z, which corresponds to the expected mass of the **N+C** product.

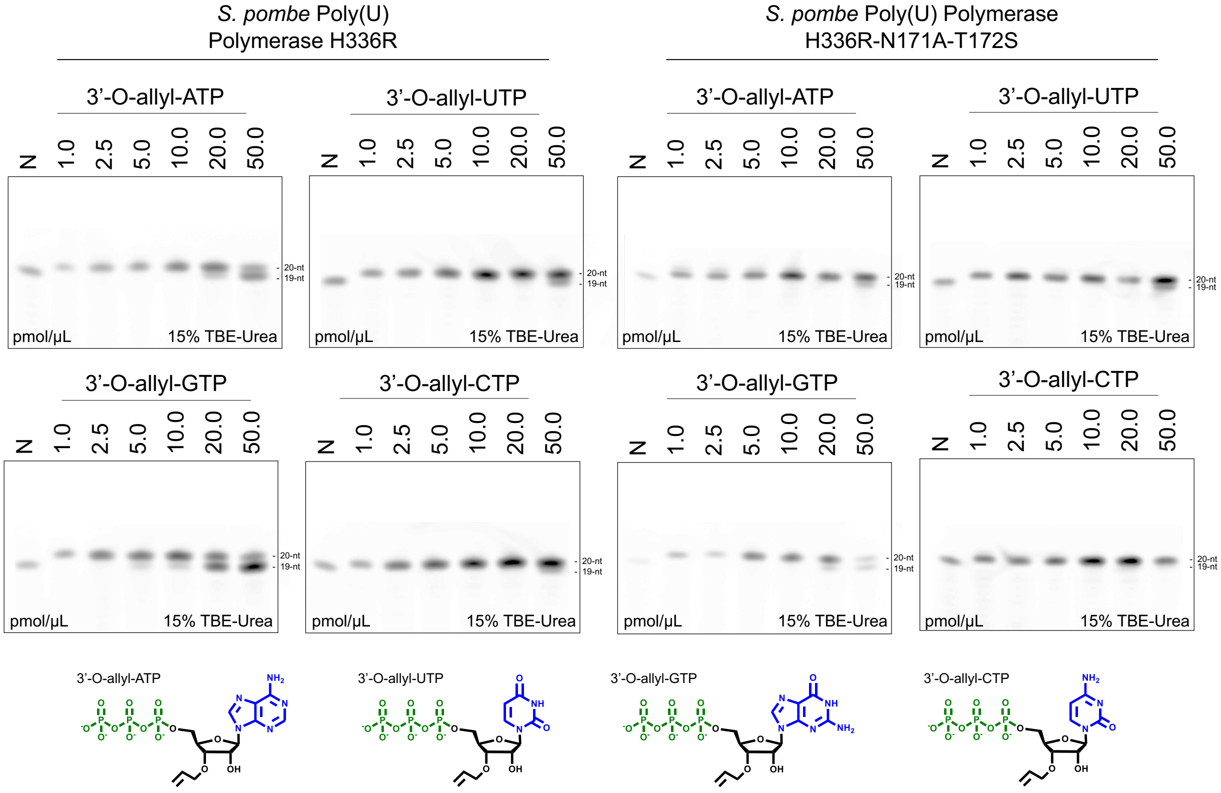

Initiator = /5AmMC12/rUrUrU/iCy5/rUrUrUrUrUrUrUrUrUrUrUrUrUrUrU ~ 19-nt

Fig. S6: An evaluation of enzymatic turnover with each 3'-O-allyl ether NTP set (A, U, G, C) by Poly(U) Polymerase mutant variants H336R and H336R-N171A-T172S using high resolution gel electrophoresis. Reactions were carried out using standard conditions, a fixed volume of 10 µL and a fixed incubation time of 30 minutes. The concentration of initiator oligonucleotide ranged from 1.0 pmol/µL to 50.0 pmol/µL (total reaction input of 10 pmol to 500 pmol). Post incubation with the enzyme mutant variants, the efficiency of reversible terminator NTP incorporation was determined using denaturing gel electrophoresis. Control reactions (N) included all components except NTP. This direct comparison was performed once but represents a compilation of several independent experimental repeats with similar results. The uncropped scan of this gel is supplied at the end of the document.

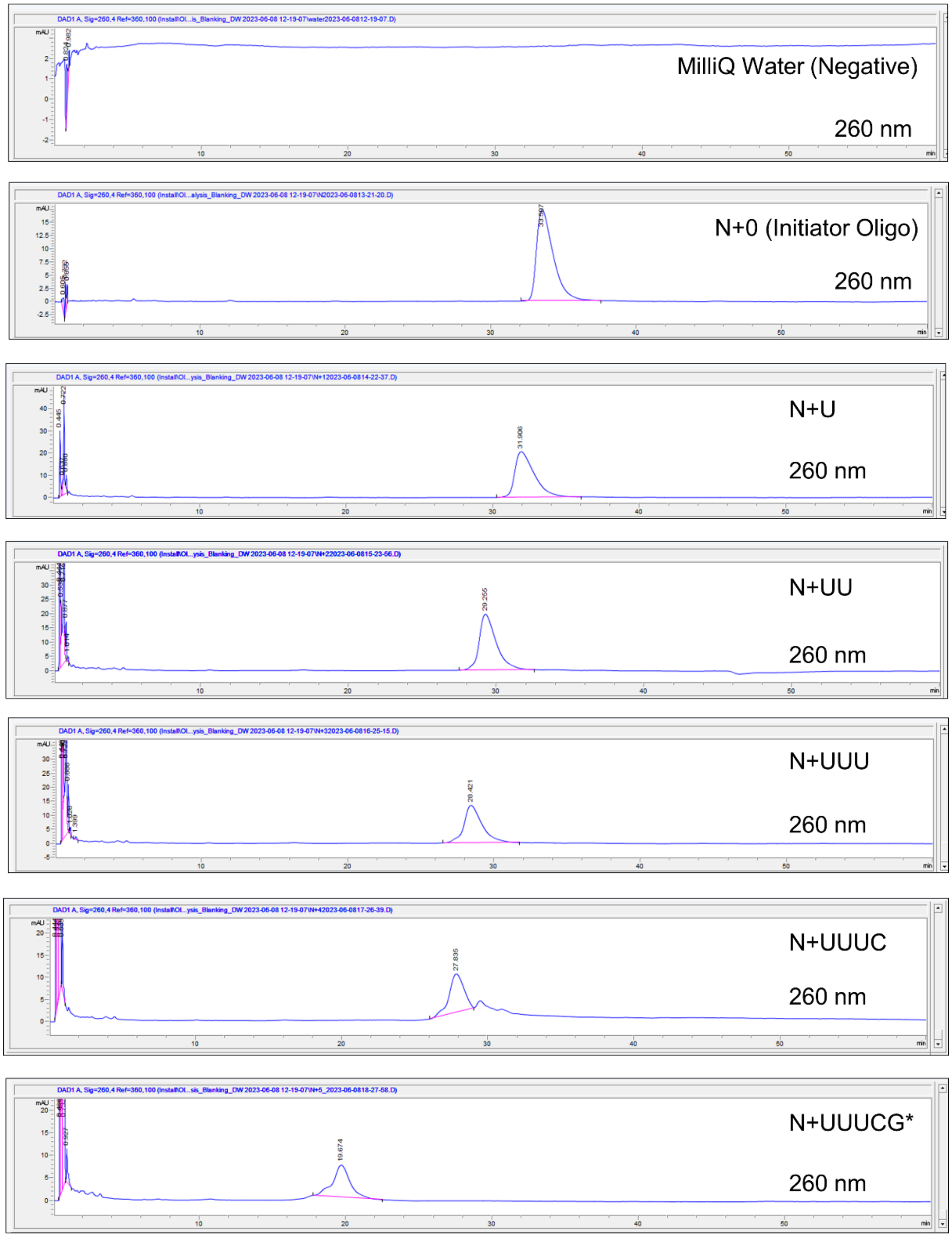

Fig. S7A: LC analysis of synthesis intermediates and final product generated when performing the controlled enzymatic synthesis of an **N+5*** RNA oligonucleotide with the sequence N+U-U-U-C-G*. Approximately ≤0.5 nmol of oligonucleotide intermediate and final product was injected onto the HPLC system. The isolated crude purity for each sample was determined by monitoring 260

nm and integrating over the chromatogram. A negative control consisting of MilliQ water was also included, which all oligonucleotide samples were dissolved in.

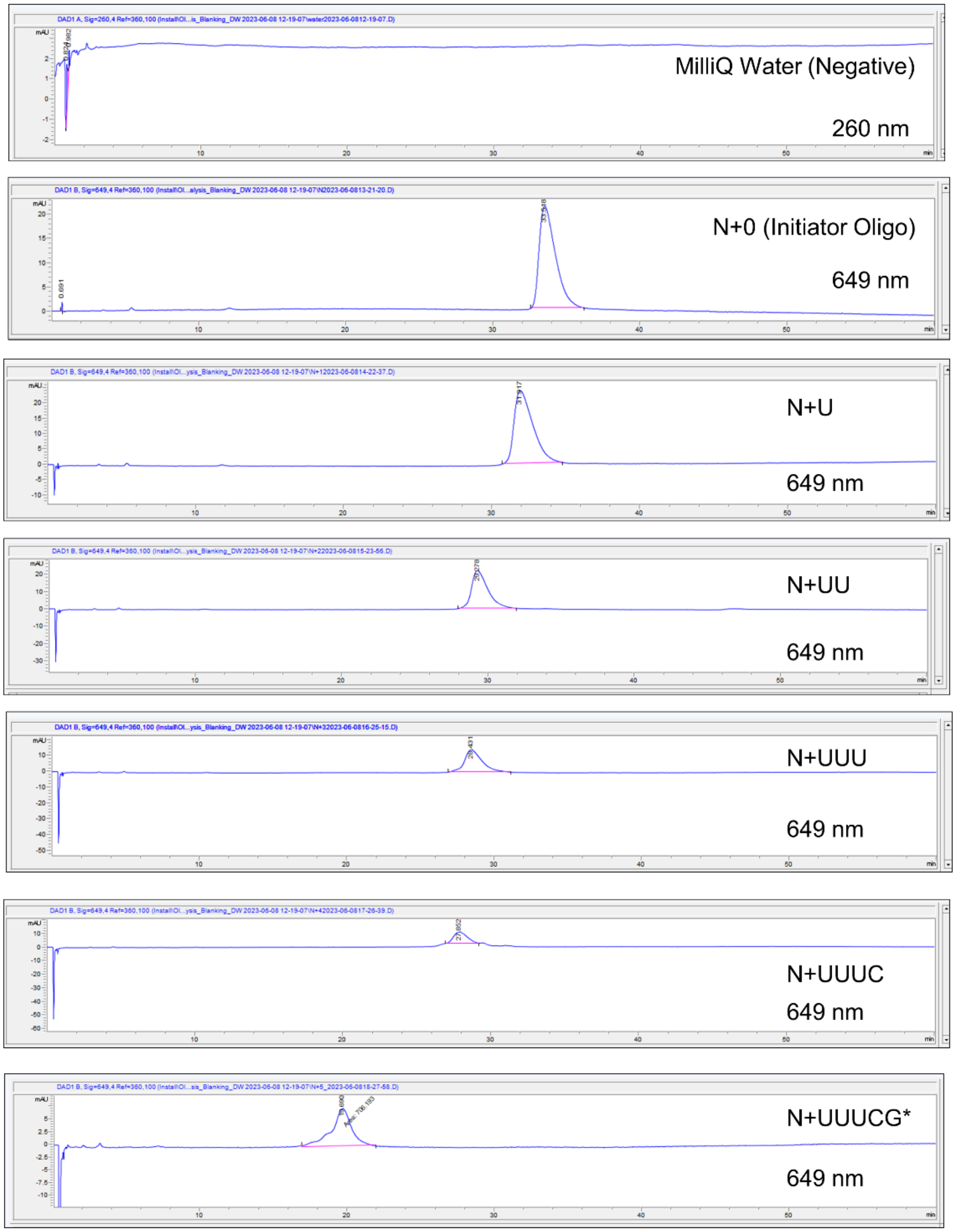

Fig. S7B: LC analysis of cycle intermediates and final product generated when performing the controlled enzymatic synthesis of an **N+5*** oligonucleotide with the natural 2'-OH RNA sequence N+U-U-U-C-G*, where N is a 19-nt Cy5 labeled initiator oligonucleotide. Approximately ≤0.5 nmol of oligonucleotide intermediate and final product was injected onto the HPLC system. The isolated crude purity for each sample was determined by monitoring 649 nm and integrating over

the chromatogram. A negative control consisting of MilliQ water was also included, which all oligonucleotide samples were dissolved in.

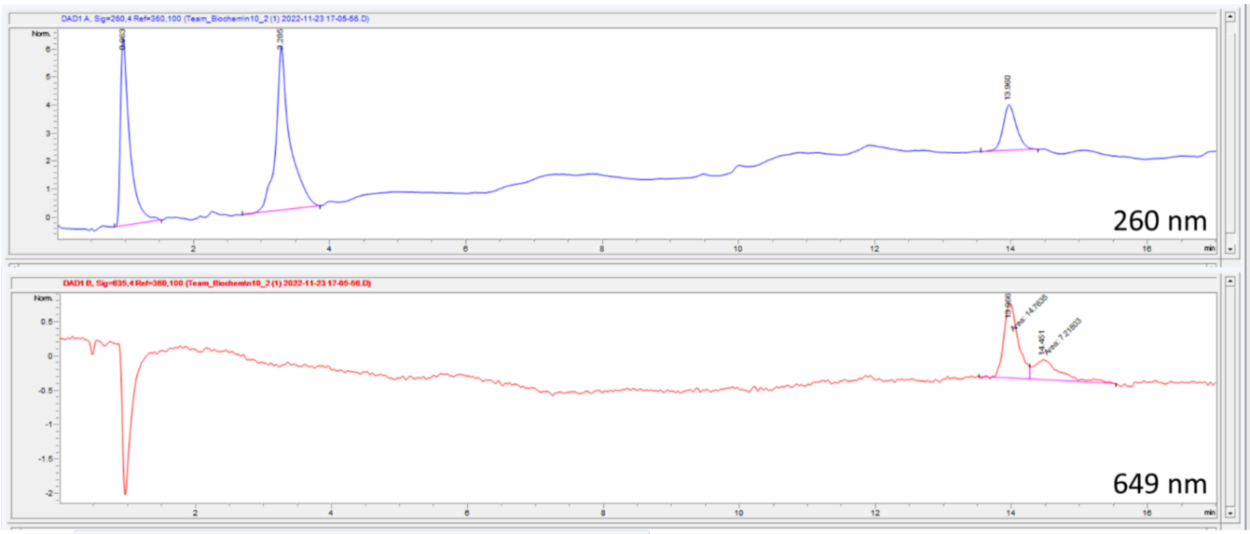

#	Time	Type	Area	Height	Width	Area%	Symmetry
1	0.963	BB	57.6	6	0.1331	37.154	0.368
2	3.285	BB	76.9	5.3	0.194	49.624	0.641
3	13.96	BB	20.5	1.5	0.2065	13.222	0.781

260 nm

#	Time	Type	Area	Height	Width	Area%	Symmetry
1	13.966	MF	14.8	9.9E-1	0.2494	67.163	0.7
2	14.451	FM	7.2	2.7E-1	0.4509	32.837	0.454

649 nm

Fig. S8. LC analysis of final product generated by controlled enzymatic synthesis of an **N+10*** oligonucleotide with the natural 2'-OH RNA sequence N+A-C-A-C-C-U-U-A-A-C*, where N is a 19-nt Cy5 labeled initiator oligonucleotide. Approximately 50 pmol of oligonucleotide final product was injected onto the HPLC system. The isolated crude purity (67.1%) was determined by monitoring 649 nm and integrating over the chromatogram. The apparent purity of the product as determined by 260 nm is 13.2%.

.

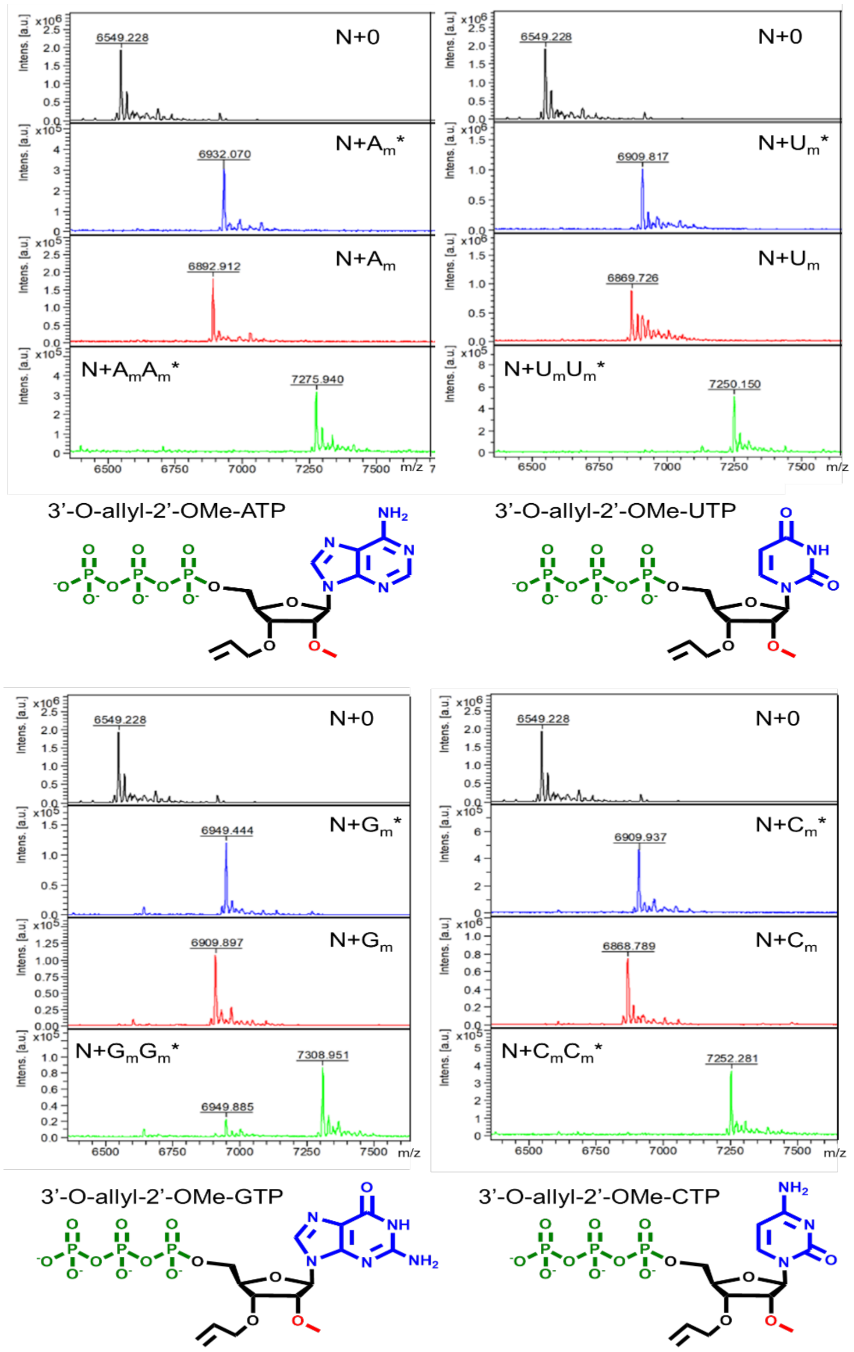

Fig. S9: An assessment of controlled, enzymatic oligonucleotide synthesis using the 2'-OMe modified 3'-O-allyl ether reversible terminator NTP set (A, U, G, C) with PUP mutant variant H336R. Extension reactions were carried out using standard reaction conditions using a Cy5 labeled 19-nt initiator oligonucleotide. MALDI-TOF mass spectrometry was used to assess the efficiency of initiator conversion to the **N+1*** product, allyl ether deblocking and then conversion to the **N+2*** product from **N+1**.

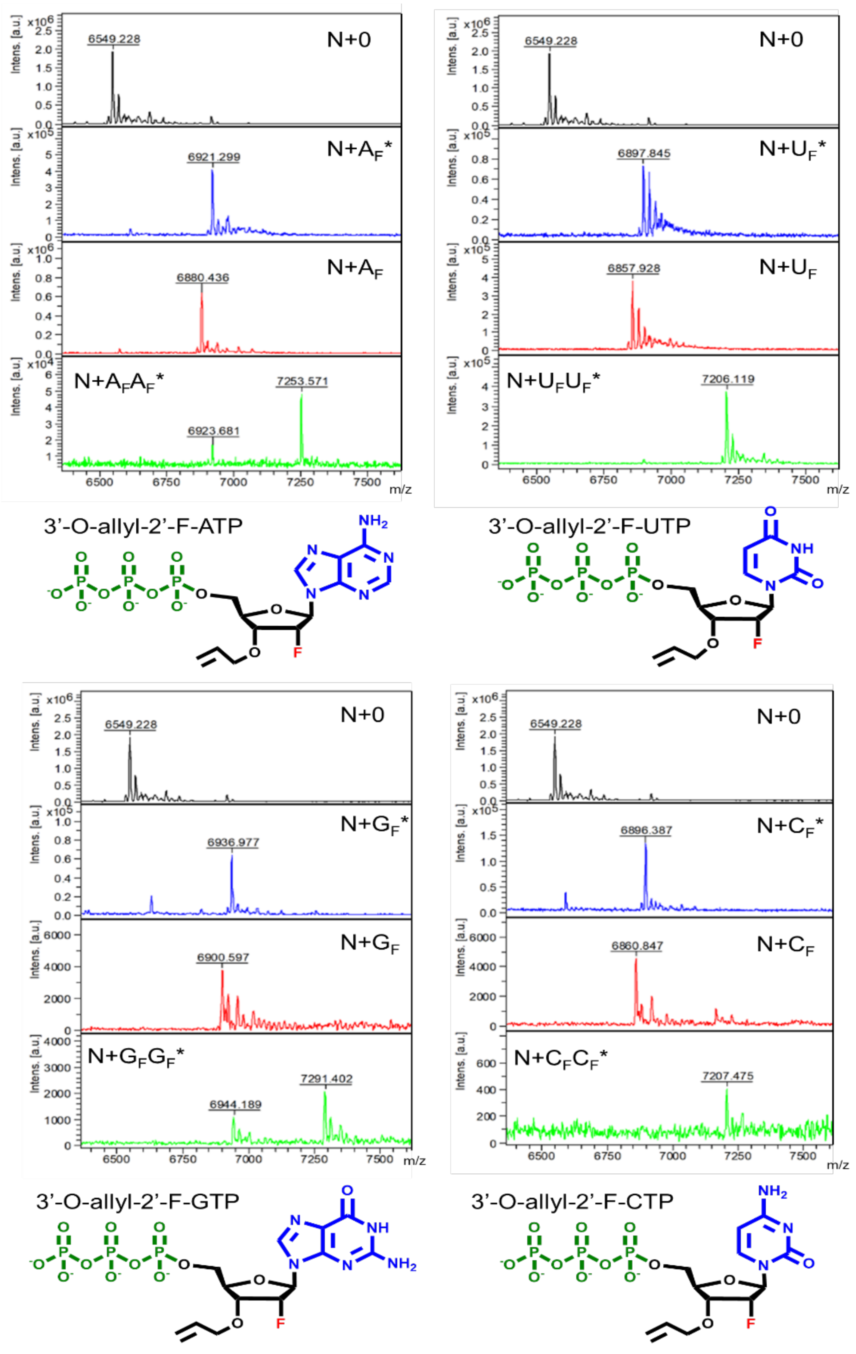

Fig. S10: An assessment of controlled, enzymatic oligonucleotide synthesis using the 2'-F modified 3'-O-allyl ether reversible terminator NTP set (A, U, G, C) with PUP mutant variant H336R. Extension reactions were carried out using standard reaction conditions using a Cy5 labeled 19-nt initiator oligonucleotide. MALDI-TOF mass spectrometry was used to assess the efficiency of initiator conversion to the **N+1*** product, allyl ether deblocking and then conversion to the **N+2*** product from **N+1**.

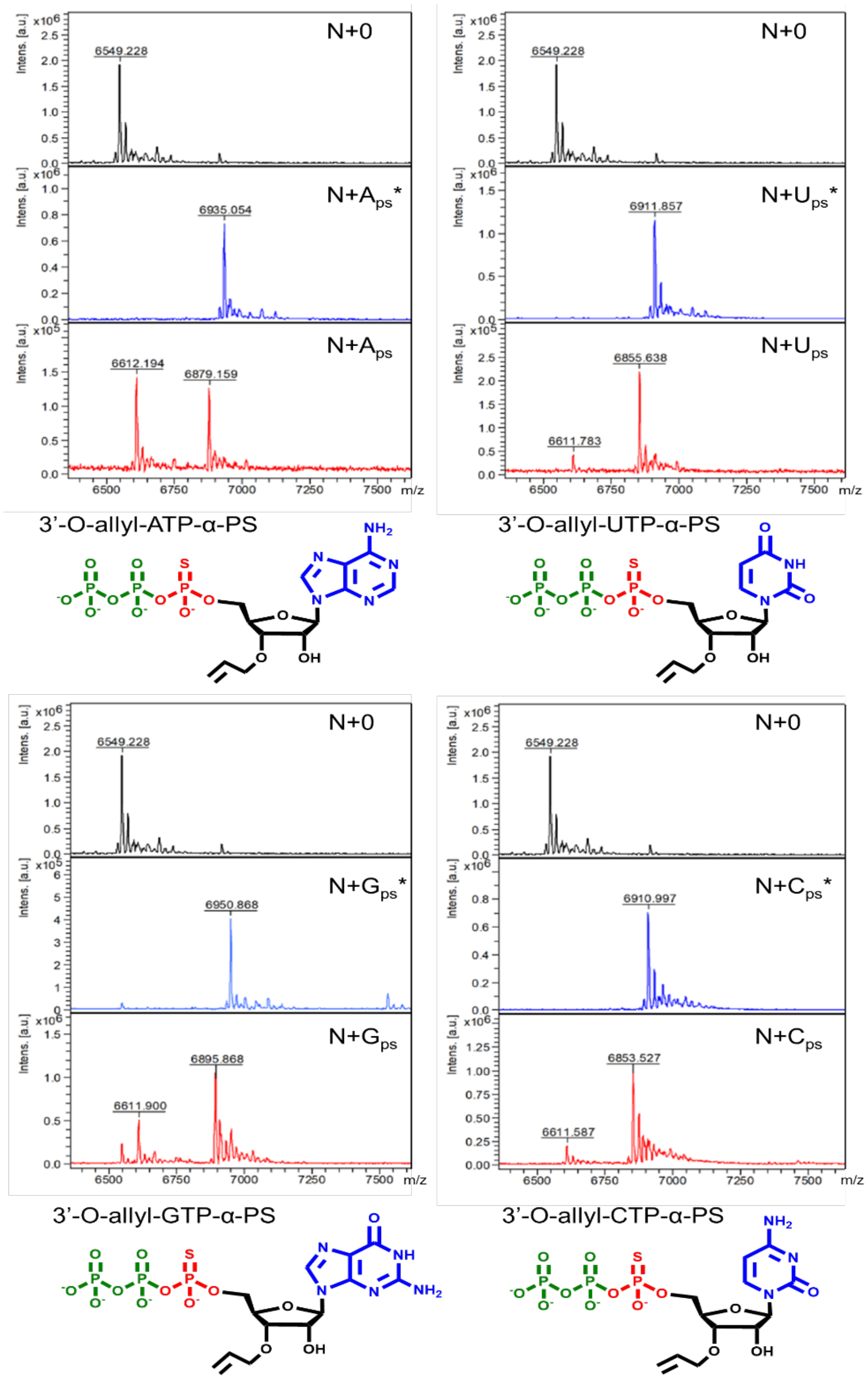

Fig. S11: An assessment of controlled, enzymatic oligonucleotide synthesis using the α-phosphorothioate (α-PS) modified 3'-O-allyl ether reversible terminator NTP set (A, U, G, C) with PUP mutant variant H336R. Extension reactions were carried out using standard reaction conditions using a Cy5 labeled 19-nt initiator oligonucleotide. MALDI-TOF mass spectrometry was used to assess the efficiency of initiator conversion to the **N+1*** product and allyl ether deblocking to **N+1**.

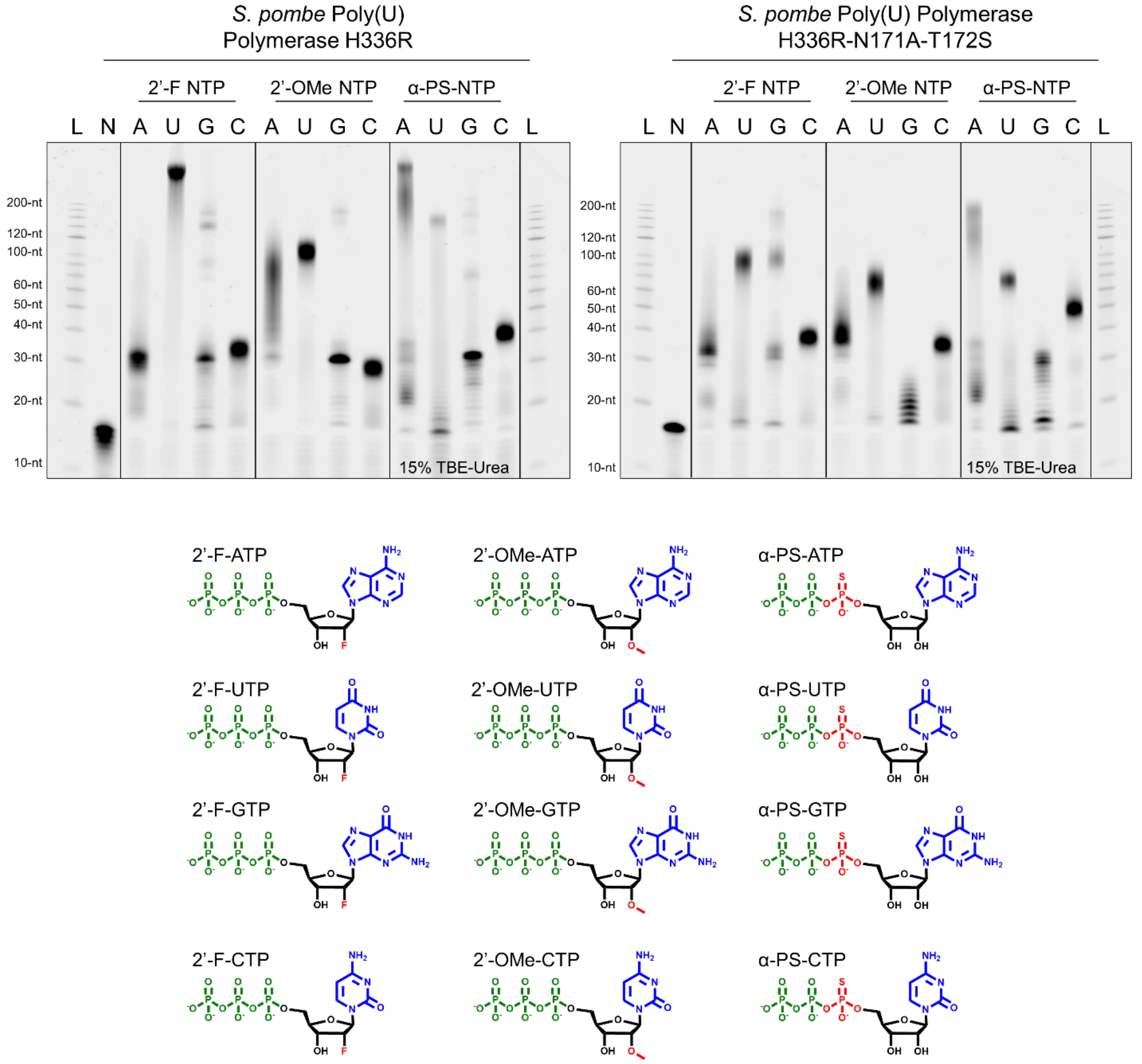

Fig. S12: An analysis of Poly(U) Polymerase mutant variant uncontrolled polymerization activity in the presence of unblocked 2’-fluoro (2’-F), 2’-methoxy (2’-OMe) and alpha-phosphorothioate (α-PS). Modified NTPs were incubated with both PUP mutant variants under standard reaction conditions using a Cy5 labeled 19-nt initiator oligonucleotide. The resultant extension products were assessed using denaturing gel electrophoresis using a 200-nt ssDNA ladder to measure the overall length of uncontrolled polymerization. This direct comparison was performed once but represents a compilation of several independent experimental repeats with similar results. The uncropped scan of this gel is supplied at the end of the document.

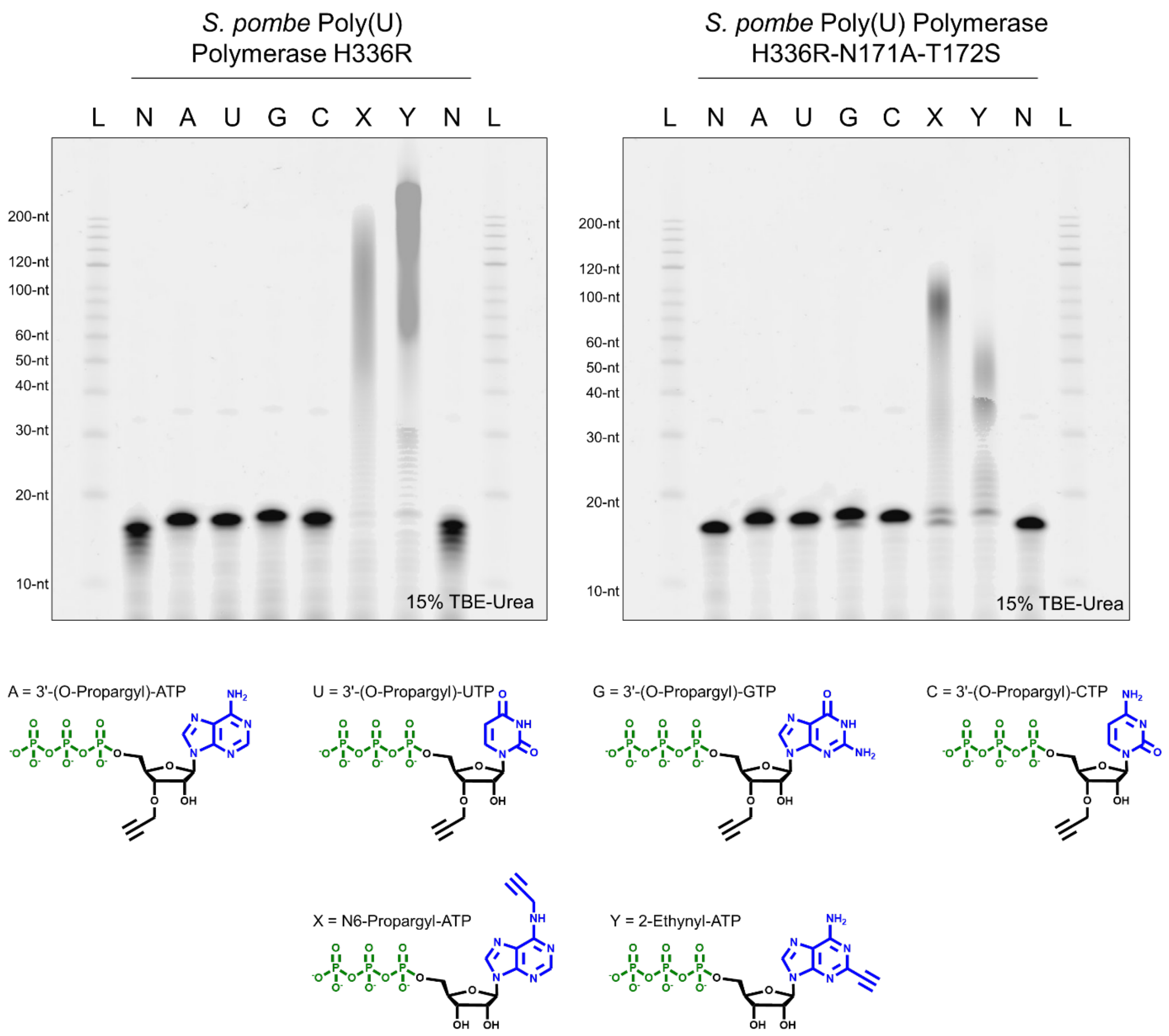

Fig. S13: An analysis of Poly(U) Polymerase mutant variant activity in the presence of propargyl modified nucleotides including a set of 3'- propargyl ether NTPs (A, U, G, C) as well as the base modified N6-propargyl-ATP and 2-Ethynyl-ATP. These modified NTPs were incubated with both PUP mutant variants under standard reaction conditions using a Cy5 labeled 19-nt initiator. The resultant extension products were assessed using denaturing gel electrophoresis with a 200-nt ssDNA ladder to measure the overall length of uncontrolled polymerization. Control reactions (N) contained all components except NTP. This experiment was conducted once but extension results found here are similar to those indicated in Fig. 4B (MALDI-TOF analysis of 3'- propargyl ether NTP incorporation). The uncropped scan of this gel is supplied at the end of the document.

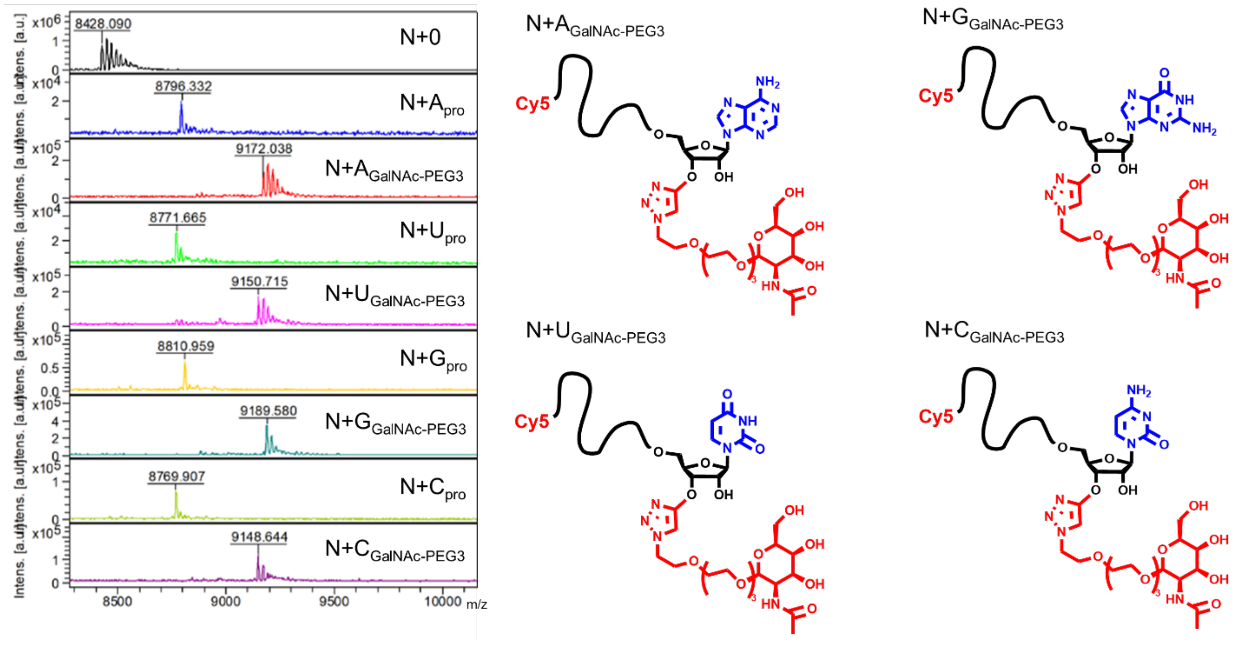

Fig. S14: An assessment of oligonucleotide labeling with an α-GalNAc-PEG3-Azide ligand with click chemistry following installation of a 3'- terminal propargyl group with PUP mutant variant H336R under standard reaction conditions and a 25-nt poly-U RNA initiator oligonucleotide modified with a 5'- Cy5. The complete set of 3'- propargyl ether NTPs (A, U, G, C) were evaluated in this activity. MALDI-TOF mass spectrometry was used to verify the conversion of the initiator oligonucleotide to the various **N+1$_{\text{pro}}$** products as well as determine the extent of labeling with α-GalNAc-PEG3 to produce **N+1$_{\text{GalNAc-PEG3}}$** oligonucleotides.

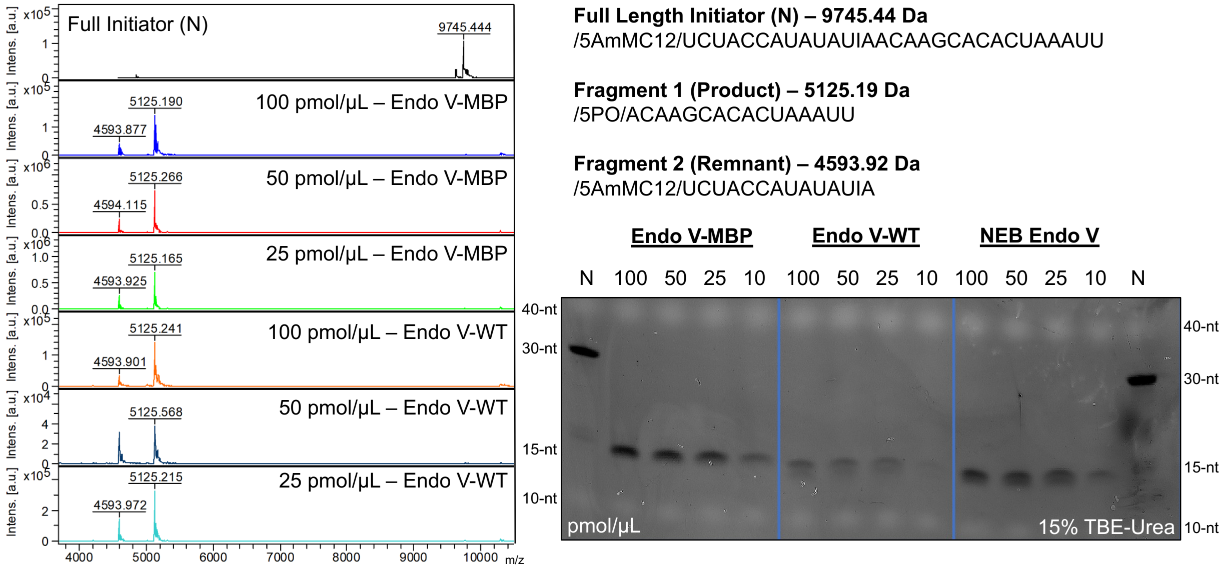

Fig. S15: An evaluation initiator oligonucleotide cleavage using Endonuclease V variants (wild-type and a fusion with an N-terminal maltose-binding protein (MBP)) expressed in-house and sourced commercially (NEB). MALDI-TOF mass spectrometry and denaturing gel electrophoresis was used to test cleavage robustness for each Endonuclease V variant. From the full-length initiator (N), it is expected that two fragments are formed: (1) the product at 5125.19 Da and (2) the remnant at 4593.92 Da. These were observed to have been formed when incubated with each Endonuclease V variant under standard cleavage reaction conditions. Similar results were found as the concentration of full-length initiator was increased from 25 pmol/µL to 100 pmol/µL (fixed volume at 10 µL and total input 250 pmol to 1000 pmol). No remaining full length initiator oligonucleotide or erroneous side products were observed in any of the Endonuclease V test cases. The uncropped scan of this gel is supplied at the end of the document.

Variable Sequence – 5137.98 Da
5’-PO-ACAAGCACACUAAAUUA-3’

Dephosphorylated Variable Sequence – 5057.16 Da
5’-OH-ACAAGCACACUAAAUUA-3

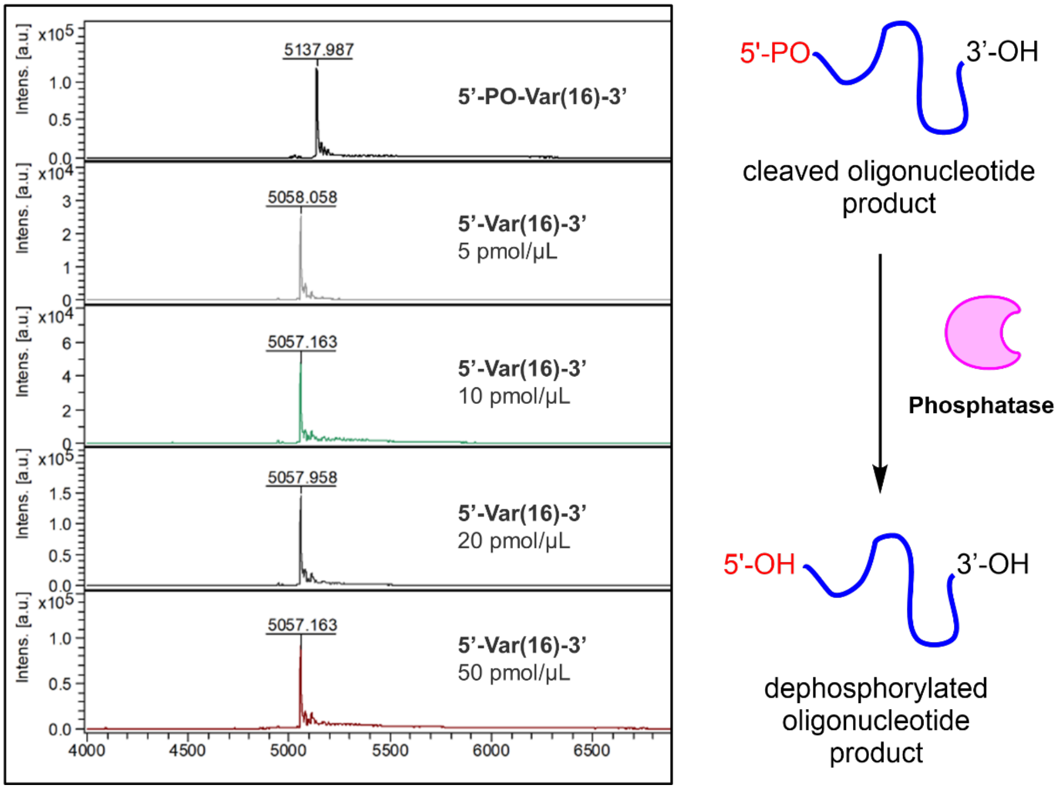

Fig. S16: A general scheme for the enzymatic removal of a 5’-phosphate (PO) group with a phosphatase from an oligonucleotide product that was cleaved from its initiator used in template-independent enzymatic synthesis. MALDI-TOF mass spectrometry was used to assess the dephosphorylation activity of Antarctic Phosphatase (NEB) in the presence of an RNA oligonucleotide sequence comprised of variable bases bearing a 5’-phosphate under standard reaction conditions. The expected dephosphorylated product was observed in each test case in which the total 5’-phosphate oligonucleotide concentration was increased from 5 pmol/µL to 50 pmol/µL.

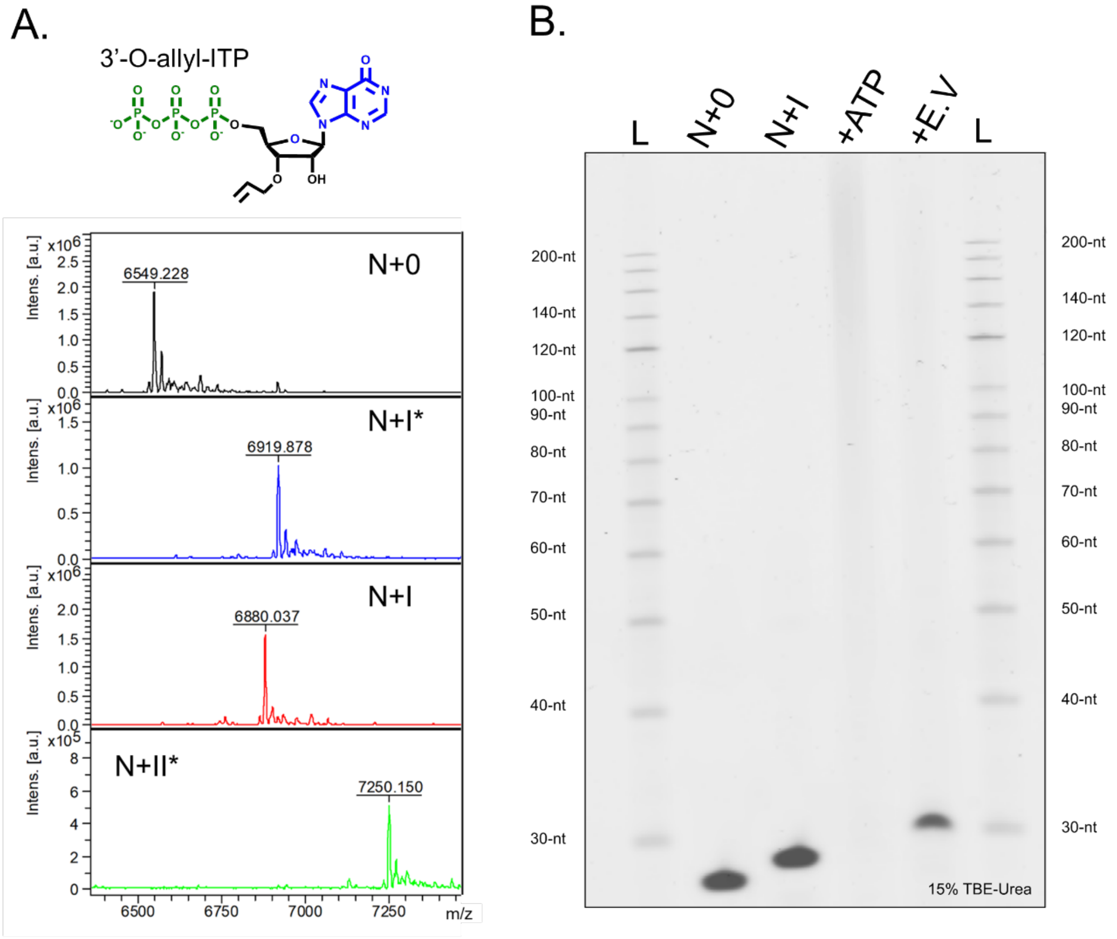

N = 5'-Am-C12/UUU/Cy5/UUUUUUUUUUUUUUUU ~ 19-nt

Fig. S17: (A) MALDI-TOF analysis of 3'-O- allyl ether ITP reversible terminator during a single cycle of enzymatic synthesis: initial incorporation (N+I*), deblocking (N+I), and generation of an N+2* single base transition product. **(B)** Following uncontrolled polymerization with unblocked ATP, Endonuclease V was used to cleave initiator oligonucleotide from the resultant homopolymer sequence. Denaturing gel electrophoresis shows the unextended initiator as a negative control (N+0), initiator extended and deblocked N+I, which was subsequently used to generate the long (+ATP) homopolymer sequence that was digested with Endonuclease V to restore initiator oligonucleotide with sequence N+I-A (+E.V). A 200-nt ssDNA ladder to measure the overall length of uncontrolled polymerization. The gel was imaged with the Cy3 and Cy5 channels consecutively, and these images were then overlayed This experiment was conducted once. The uncropped scan of this gel is supplied at the end of the document.

A. B.

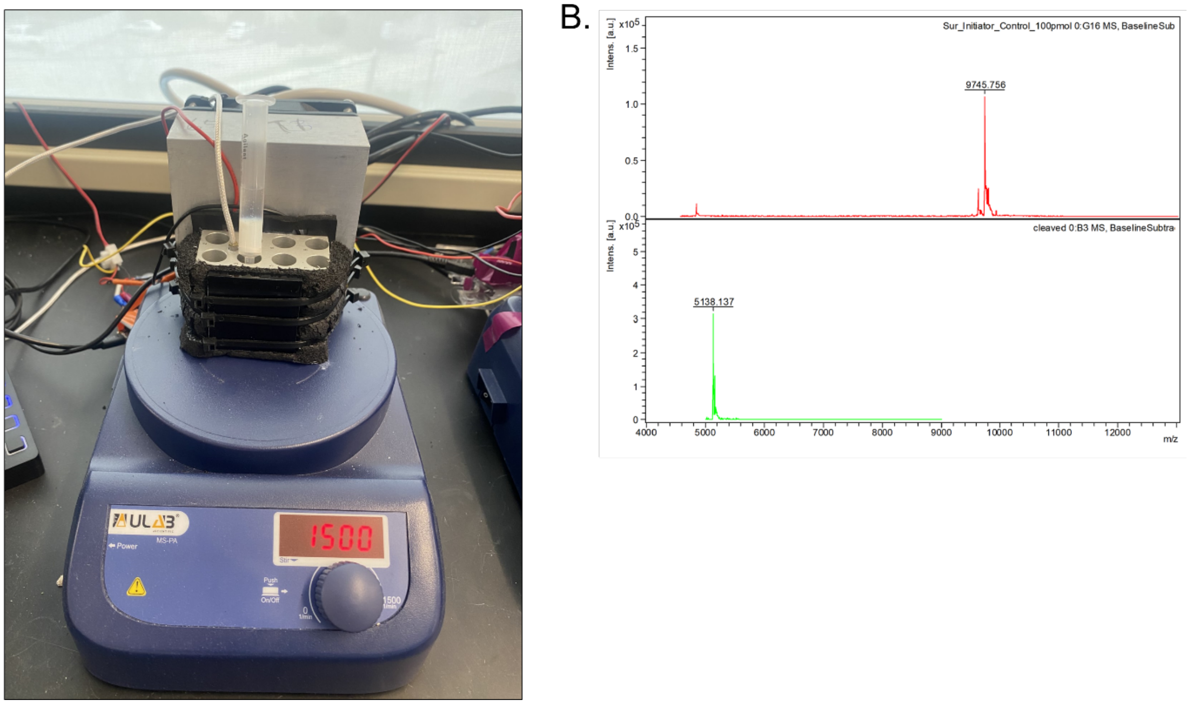

Fig. S18: **(A)** Picture of a stir reactor, which is comprised of a heat block controlled by a Pelletier unit and placed on top of a magnetic stir place. Solid support is placed within a 3 mL solid phase extraction column with a small magnetic flea and the appropriate reaction master mix for either extension, deblocking or Endonuclease V cleavage is added directly. Custom software controls the heat block temperature, which is regulated by a wire probe inserted into a drilled-out hole in the block. **(B)** MALDI-TOF analysis of Endonuclease V-mediated cleavage of the full surface initiator oligonucleotide.

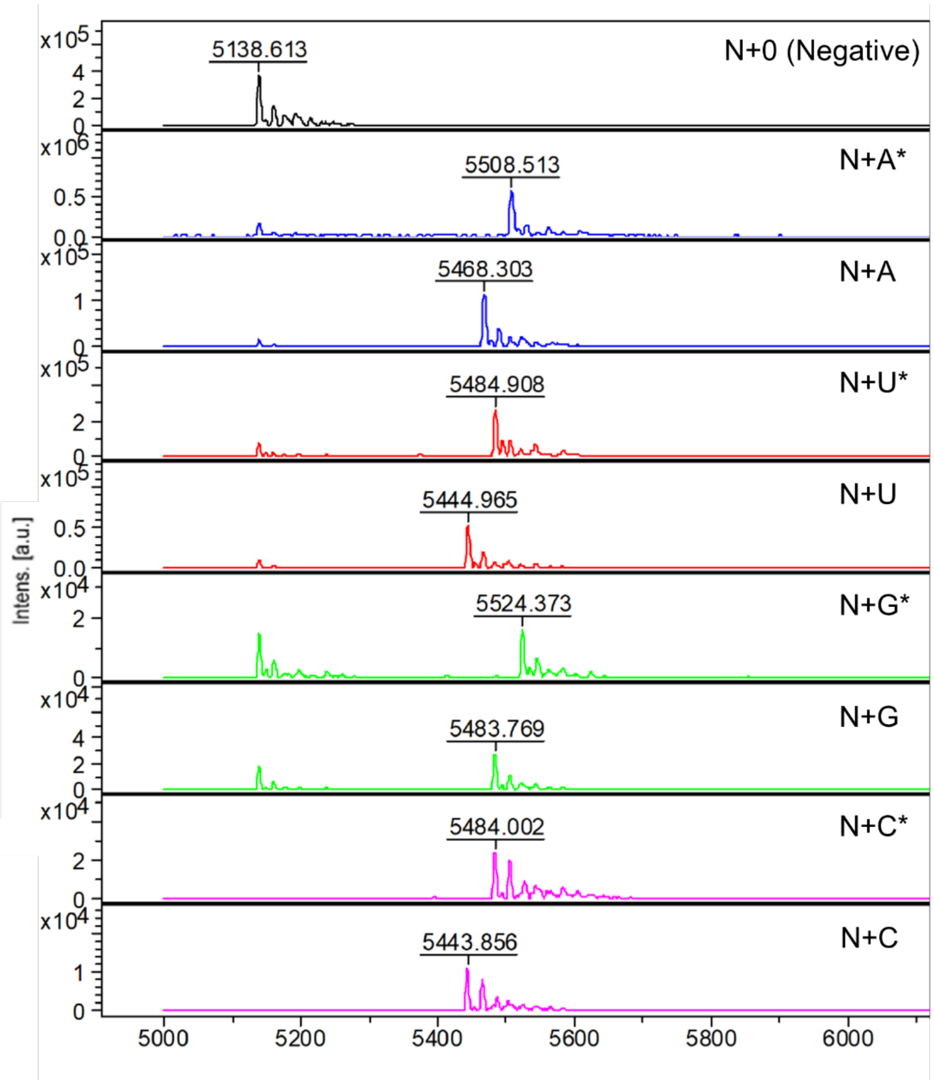

Fig. S19: MALDI-TOF analysis of oligonucleotide products cleaved from the CPG solid support via Endonuclease V after a single cycle of enzymatic extension and deblocking with the full 3'-O-allyl ether NTP set. The negative control reaction contained all extension reaction components with the exception of NTP building block and did not undergo deblocking.

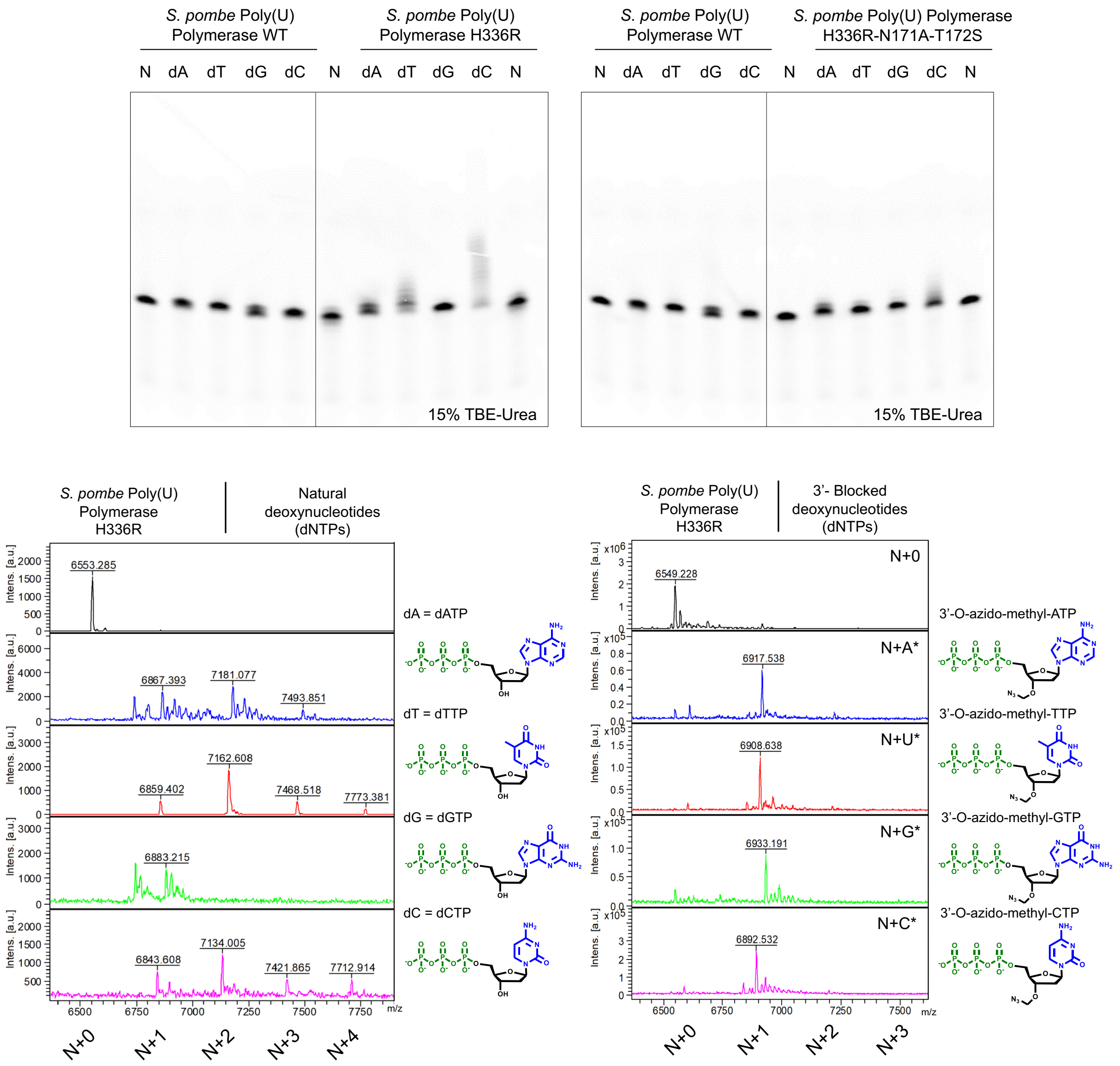

Fig. S20A: A comparison of uncontrolled polymerization catalyzed by wild-type *S. pombe* Poly(U) Polymerase and two PUP mutant variants (H336R & H336R-N171A-T172S) using the natural unblocked deoxynucleotide (dNTPs) by denaturing gel electrophoresis. Control reactions (N) contained all components except for nucleotide. Because incorporation was limited to just a few extensions, MALDI-TOF spectra was also obtained and is compared to the incorporation of a set 3'-O-azido-methyl ether dNTP reversible terminators. This direct comparison was performed once but represents a compilation of several independent experimental repeats with similar results. The uncropped scan of this gel is supplied at the end of the document.

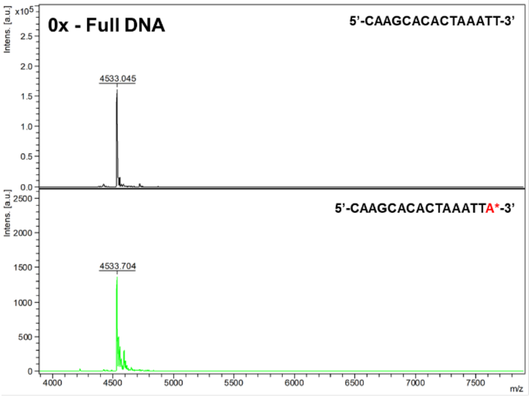

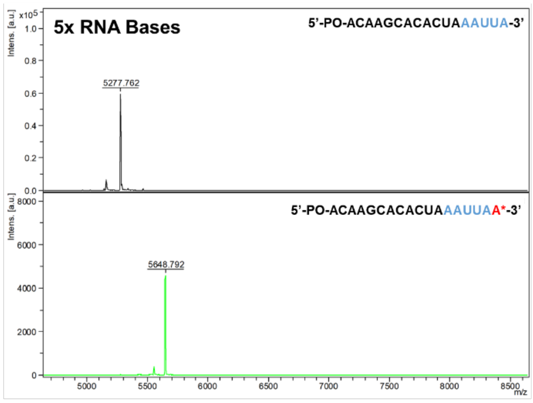

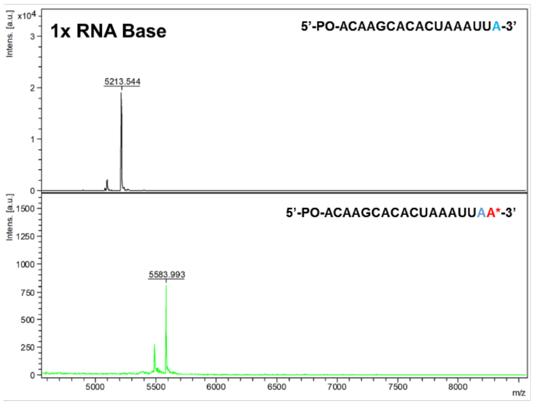

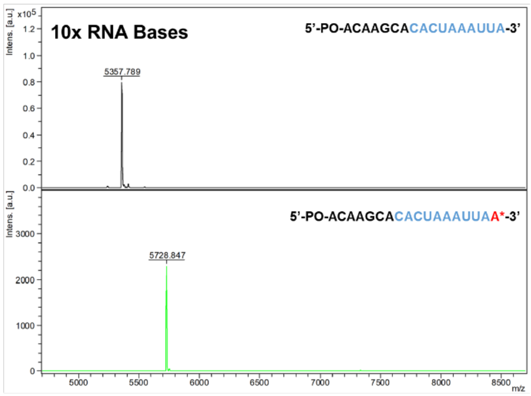

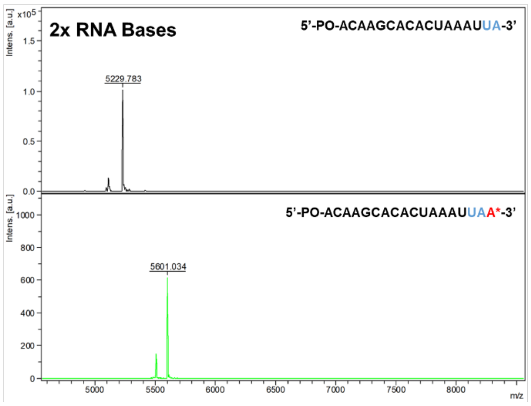

Observed MALDI-TOF Masses

# RNA Bases at 3'-Terminus	N+0 (m/z)	N+A* (m/z)
0x (Full DNA)	4533.04	4533.70*
1x RNA	5213.54	5583.99
2x RNA	5229.78	5601.03
5x RNA	5277.76	5648.79
10x RNA	5357.78	5728.84

* Indicates no conversion to the final product

Fig. S20B: An evaluation of Poly(U) Polymerase mutant variant H336R extension activity under standard reaction conditions with a 3'-O- allyl ether ATP reversible terminator in the presence of initiator oligonucleotides with a decreasing number of RNA (2'-OH) bases at the 3'-terminus using MALDI-TOF mass spectrometry. For each sequence, DNA and RNA bases are indicated in black

and blue, respectively. Mass spectra of initiators are shown before (top, black) and after (bottom, green) enzymatic extension.

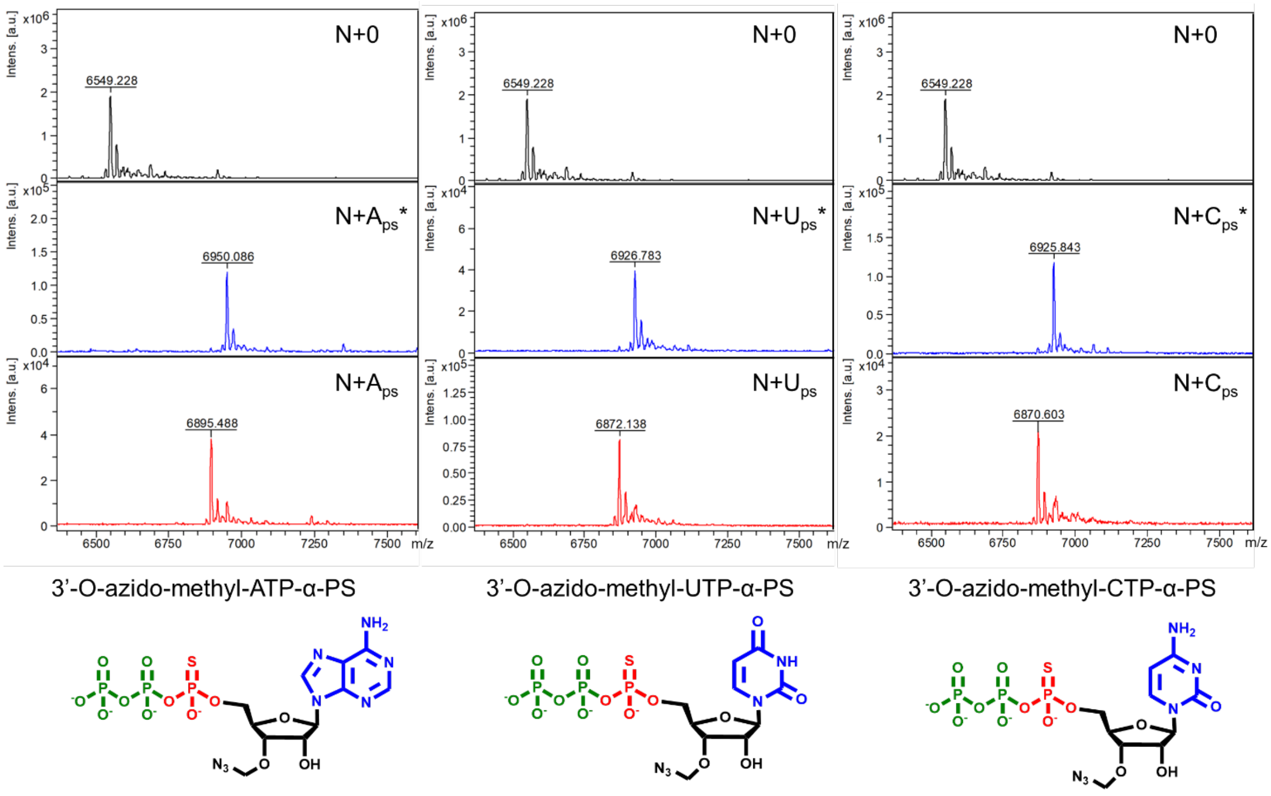

Fig. S21: An assessment of controlled, enzymatic oligonucleotide synthesis using a partial set of α-phosphorothioate (α-PS) modified 3'-O-azido-methy ether reversible terminator NTPs (A, U, C) with PUP mutant variant H336R. Extension reactions were carried out using standard reaction conditions using a Cy5 labeled 19-nt initiator oligonucleotide. MALDI-TOF mass spectrometry was used to assess the efficiency of initiator conversion to the **N+1*** product and azido methyl TCEP deblocking to **N+1**.

Figure	Primary Product	Calc. (m/z)	Obs. (m/z)	M.W. (Da)
**2B**	N	6548.0	6549.2	6551.2
	N+A*	6917.1	6919.0	6920.4
	N+A	6877.1	6879.7	6880.4
	N+U*	6894.1	6896.1	6897.3
	N+U	6854.1	6856.1	6857.3
	N+G*	6933.1	6935.3	6936.4
	N+G	6893.1	6895.3	6896.4
	N+C*	6893.1	6895.3	6896.4
	N+C	6853.1	6855.5	6856.4
**Figure**	**Primary Product**	**Calc. (m/z)**	**Obs. (m/z)**	**M.W. (Da)**
**2C**	N	6548.0	6549.2	6551.2
	N+A*	6917.1	6919.0	6920.4
	N+AA*	7246.2	7246.9	7249.6
	N+AU*	7223.1	7223.9	7226.5
	N+AC*	7222.2	7227.8	7225.6
	N+AG*	7262.2	7262.8	7265.6
	N+U*	6894.1	6896.1	6897.3
	N+UA*	7223.1	7223.9	7226.5
	N+UU*	7200.1	7202.2	7203.5
	N+UC*	7199.1	7204.4	7202.5
	N+UG*	7239.1	7238.8	7242.5
	N+C*	6893.1	6895.3	6896.4
	N+CA*	7222.2	7244.5	7225.6
	N+CU*	7199.1	7204.3	7202.5
	N+CC*	7198.1	7202.7	7201.5
	N+CG*	7238.2	7245.4	7241.6
	N+G*	6933.1	6935.3	6936.4
	N+GA*	7262.2	7263.8	7265.6
	N+GU*	7239.1	7240.2	7242.5
	N+GC*	7238.2	7243.3	7241.6
	N+GG*	7278.2	7284.5	7281.6
**Figure**	**Primary Product**	**Calc. (m/z)**	**Obs. (m/z)**	**M.W. (Da)**
**3B**	N	6548.0	6549.2	6551.2
	N+U*	6894.1	6901.2	6897.3
	N+U	6854.1	6860.2	6857.3
	N+UU*	7200.1	7206.5	7203.5
	N+UU	7160.1	7166.6	7163.5
	N+UUU*	7506.2	7512.2	7509.7

	N+UUU	7466.1	7472.3	7469.7
	N+UUUC*	7811.2	7816.8	7814.9
	N+UUUC	7771.2	7776.3	7774.9
	N+UUUCG*	8156.2	8164.4	8160.1
**Figure**	**Primary Product**	**Calc. (m/z)**	**Obs. (m/z)**	**M.W. (Da)**
**3F**	N+ACACCUUAAC*	9736.5	9738.9	9741.1
	N+ACACCUUAAC(+A)*	10065.53	10068.0	10070.3

Table S1: Overview of oligonucleotide products generated by synthesis activities described in Fig. 2B, 2C & Fig. 3B, 3F using MALDI-TOF mass spectrometry. The calculated mass-to-charge ratio value (m/z) for each product was determined with the assistance of the ChemDraw software and is compared to the observed (m/z) value. The molecular weight (M.W.) of each product in Dalton (Da) is also provided and was determined using the IDT Oligoanalyzer tool. Observed impurities are marked in red.

Figure	Primary Product	Calc. (m/z)	Obs. (m/z)	M.W. (Da)
4A	N+Am*	6931.1	6932.0	6934.4
	N+Am	6891.1	6892.9	6894.4
	N+AmAm*	7274.2	7275.9	7277.6
	N+Um*	6908.1	6909.8	6911.4
	N+Um	6867.1	6869.7	6871.4
	N+UmUm*	7227.2	7250.1	7131.6
	N+Gm*	6947.1	6949.4	6950.4
	N+Gm	6907.1	6909.8	6910.4
	N+GmGm*	7306.2	7308.9	7309.6
	N+Cm*	6907.1	6909.9	6910.4
	N+Cm	6867.1	6868.7	6870.4
	N+CmCm*	7226.2	7252.2	7229.6
	N+Af*	6919.1	6921.2	6922.4
	N+Af	6879.1	6880.4	6882.4
	N+AfAf*	7250.2	7253.5	7253.6
	N+Uf*	6896.1	6897.8	6899.3
	N+Uf	6856.1	6857.9	6859.3
	N+UfUf*	7204.1	7206.1	7207.5
	N+Gf*	6935.1	6936.9	6938.4
	N+Gf	6895.1	6900.5	6898.4
	N+GfGf*	7282.1	7291.4	7295.6
	N+Cf*	6895.1	6896.3	6898.3
	N+Cf	6855.1	6860.8	6858.3
	N+CfCf*	7202.1	7207.4	7205.5
	N+A(ps)*	6933.1	6935.0	6936.4
	N+A(ps)	6893.1	6612.1 / 6879.1	6896.4
	N+A(ps)A(ps)*	7278.1	n/a	7281.7
	N+U(ps)*	6910.1	6911.8	6913.4
	N+U(ps)	6870.0	6611.7 / 6855.6	6873.4
	N+U(ps)U(ps)*	7232.1	n/a	7235.6
	N+G(ps)*	6949.1	6950.8	6952.4
	N+G(ps)	6909.1	6611.9 / 6895.8	6912.4
	N+G(ps)G(ps)*	7310.1	n/a	7313.7
	N+C(ps)*	6909.1	6910.9	6902.4
	N+C(ps)	6869.1	6611.9 / 6853.5	6872.4
	N+C(ps)C(ps)*	7230.1	n/a	7233.7
**Figure**	**Primary Product**	**Calc. (m/z)**	**Obs. (m/z)**	**M.W. (Da)**
**4B**	N+Ap	6915.1	6916.2	6918.2

	N+Up	6892.1	6893.3	6895.1
	N+Cp	6891.1	6891.4	6894.2
	N+Gp	6931.1	6932.3	6934.2
**Figure**	**Primary Product**	**Calc. (m/z)**	**Obs. (m/z)**	**M.W. (Da)**
**4D**	N	6548.0	6549.2	6551.2
	N+Af*	6919.1	6924.3	6922.4
	N+AfAf*	7250.1	7256.6	7253.6
	N+AfAfCm*	7569.2	7577.4	7572.8
	N+AfAfCmCm*	7888.3	7894.2	7892.0
	N+AfAfCmCmUf*	8196.3	8202.2	8200.1
	N+AfAfCmCmUfUf*	8504.3	8508.7	8508.3
	N+AfAfCmCmUfUfUf*	8812.3	8818.1	8816.5
	N+AfAfCmCmUfUfUfCm*	9131.4	9135.9	9135.7
	N+AfAfCmCmUfUfUfCmUf*	9439.4	9443.8	9443.8
	N+AfAfCmCmUfUfUfCmUfAp	9766.5	9772.0	9770.8

Table S2: Overview of oligonucleotide products generated by synthesis activities described in Fig. 4A, 4B, & 4D using MALDI-TOF mass spectrometry. The calculated mass-to-charge ratio value (m/z) for each product was determined with the assistance of the ChemDraw software and is compared to the observed (m/z) value. The molecular weight (M.W.) of each product in Dalton (Da) is also provided and was determined using the IDT Oligoanalyzer tool. Observed impurities are marked in red and products that were not obtained are marked in yellow.

Fig.	Product	Sequence	Calc. (m/z)	Obs. (m/z)	M.W. (Da)
5C,D	Oligo 1 - Primary Product (N+5)	N+AAUUC*	6747.9	6749.8	6752.1
	Oligo 1 - Minor Product A (N+4)	N+AAUU	6402.9	6456.9	6406.9
	Oligo 1 - Minor Product B (N+6)	N+AAUUC*(+U)	7054.0	7056.8	7058.0
	Oligo 2 - Primary Product (N+5)	N+AACUG*	6787.0	6789.9	6791.0
	Oligo 2 - Minor Product A (N+4)	N+AACU	6401.9	6405.0	6405.9
	Oligo 3 - Primary Product (N+5)	N+CCAAU*	6747.0	6747.8	6751.0
	Oligo 3 - Minor Product A (N+4)	N+CCAA	6400.9	6419.8	6404.9
	Oligo 3 - Minor Product B (N+6)	N+CCAAU*(+A)	7076.0	7077.8	7080.3
	Oligo 4 - Primary Product (N+5)	N+UfAUfAUf*	6754.9	6757.7	6759.0
	Oligo 4 - Minor Product A (N+4)	N+UfAUfA	6406.9	6428.8	6410.9
	Oligo 4 - Minor Product B (N+6)	N+UfAUfAUf*(+A)	7084.0	7086.7	7124.2
	Oligo 5 - Primary Product (N+5)	N+UfUmUfUmUf*	6736.9	6739.6	6741.0
	Oligo 5 - Minor Product A (N+6)	N+UfUmUfUm	6388.8	6431.8	6392.8

Table S3: Overview of oligonucleotide products generated by synthesis activities described in Fig. 5C & 5D using MALDI-TOF mass spectrometry. The calculated mass-to-charge ratio value (m/z) for each product was determined with the assistance of the ChemDraw software and is compared to the observed (m/z) value. The molecular weight (M.W.) of each product in Dalton (Da) is also provided and was determined using the IDT Oligoanalyzer tool. Observed impurities are marked in red.

SEQ1: *S. pombe* Poly(U) Polymerase WT

MNISSAQFIPGVHTVEEIEAEIHKNLHISKSCSYQKVPNSHKEFTKFCYEVYNEIKISDKEF
KEKRAALDTLRLCLKRISPDAELVAFGSLESGLALKNSDMDLCVLMDSRVQSDTIALQF
YEELIAEGFEGKFLQRARIPIIKLTSDTKNGFGASFQCDIGFNNRLAIHNTLLLSSYTKLDA
RLKPMVLLVKHWAKRKQINSPYFGTLSSYGYVLMVLYYLIHVIKPPVFPNLLLSPLKQE
KIVDGFDVGFDDKLEDIPPSQNYSSLGSLLHGFFRFYAYKFEPREKVVTFRRPDGYLTKQ
EKGWTSATEHTGSADQIIKDRYILAIEDPFEISHNVGRTVSSSGLYRIRGEFMAASRLLNS
RSYPIPYDSLFEEAPIPPRRQKKTDEQSNKKLLNETDGDNSE

SEQ2: *S. pombe* Poly(U) Polymerase H336R

MNISSAQFIPGVHTVEEIEAEIHKNLHISKSCSYQKVPNSHKEFTKFCYEVYNEIKISDKEF
KEKRAALDTLRLCLKRISPDAELVAFGSLESGLALKNSDMDLCVLMDSRVQSDTIALQF
YEELIAEGFEGKFLQRARIPIIKLTSDTKNGFGASFQCDIGFNNRLAIHNTLLLSSYTKLDA
RLKPMVLLVKHWAKRKQINSPYFGTLSSYGYVLMVLYYLIHVIKPPVFPNLLLSPLKQE
KIVDGFDVGFDDKLEDIPPSQNYSSLGSLLHGFFRFYAYKFEPREKVVTFRRPDGYLTKQ
EKGWTSATEHTGSADQIIKDRYILAIEDPFEIS**R**NVGRTVSSSGLYRIRGEFMAASRLLNS
RSYPIPYDSLFEEAPIPPRRQKKTDEQSNKKLLNETDGDNSE

SEQ3: *S. pombe* Poly(U) Polymerase H336R-N171A-T172S

MNISSAQFIPGVHTVEEIEAEIHKNLHISKSCSYQKVPNSHKEFTKFCYEVYNEIKISDKEF
KEKRAALDTLRLCLKRISPDAELVAFGSLESGLALKNSDMDLCVLMDSRVQSDTIALQF
YEELIAEGFEGKFLQRARIPIIKLTSDTKNGFGASFQCDIGFNNRLAIH**AS**LLLSSYTKLDA
RLKPMVLLVKHWAKRKQINSPYFGTLSSYGYVLMVLYYLIHVIKPPVFPNLLLSPLKQE
KIVDGFDVGFDDKLEDIPPSQNYSSLGSLLHGFFRFYAYKFEPREKVVTFRRPDGYLTKQ

EKGWTSATEHTGSADQIIKDRYILAIEDPFEIS**R**NVGRTVSSSGLYRIRGEFMAASRLLNS
RSYPIPYDSLFEEAPIPPRRQKKTDEQSNKKLLNETDGDNSE

SEQ4: ***E. coli*** **Endonuclease V WT**

MDLASLRAQQIELASSVIREDRLDKDPPDLIAGADVGFEQGGEVTRAAMVLLKYPSLEL
VEYKVARIATTMPYIPGFLSFREYPALLAAWEMLSQKPDLVFVDGHGISHPRRLGVASH
FGLLVDVPTIGVAKKRLCGKFEPLSSEPGALAPLMDKGEQLAWVWRSKARCNPLFIATG
HRVSVDSALAWVQRCMKGYRLPEPTRWADAVASERPAFVRYTANQP

SEQ5: ***E. coli*** **Endonuclease V WT – N-Term. MBP Fusion**

MDLASLRAQQIELASSVIREDRLDKDPPDLIAGADVGFEQGGEVTRAAMVLLKYPSLEL
VEYKVARIATTMPYIPGFLSFREYPALLAAWEMLSQKPDLVFVDGHGISHPRRLGVASH
FGLLVDVPTIGVAKKRLCGKFEPLSSEPGALAPLMDKGEQLAWVWRSKARCNPLFIATG
HRVSVDSALAWVQRCMKGYRLPEPTRWADAVASERPAFVRYTANQPMKIEEGKLVIW
INGDKGYNGLAEVGKKFEKDTGIKVTVEHPDKLEEKFPQVAATGDGPDIIFWAHDRFGG
YAQSGLLAEITPDKAFQDKLYPFTWDAVRYNGKLIAYPIAVEALSLIYNKDLLPNPPKT
WEEIPALDKELKAKGKSALMFNLQEPYFTWPLIAADGGYAFKYENGKYDIKDVGVDNA
GAKAGLTFLVDLIKNKHMNADTDYSIAEAAFNKGETAMTINGPWAWSNIDTSKVNYGV
TVLPTFKGQPSKPFVGVLSAGINAASPNKELAKEFLENYLLTDEGLEAVNKDKPLGAVA
LKSYEEELAKDPRIAATMENAQKGEIMPNIPQMSAFWYAVRTAVINAASGRQTVT

3’-O- allyl ether ATP – Proton NMR

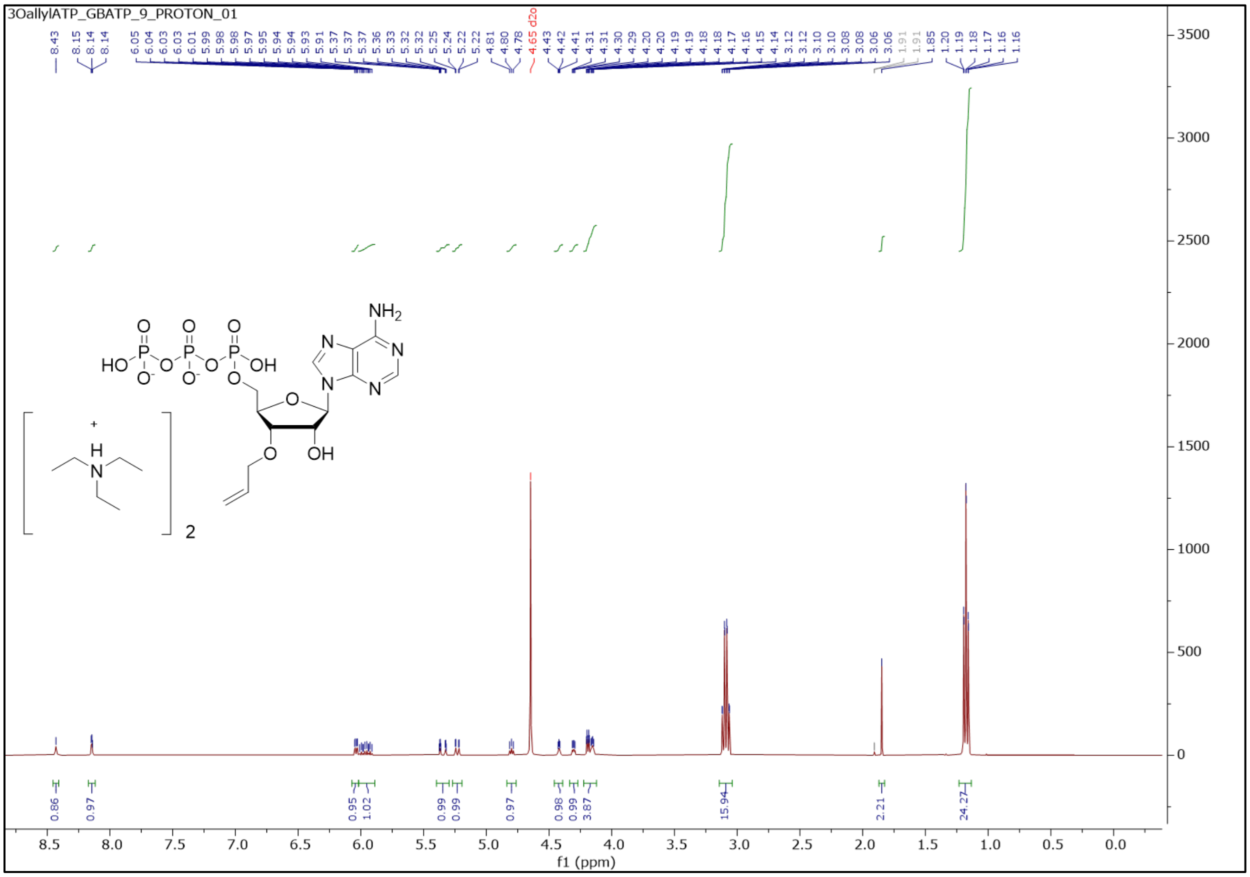

3'-O- allyl ether ATP – Phosphorous NMR

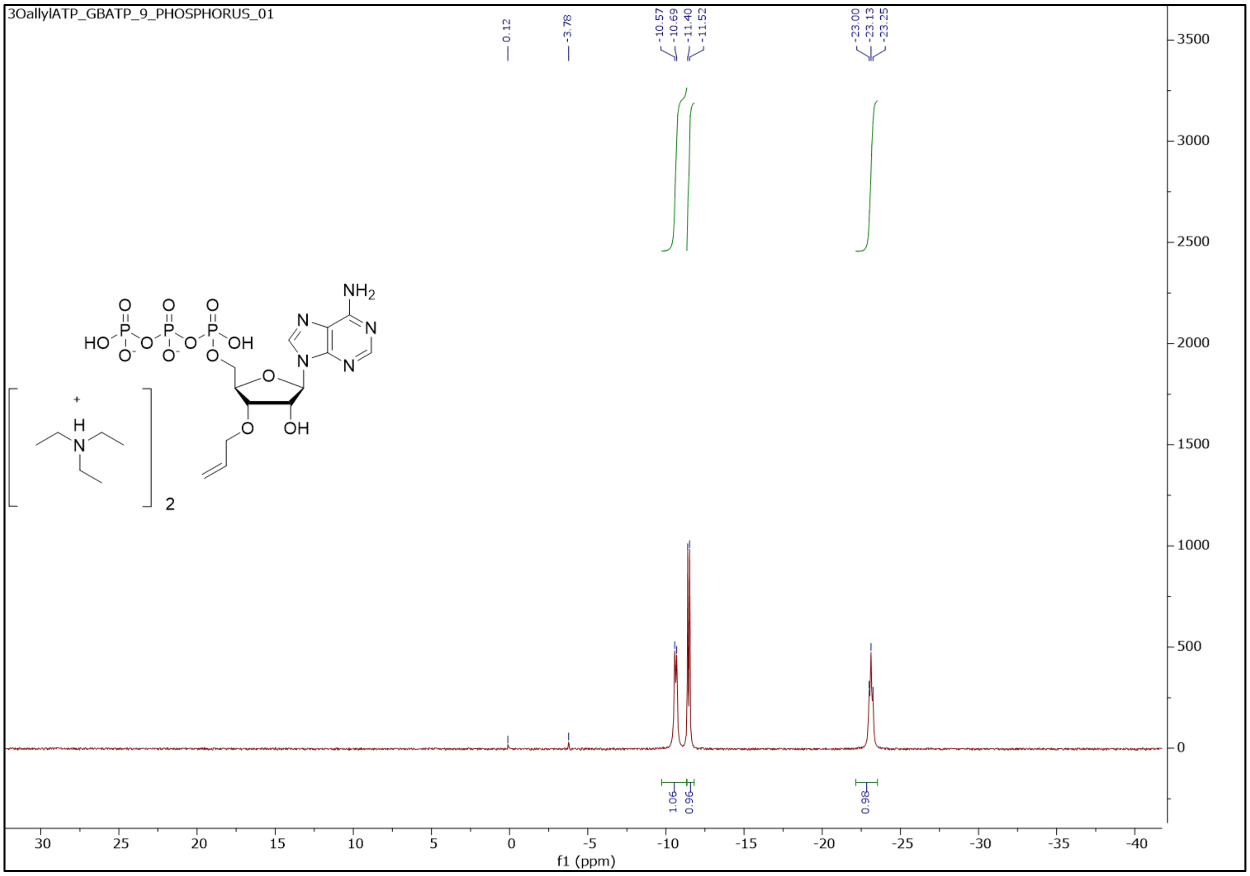

3’-O- allyl ether UTP – Proton NMR

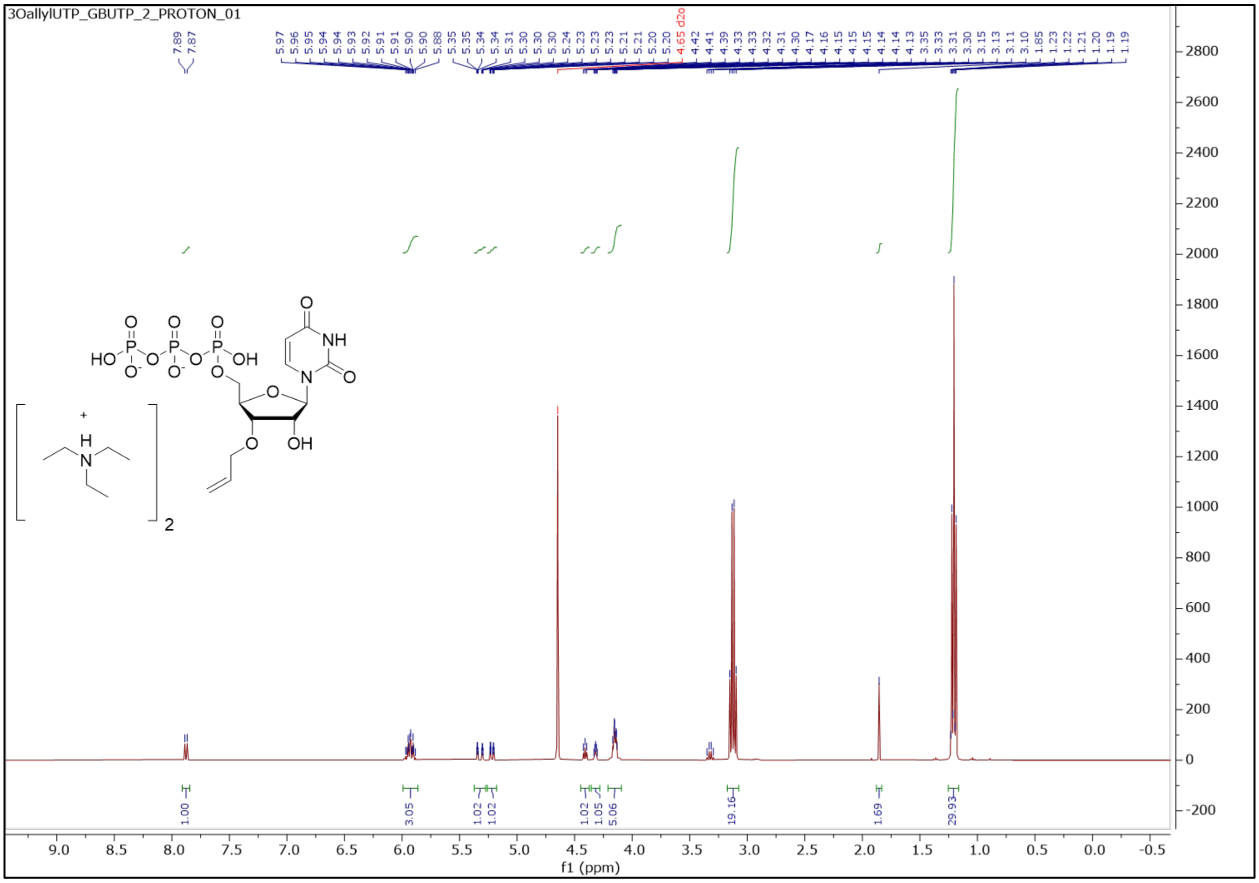

3'-O- allyl ether UTP – Phosphorous NMR

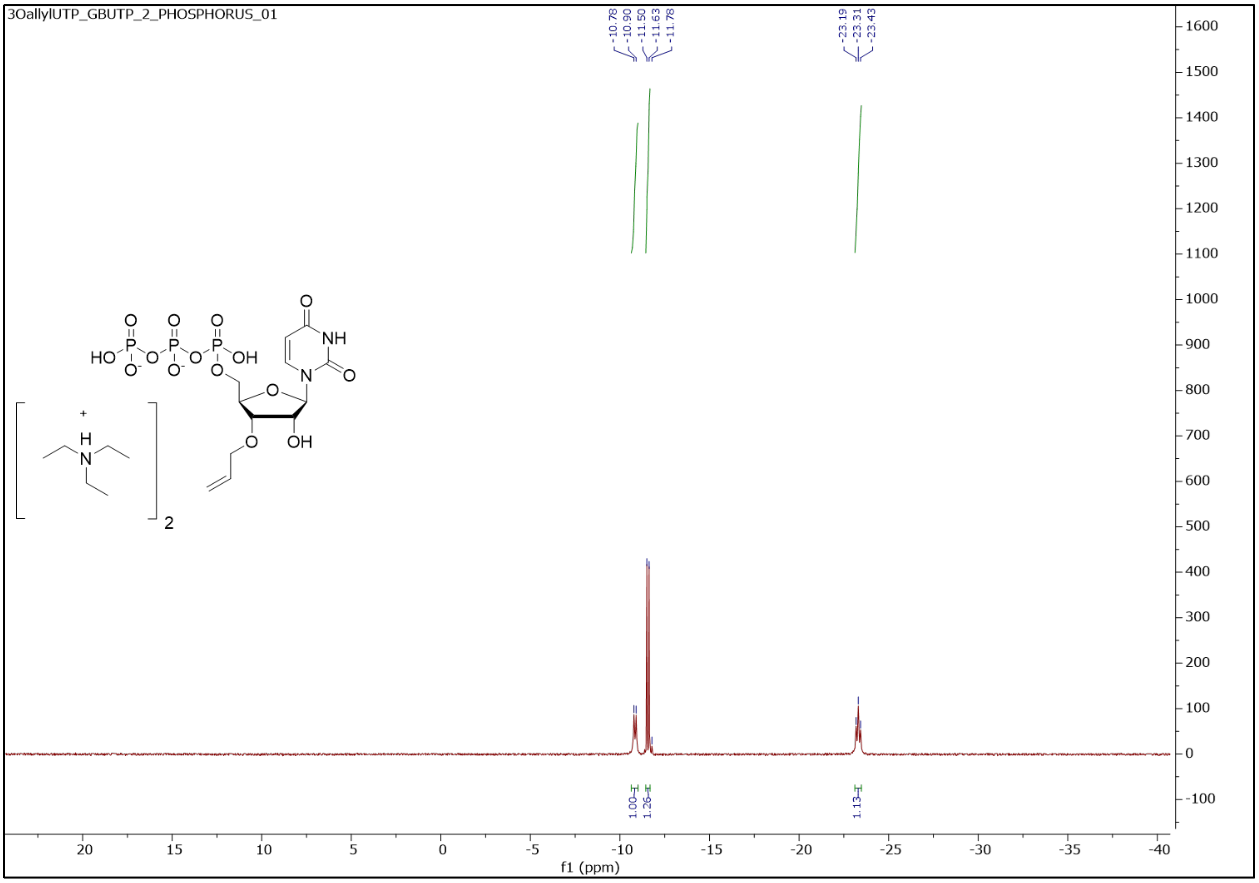

3’-O- allyl ether GTP – Proton NMR

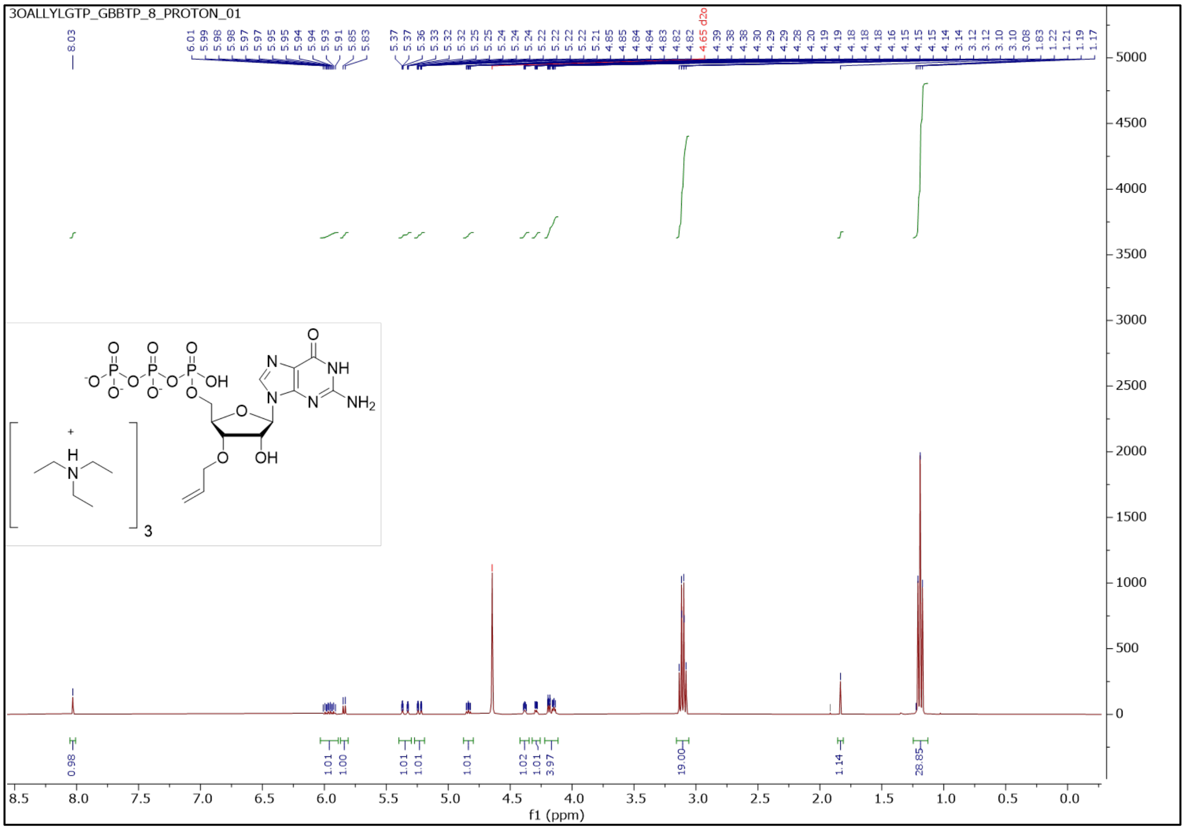

3'-O- allyl ether GTP – Phosphorous NMR

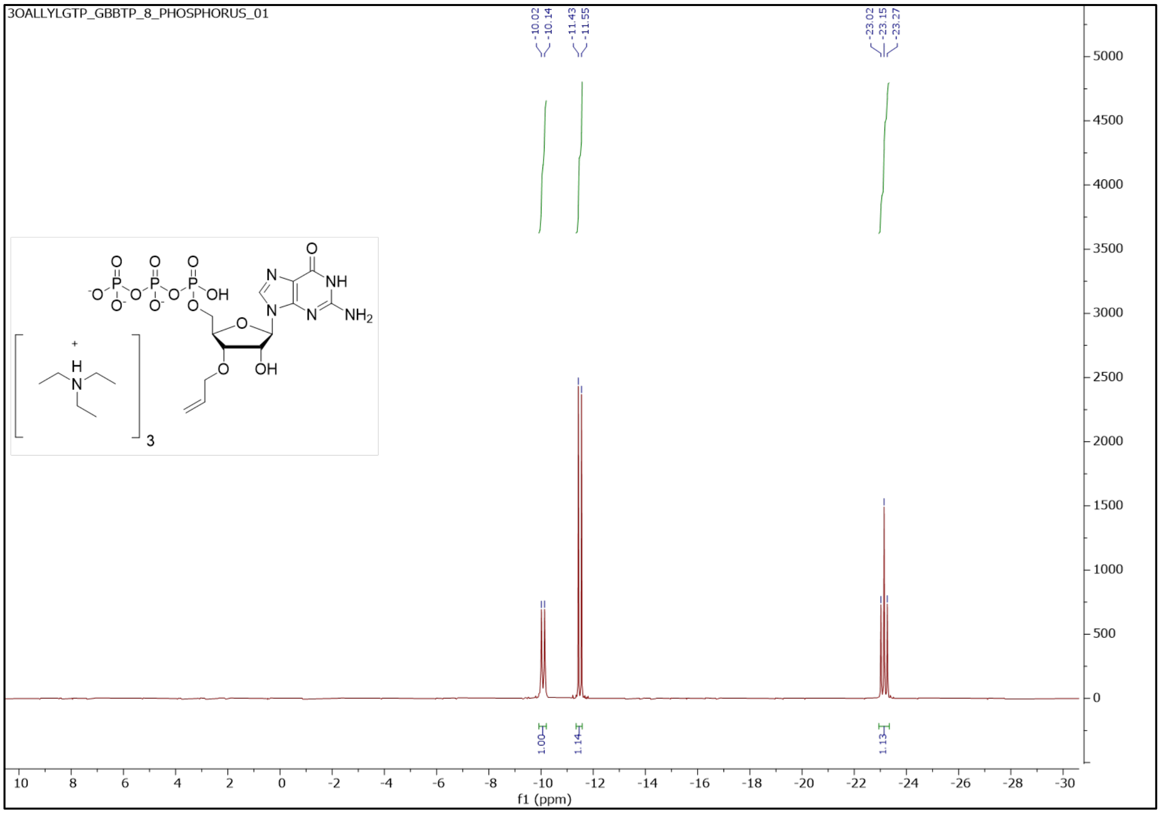

3’-O- allyl ether CTP – Proton NMR

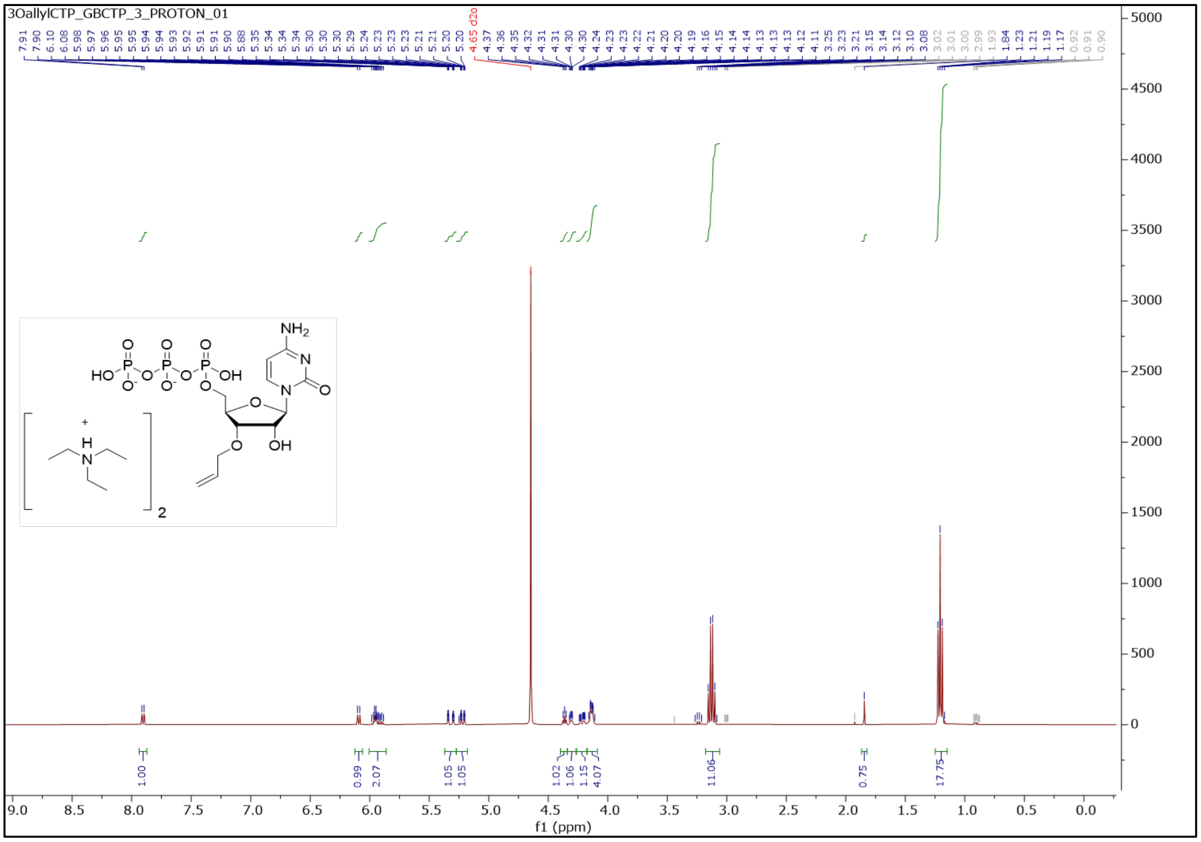

3’-O- allyl ether CTP – Phosphorous NMR

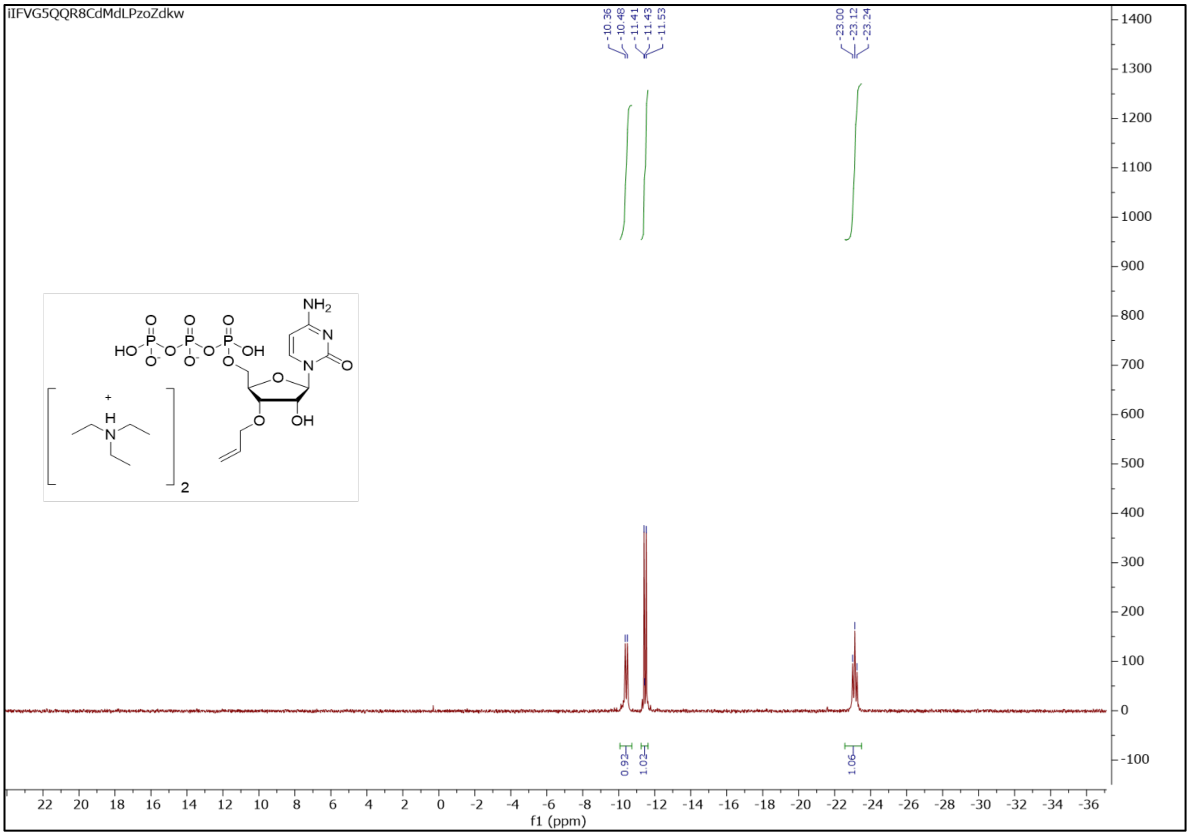

Unmodified Gel for Fig. S1

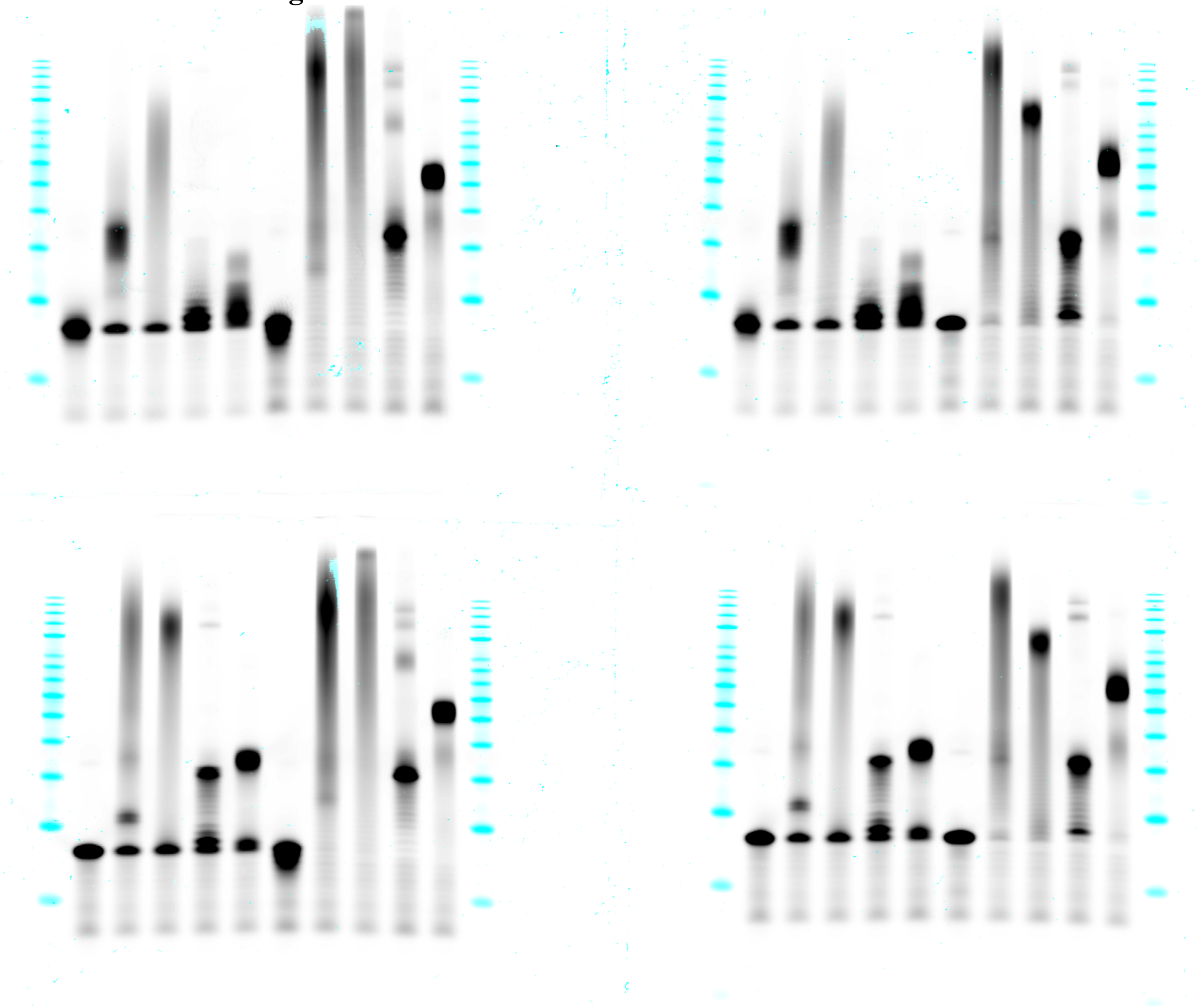

Unmodified Gel for Fig. S2C (Cy3 Channel)

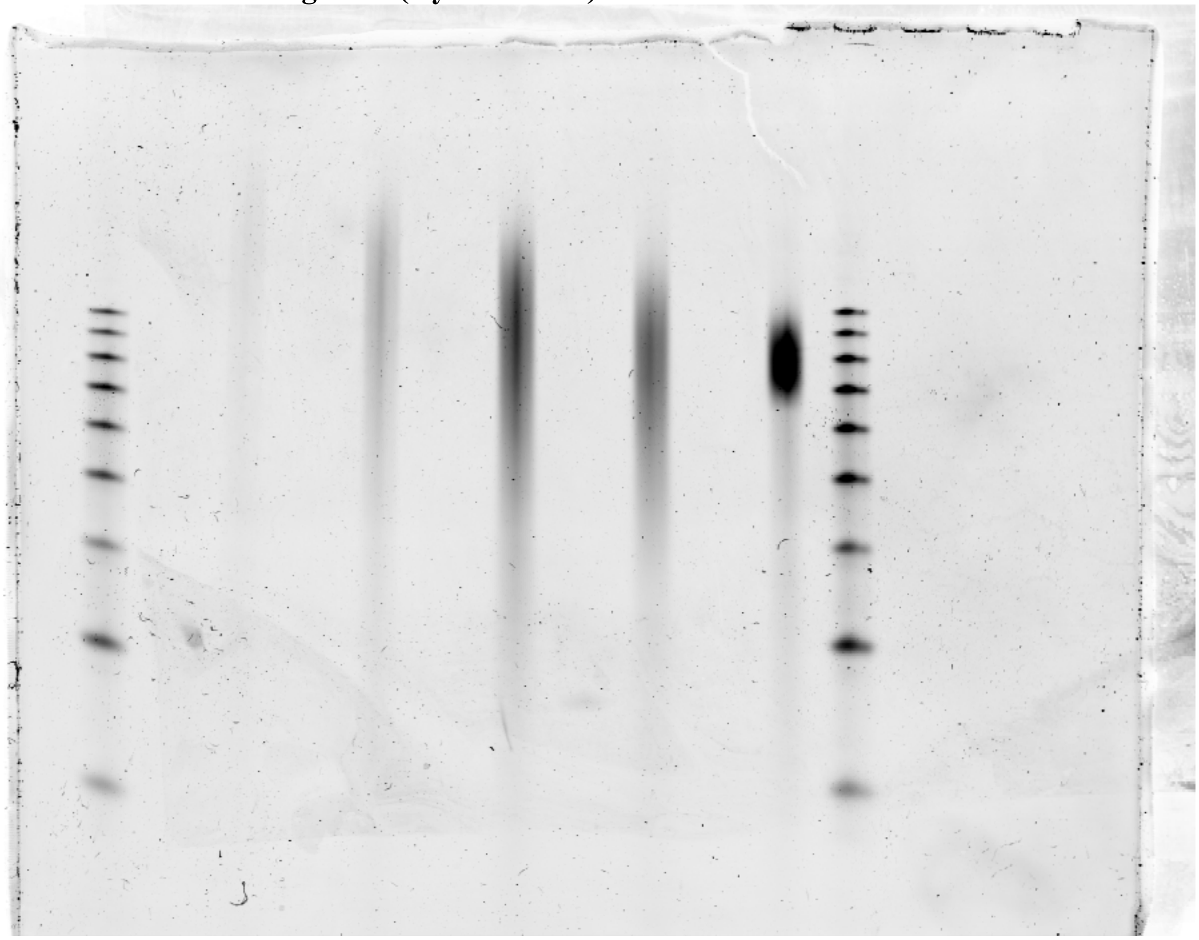

Unmodified Gel for Fig. S2C (Cy5 Channel)

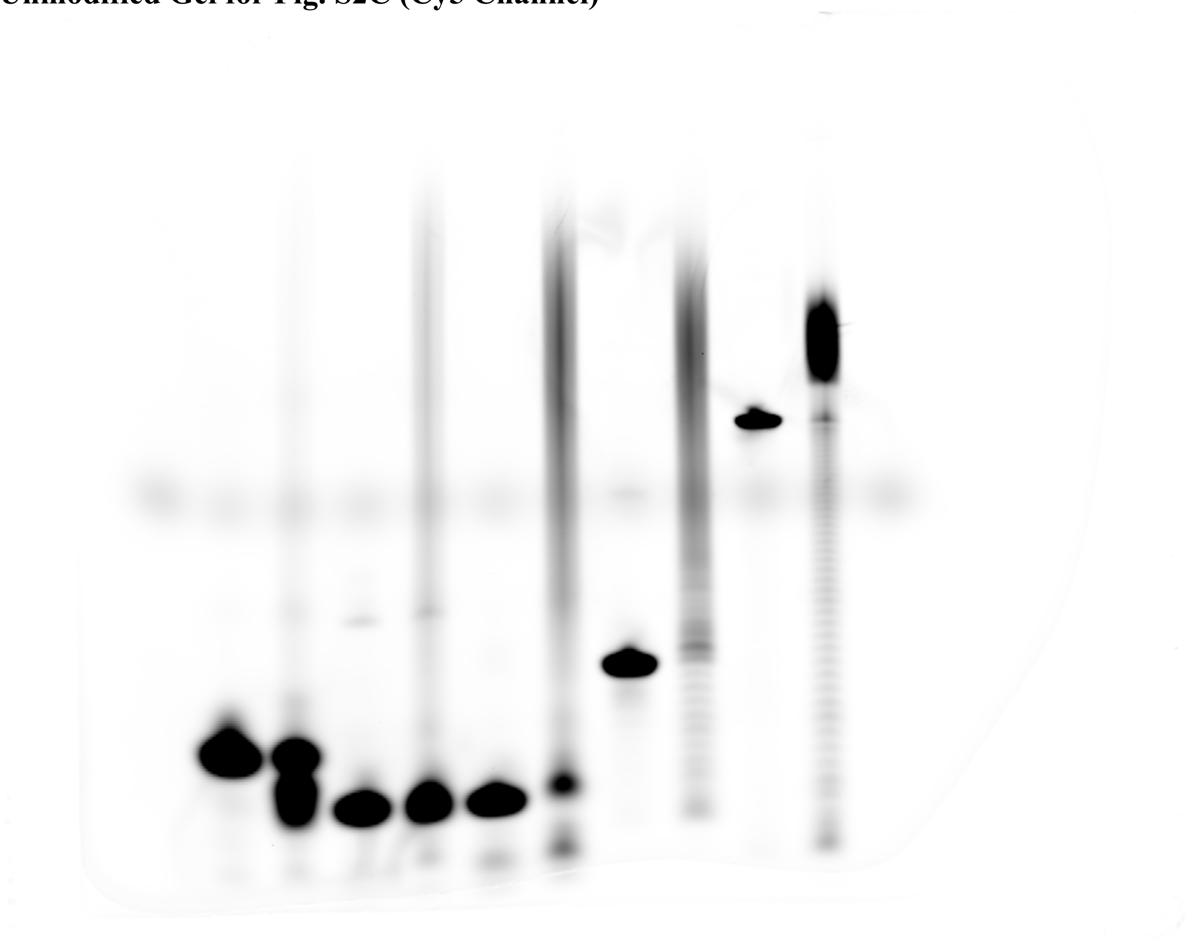

Unmodified Gel for Fig. S3B

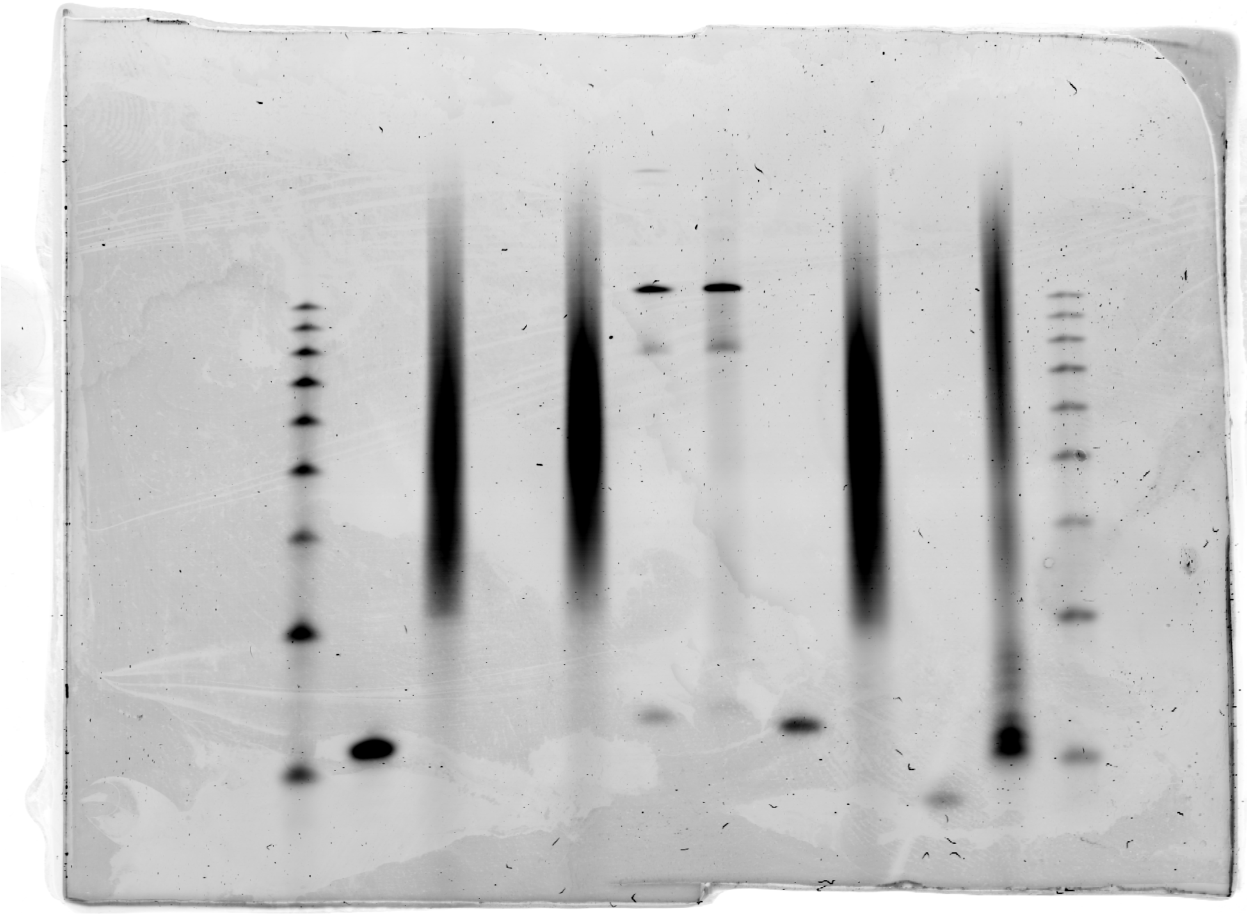

Unmodified Gel for Fig. S6

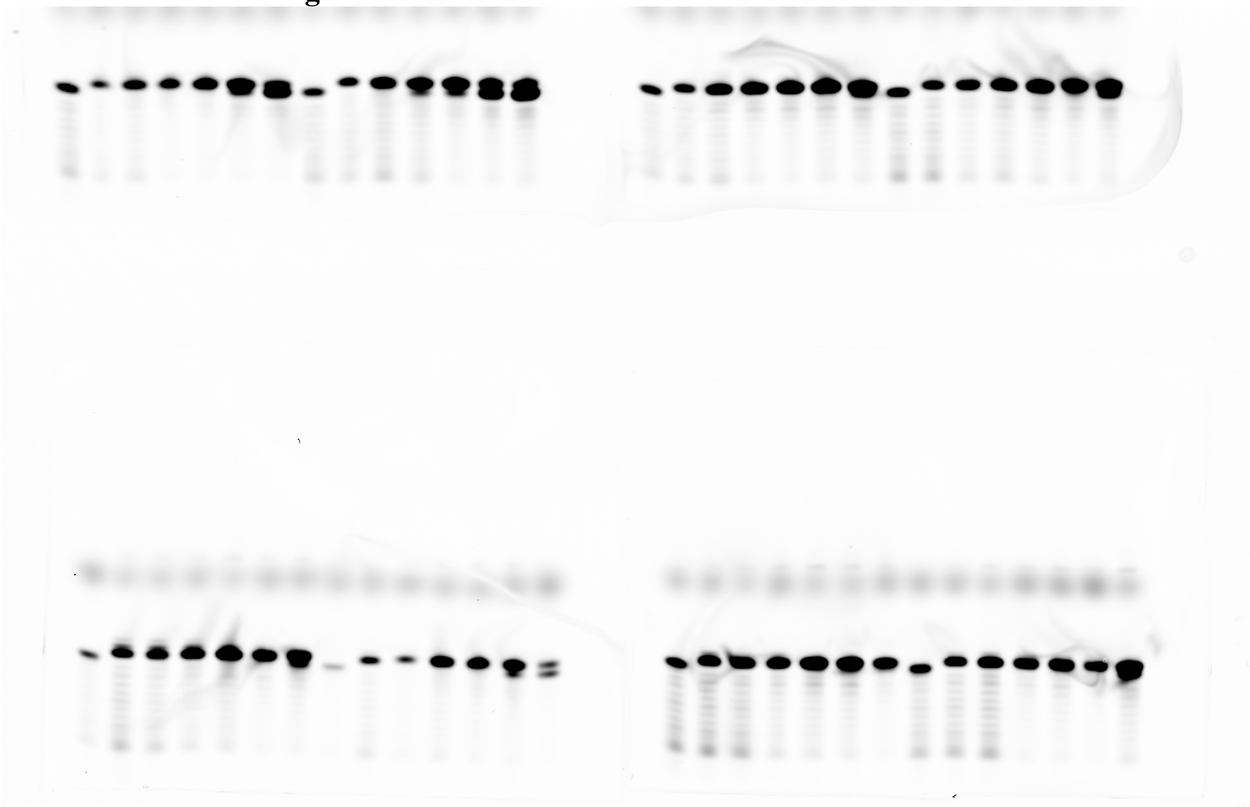

Unmodified Gel for Fig. S12

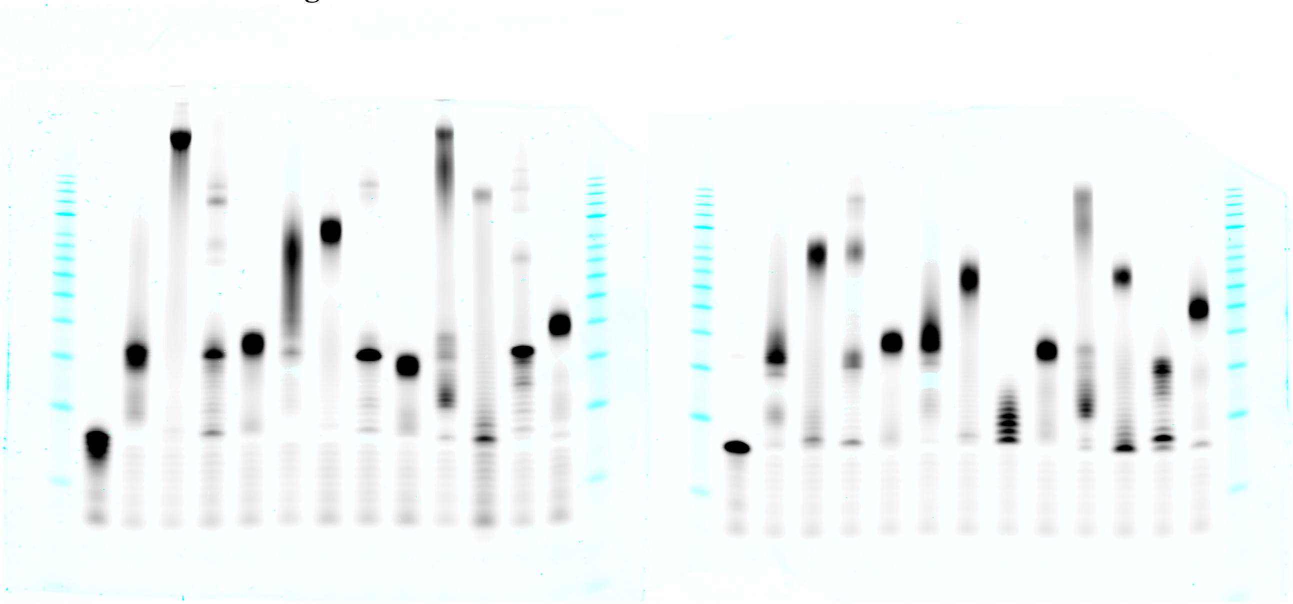

Unmodified Gel for Fig. S13

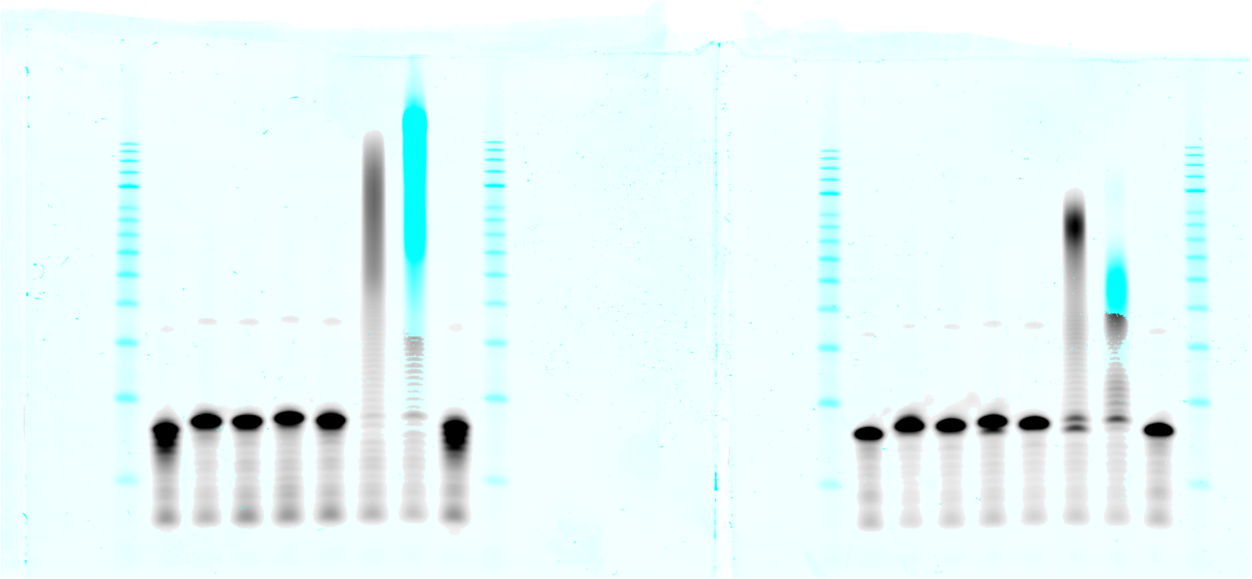

Unmodified Gel for Fig. S15

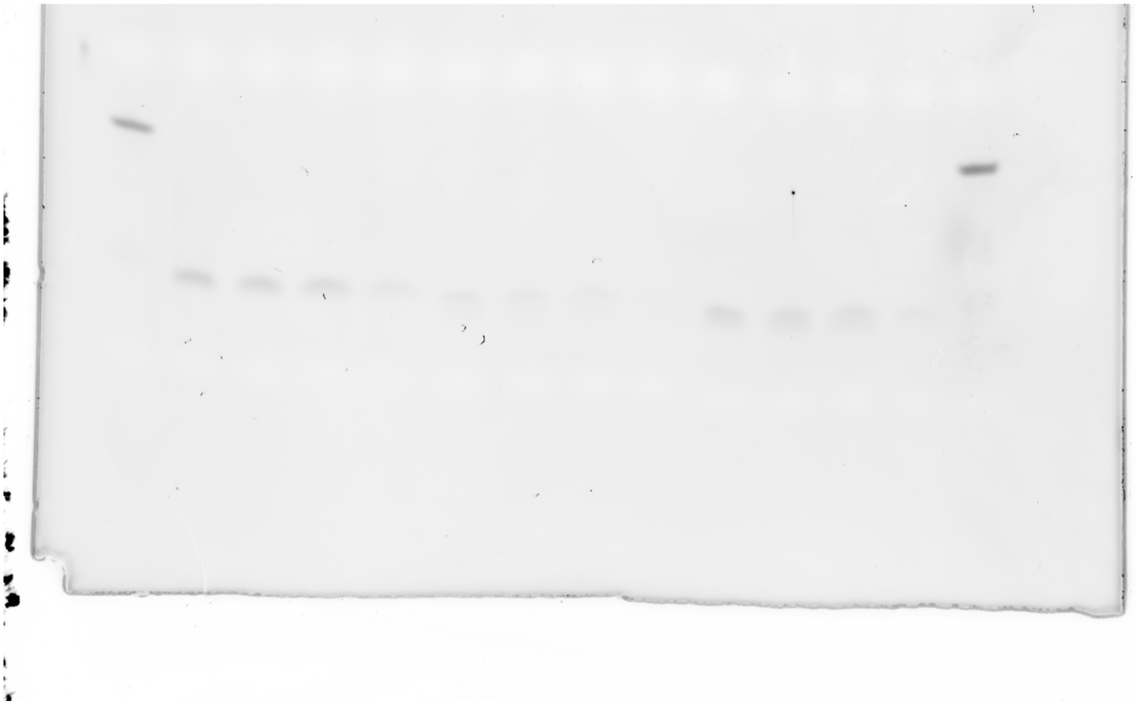

Unmodified Gel for Fig. S17B (Cy3 Channel)

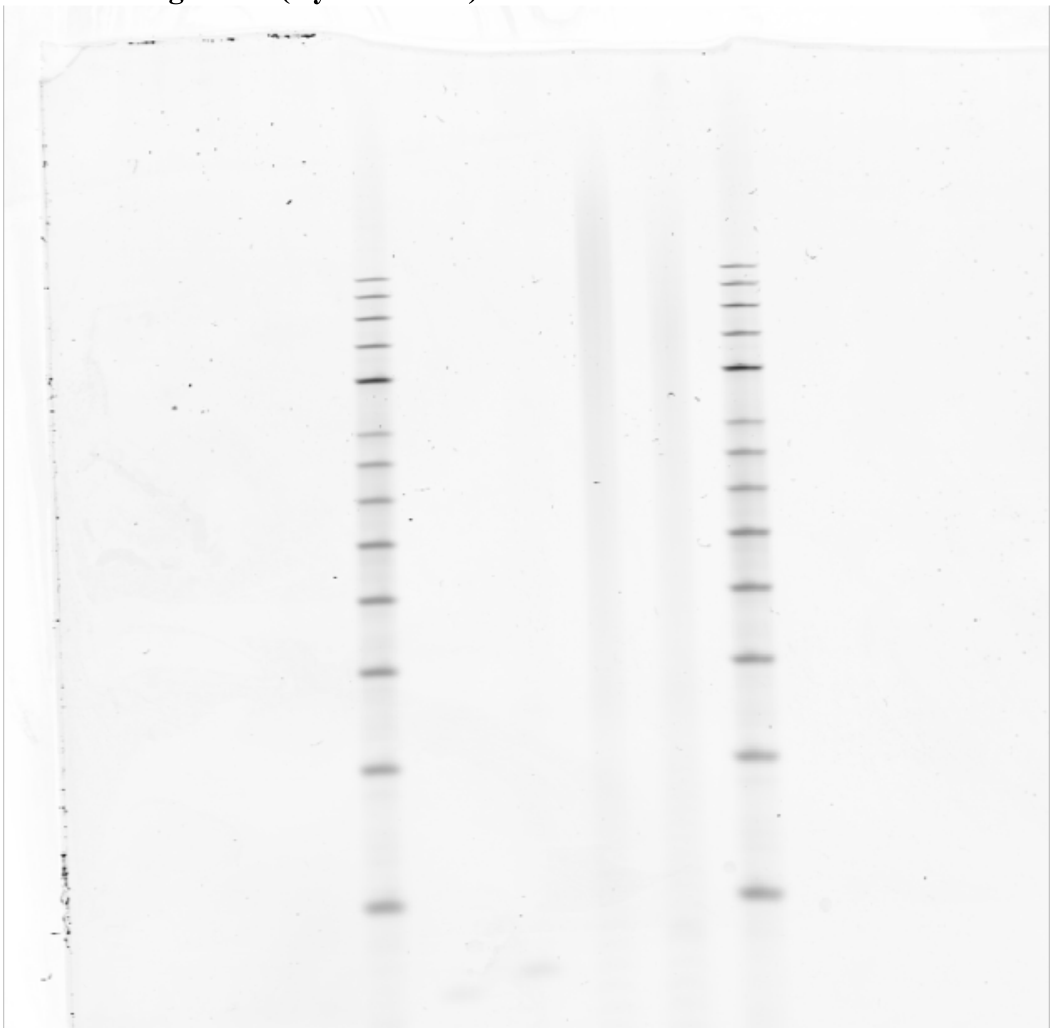

Unmodified Gel for Fig. S17B (Cy5 Channel)

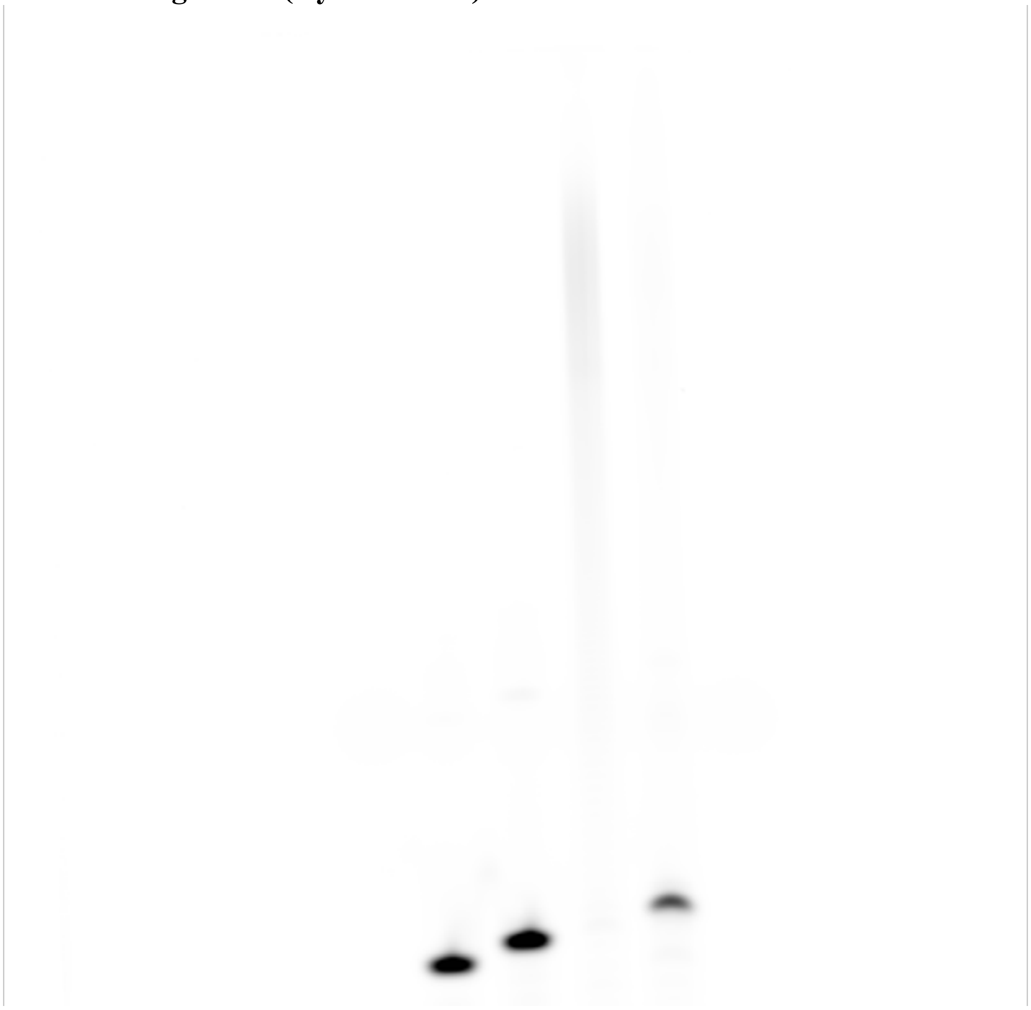

Unmodified Gel for Fig. S20A

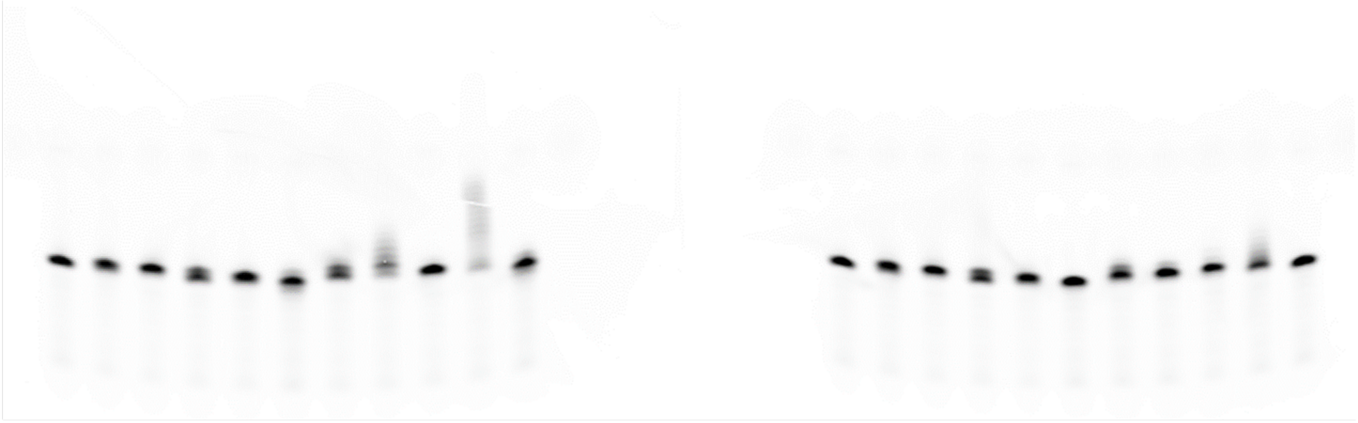

Supplement: Supplementary file 1 — Supplementary Figs. 1–21 and Tables 1–3, enzyme sequences SEQ1–SEQ5, NMR analysis of 3′-O-allyl ether NTP and uncropped scans of gels from Supplementary figures. [file 41587_2024_2244_MOESM1_ESM.pdf]
