# Supplementary material for: Template-independent enzymatic synthesis of RNA oligonucleotides
Source: Nat Biotechnol. 2024 Jul 12;43(5):762–72. doi: 10.1038/s41587-024-02244-w (PMC12084152; doi:10.1038/s41587-024-02244-w)
Supplement: Supplementary file 2 — Reporting Summary [file 41587_2024_2244_MOESM2_ESM.pdf]

nature portfolio

Corresponding author(s): Erkin Kuru, George Church

Last updated by author(s): Mar 17, 2023

Reporting Summary

Nature Portfolio wishes to improve the reproducibility of the work that we publish. This form provides structure for consistency and transparency in reporting. For further information on Nature Portfolio policies, see our Editorial Policies and the Editorial Policy Checklist.

Statistics

For all statistical analyses, confirm that the following items are present in the figure legend, table legend, main text, or Methods section.

n/a	Confirmed	
☒	☐	The exact sample size (*n*) for each experimental group/condition, given as a discrete number and unit of measurement
☒	☐	A statement on whether measurements were taken from distinct samples or whether the same sample was measured repeatedly
☒	☐	The statistical test(s) used AND whether they are one- or two-sided<br>*Only common tests should be described solely by name; describe more complex techniques in the Methods section.*
☒	☐	A description of all covariates tested
☒	☐	A description of any assumptions or corrections, such as tests of normality and adjustment for multiple comparisons
☒	☐	A full description of the statistical parameters including central tendency (e.g. means) or other basic estimates (e.g. regression coefficient) AND variation (e.g. standard deviation) or associated estimates of uncertainty (e.g. confidence intervals)
☒	☐	For null hypothesis testing, the test statistic (e.g. F, t, r) with confidence intervals, effect sizes, degrees of freedom and P value noted<br>*Give P values as exact values whenever suitable.*
☒	☐	For Bayesian analysis, information on the choice of priors and Markov chain Monte Carlo settings
☒	☐	For hierarchical and complex designs, identification of the appropriate level for tests and full reporting of outcomes
☒	☐	Estimates of effect sizes (e.g. Cohen's *d*, Pearson's *r*), indicating how they were calculated

Our web collection on statistics for biologists contains articles on many of the points above.

Software and code

Policy information about availability of computer code

Data collection	MALDI-TOF mass spectrometry data was collected using a Bruker Daltronics autoflex system using the manufacturer's supplied software (FlexControl, version 3.4). LC/MS data was collected using an Agilent 1200 Series LC and 6400 Series single-quad mass spectrometer using manufacturer's supplied software (OpenLab ChemStation). Gel electrophoresis images were acquired and processed using an Azure Biosystems Sapphire Biomolecular Imager using manufacturer’s supplied software (Sapphire Capture).
Data analysis	All MALDI-TOF mass spectrometry data was analyzed and exported using the manufacturer's supplied software (FlexAnalysis, version 3.4). LC/MS data was analyzed using the manufacturer's supplied software (OpenLab ChemStation) with the Agilent Deconvolution & Bioanalysis Software add-on. The masses (m/z) of oligonucleotide intermediates, products, and major impurities are reported and assessed using a custom Microsoft Excel-based toolkit (See ezRNA Analyzer tab in Supplementary_Data_1). This was particularly relevant for mass spectrometry using negative mode MALDI-TOF. The toolkit sums up the exact masses of all individual bases and modifications in the oligonucleotide sequences. We found the exact masses for small molecules and building blocks using structural information obtained using ChemDraw (version 23.0.1).

For manuscripts utilizing custom algorithms or software that are central to the research but not yet described in published literature, software must be made available to editors and reviewers. We strongly encourage code deposition in a community repository (e.g. GitHub). See the Nature Portfolio guidelines for submitting code & software for further information.

Data

Policy information about availability of data

All manuscripts must include a data availability statement. This statement should provide the following information, where applicable:

- Accession codes, unique identifiers, or web links for publicly available datasets
- A description of any restrictions on data availability
- For clinical datasets or third party data, please ensure that the statement adheres to our policy

Raw NMR data are deposited at https://doi.org/10.7910/DVN/8XLE6P. Processed MALDI-TOF mass spec data can be accessed at https://github.com/dan-wiegand/Enzymatic_RNA_Synthesis. Additional LC/MS data are available upon request from the corresponding authors. Additional data are available in the Article, Online methods, and Supplementary information. To reproduce the results, no further data is needed.

Human research participants

Policy information about studies involving human research participants and Sex and Gender in Research.

Reporting on sex and gender	N/A
Population characteristics	N/A
Recruitment	N/A
Ethics oversight	N/A

Note that full information on the approval of the study protocol must also be provided in the manuscript.

Field-specific reporting

Please select the one below that is the best fit for your research. If you are not sure, read the appropriate sections before making your selection.

☒ Life sciences ☐ Behavioural & social sciences ☐ Ecological, evolutionary & environmental sciences

For a reference copy of the document with all sections, see nature.com/documents/nr-reporting-summary-flat.pdf

Life sciences study design

All studies must disclose on these points even when the disclosure is negative.

Sample size	No statistical methods were used to predetermine sample size because this does not apply to this study. Enzymatically synthesized oligonucleotide products are reported when the expected (calculated) m/z matched the observed m/z within the accuracy limitations of the instrument used (MALDI-TOF or LC/MS).
Data exclusions	No data was excluded for this study.
Replication	In general, large batches of raw materials used in this study (enzymes, NTP building blocks, labeled CPG solid support) were produced, aliquoted into smaller amounts and stored appropriately following protocols described in the methods section. We did not experience significant sample to sample variability with these materials, which indicated good replication of experimental data as acquired through MALDI-TOF mass spectrometry, LC/MS and gel electrophoresis.
Randomization	Randomization of samples was not applicable to this study as we demonstrate achieving the enzymatic synthesis of specific intermediates and final products (as reported by MALDI-TOF, LC/MS, and gel electrophoresis) rather than comparing experimental groups.
Blinding	Investigators were not blinded in this study as no groups were tested.

Reporting for specific materials, systems and methods

We require information from authors about some types of materials, experimental systems and methods used in many studies. Here, indicate whether each material, system or method listed is relevant to your study. If you are not sure if a list item applies to your research, read the appropriate section before selecting a response.

Materials & experimental systems

n/a	Involved in the study
☒	☐ Antibodies
☒	☐ Eukaryotic cell lines
☒	☐ Palaeontology and archaeology
☒	☐ Animals and other organisms
☒	☐ Clinical data
☒	☐ Dual use research of concern

Methods

n/a	Involved in the study
☒	☐ ChIP-seq
☒	☐ Flow cytometry
☒	☐ MRI-based neuroimaging
