# Supplementary figures and images for: Template-independent enzymatic synthesis of RNA oligonucleotides
Source: Nat Biotechnol. 2024 Jul 12;43(5):762–72. doi: 10.1038/s41587-024-02244-w (PMC12084152; doi:10.1038/s41587-024-02244-w)

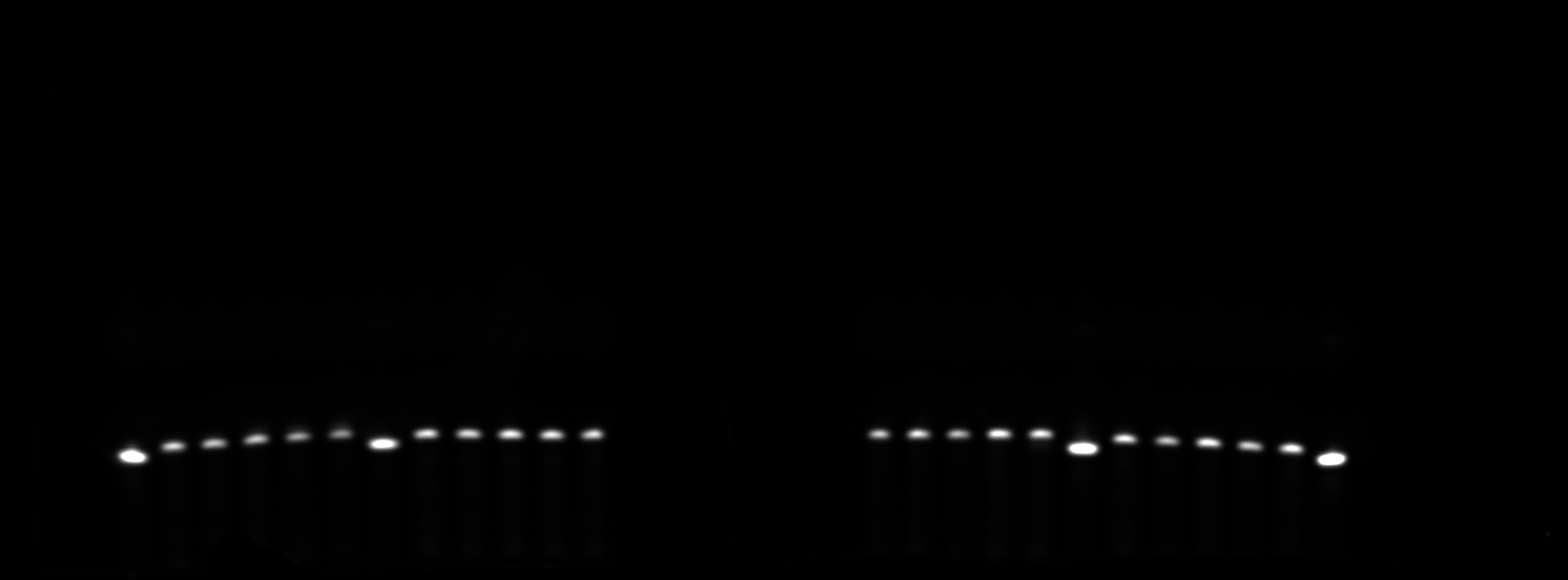

Supplement: Supplementary file 4 — Kinetic profile for each 3′-O-allyl ether NTP analyzed with denaturing gel electrophoresis; reaction samples were taken at 1, 5, 10, 20 and 30 min. Control reactions (N) included all reaction components except NTP. [file 41587_2024_2244_MOESM4_ESM.tif]

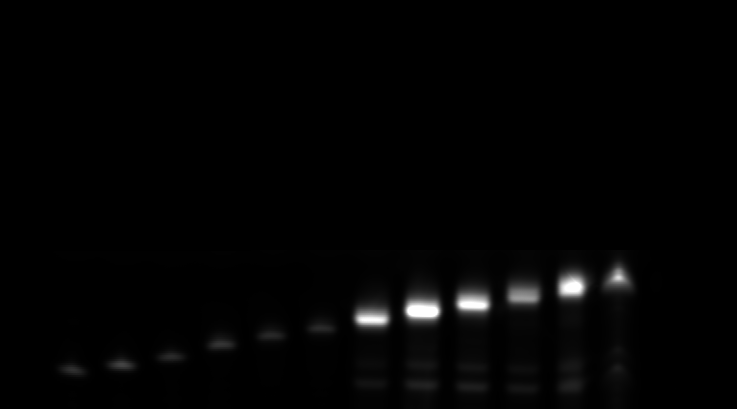

Supplement: Supplementary file 5 — High-resolution gel electrophoresis to analyze the success of each cycle after the sequence was enzymatically synthesized with an imager set to collect Cy5 signal. [file 41587_2024_2244_MOESM5_ESM.tif]
